# Structurally Constrained Stibenium: Metallomimetic C−Si Bond Activation

**DOI:** 10.1002/anie.3059944

**Published:** 2026-05-01

**Authors:** Donia Toami, Roman Dobrovetsky

**Affiliations:** ^1^ School of Chemistry, Raymond and Beverly Sackler Faculty of Exact Sciences Tel Aviv University Tel Aviv Israel

**Keywords:** antimony, cations, metallomimetic catalysis, silane, small‐molecule activation

## Abstract

The chemistry of structurally constrained pnictogen centers continues to attract interest within the field of metallomimetic main‐group catalysis. Herein, we report a structurally constrained stibenium cation [**1**]^+^ supported by a 2,6‐bis(o‐carborano)pyridine pincer‐type ligand. This unique Sb‐based cation exhibits an unprecedented ability to activate the Si−C bonds via oxidative addition not only in hydrosilanes but also in otherwise unreactive tetraalkylsilanes. This remarkable reactivity of [**1**]^+^ enables its application as a catalyst for silane redistribution under mild conditions. Experimental and density functional theory (DFT) mechanistic studies of this catalysis suggest that [**1**]^+^ operates in a metallomimetic fashion, involving key steps commonly associated with transition‐metal catalysis.

## Graphical abstract

A structurally constrained stibenium cation supported by a 2,6‐bis(o‐carborano)pyridine pincer‐type ligand was prepared, and its preliminary reactivity toward silanes was investigated. Remarkably, Si−C bonds in silanes undergo activation via an oxidative‐addition‐type process at the cationic Sb center. This transformation enables catalytic silane redistribution through a metallomimetic Sb^III^/Sb^V^ redox cycle.

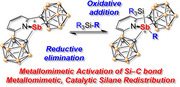

## 1 Introduction

The redox ability of pnictogens (Pn), elements of group 15 (P, As, Sb, Bi), enables them to mimic transition‐metal chemistry in bond activation and catalysis, by switching between two stable oxidation states (Pn^n^ ⇌ Pn^n+2^) [1, 2]. The strategy of enforcing structural constraints on pnictogen centers by incorporating them into rigid ligands has proven successful in enhancing their reactivity by distorting their geometry away from ideal VSEPR shapes, which induces rehybridization at the central atom and thereby lowers the HOMO‐LUMO gap [3, 4]. The reactivity of these structurally constrained pnictogen centers can be further enhanced by incorporating a cationic character, which lowers the LUMO energy and increases their electrophilicity [5].

Sb^III^ centers generally possess a lower‐lying LUMO compared to other pnictogens, due to their lower electronegativity and higher polarizability, which inherently makes them highly Lewis acidic [6, 7, 8, 9, 10, 11, 12]. Zhou recently demonstrated that Sb^III^ complexes are inherently more ambiphilic, both nucleophilic and electrophilic, compared to other pnictogens [13]. These features suggest that enforcing structural constraints on Sb centers can significantly enhance their reactivity toward small‐molecule activation and open new synthetic avenues. Despite this potential, only a few examples of geometrically constrained Sb^III^ centers have been reported [14], and their reactivity with small molecules, and especially their catalytic applications, remain largely unexplored.

**FIGURE 1:**
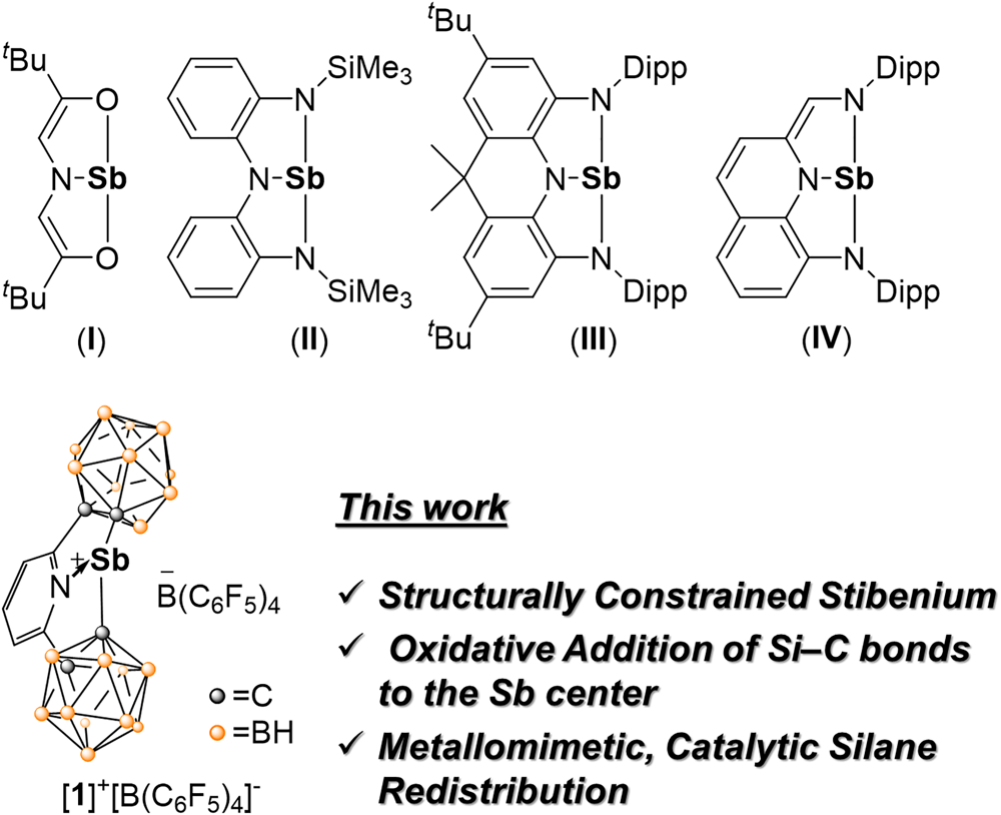
Previously reported geometrically constrained stibines, and this work, structurally constrained stibenium [**1**]^+^.

The first structurally constrained Sb^III^ center supported by an ONO‐pincer ligand (**I**) was reported by Arduengo and co‐workers (Figure 1); however, no bond‐activation reactivity arising from the ambiphilic nature of the Sb center was demonstrated [15]. In 2019, Chitnis and co‐workers reported a structurally constrained stibine (**II**) featuring a trianionic NNN pincer‐type ligand (Figure 1). Compound **II** was isolated as a dimer in the solid state but was shown to exist as a monomer in solution [16]. Again, no reactivity with small molecules was reported. Wang and co‐workers isolated T‐shaped stibine (**III**) supported by a sterically encumbered triamide NNN ligand (Figure 1). **III** could be reduced to afford a stable radical anion, which was found to be reactive toward S_8_, affording a salt of the S_10_^−2^ dianion [17]. Very recently, Hwang used an elegant ligand design for the synthesis of the T‐shaped stibine in a redox‐active NNN‐type ligand (**IV**) (Figure 1) and showed the activation of O_2_ and oxygen atom transfer in 2e redox process [18].

Similarly to neutral Sb‐based species, Sb‐based cations have attracted increasing attention due to their potential in bond activation and catalysis. Nevertheless, only a handful of Sb^III^ cations have been reported [7, 8], and their reactivity remains largely underexplored. The number of structurally constrained Sb^III^ cations is even more limited, with only two examples described to date [19, 20]. In 2022, Greb reported a Lewis superacidic calix[4]pyrrolato stibenium [19], and in 2025, Gilliard reported a tricationic Sb^III^ species supported by a neutral, tridentate pincer‐type ligand, which displayed reactivity toward C−H bond activation in terminal alkynes in the presence of an external base [20]. More recently, we reported a distorted cationic Sb^III^ center supported by an OCO‐pincer ligand, which enabled the direct preparation of a cationic P^III^‐compound via Sb‐to‐P metathesis reaction [21].

In contrast to Sb^I^ species, whose ambiphilicity has been demonstrated and exploited for small‐molecule activation and catalysis [14, 22], the ambiphilic reactivity of Sb^III^ compounds is rare and, to the best of our knowledge, has only been observed when driven primarily by oxophilicity [11, 12, 13]. This limited ambiphilic behavior of Sb^III^ centers stems primarily from the inertness of their lone pair, which renders them largely non‐nucleophilic [16, 21, 23, 24]. Importantly, previous studies have shown that complexes bearing pincer ligands with heteroatoms coordinated to the central Sb^III^ center often tend to dimerize due to strain around the antimony center, ultimately quenching its reactivity.

We recently reported a structurally constrained, ambiphilic phosphenium with a 2,6‐bis(o‐carborano)pyridine pincer‐type ligand, which was capable of activating the H−H bond at a single P^III^ center [25]. Motivated by this success, we decided to attempt the synthesis of a stibenium analog of this molecule, [**1**]^+^ (Figure 1), and investigate its potential reactivity with small molecules and its utility in catalysis. Herein, we report the synthesis of [**1**]^+^[B(C_6_F_5_)_4_]^−^ and its distinct reactivity compared to its phosphenium predecessor. Unexpectedly, we found that [**1**]^+^ activates Si−C bonds through an unprecedented oxidative‐addition‐type (OA) reaction at the cationic Sb center across a range of silanes (R_n_SiH_4‐n_: R = Alk, Ar) and serves as a highly efficient catalyst for silane redistribution. Experimental and DFT studies suggest that [**1**]^+^ engages in catalysis in a metallomimetic manner.

## 2 Results and Discussion

**SCHEME 1:**
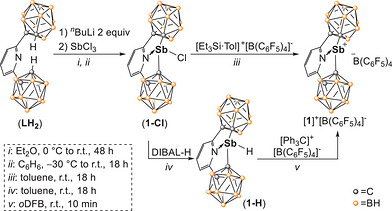
Synthesis of [**1**]^+^[B(C_6_F_5_)_4_]^−^.

**FIGURE 2:**
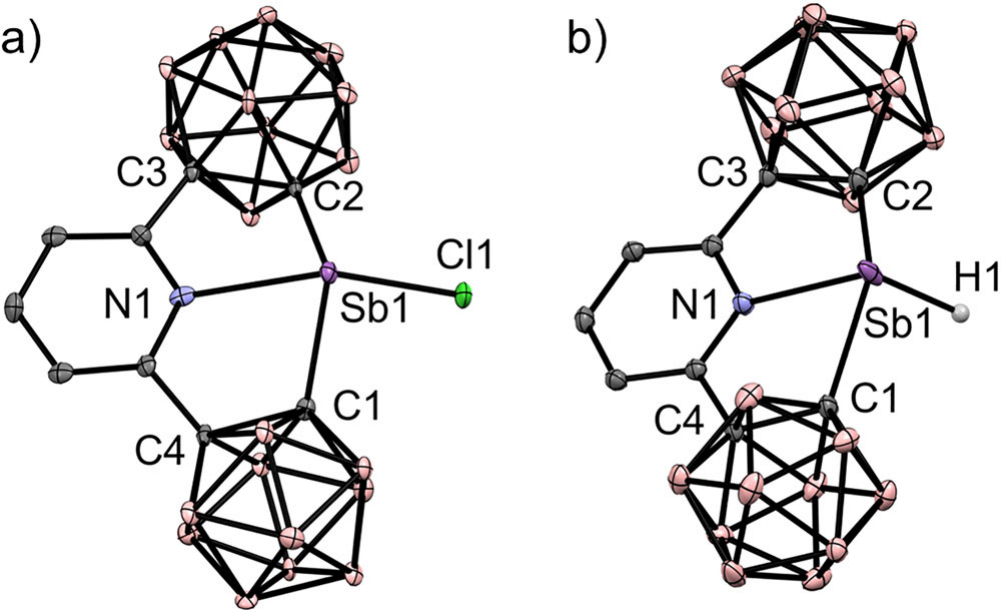
POV‐ray depiction of **1‐Cl** (a), and **1‐H** (b). Thermal ellipsoids at 30% probability; non‐relevant hydrogen atoms were omitted for clarity.

The synthetic route toward [**1**]^+^[B(C_6_F_5_)_4_]^−^ closely followed the procedure we previously employed for the preparation of the analogous phosphenium [25]. In the first step, the **LH_2_** ligand [26] was deprotonated with two equivalents of *^n^* BuLi, followed by reaction with SbCl_3_ to afford the chlorostibine **1‐Cl** (Scheme 1). **1‐Cl** was purified by slow evaporation from a hexane:CH_2_Cl_2_ (1:4) mixture, yielding crystalline material in 56% yield. The molecular structure of **1‐Cl** was confirmed by single‐crystal x‐ray diffraction (SCXRD) and is shown in Figure 2a.

To obtain the desired [**1**]^+^[B(C_6_F_5_)_4_]^−^, **1‐Cl** was reacted with [Et_3_Si·Tol]^+^[B(C_6_F_5_)_4_]^−^. Multinuclear (^1^H, ^13^C, ^11^B, and ^19^F) NMR spectra were recorded after 18 h at r. t. As expected, no significant changes were observed in the ^1^H NMR spectrum. The ^11^B and ^19^F NMR spectra confirmed the presence of [B(C_6_F_5_)_4_]^−^, while the most indicative changes appeared in the ^13^C NMR spectrum: the two resonances corresponding to the *o*Cb carbons in **1‐Cl** at *δ* = 72.85 and 77.86 ppm disappeared, and two new signals were observed at *δ* = 75.84 and 79.12 ppm. Furthermore, high‐resolution mass spectrometry (HRMS) using the atmospheric pressure photoionization (APPI) supported the formation of [1]^+^ (Calculated for C_9_H_23_^10^B_8_^11^B_12_N^121^Sb: 478.3020 [M]^+^; Obs: 478.3011).

All attempts to crystallize [**1**]^+^[B(C_6_F_5_)_4_]^−^ were unsuccessful due to its high reactivity toward halogenated solvents. [**1**]^+^[B(C_6_F_5_)_4_]^−^ rapidly reacted with CH_2_Cl_2_ and with CHCl_3_ within minutes, regenerating **1‐Cl**. While this reactivity supports the formation of [**1**]^+^[B(C_6_F_5_)_4_]^−^, it also precludes crystallization from these solvents and significantly limits the range of solvents compatible with its handling. The compound also slowly decomposed in 1,2‐difluorobenzene (*o*DFB), which similarly hindered crystallization efforts.

To further verify the formation of [**1**]^+^[B(C_6_F_5_)_4_]^−^, we pursued an independent synthetic route via the corresponding hydrostibine, **1‐H** (Scheme 1), followed by hydride abstraction using [Ph_3_C]^+^[B(C_6_F_5_)_4_]^−^. This approach offered the advantage of allowing the reaction progress to be readily monitored by ^1^H NMR spectroscopy, facilitating confirmation of [**1**]^+^[B(C_6_F_5_)_4_]^−^ formation. Compound **1‐H** was obtained by reacting **1‐Cl** with DIBAL‐H and was isolated in 65% yield by crystallization from benzene at r.t. Its molecular structure was confirmed by SCXRD (Figure 2b).

Notably, isolable hydrostibines are relatively rare compared to their lighter analogues [27, 28, 29, 30, 31, 32, 33, 34], making the isolation and relative stability of **1‐H** an interesting result in its own right. Moreover, **1‐H** showed similar reactivity to [**1**]^+^[B(C_6_F_5_)_4_]^−^ toward chloroalkanes, regenerating **1‐Cl**. Nevertheless, the slower reaction allowed for its multinuclear NMR characterization in CDCl_3_. Finally, hydride was abstracted from **1‐H** using [Ph_3_C]^+^[B(C_6_F_5_)_4_]^−^ (Scheme 1). ^1^H and ^13^C NMR spectra clearly showed the formation of Ph_3_CH. In addition, the ^1^H NMR signal at 11.24 ppm, corresponding to Sb‐***H*** in **1‐H** as well as the ^13^C NMR chemical shifts of the *o*Cb carbons in **1‐H** at *δ* = 66.65 and 71.34 ppm, disappeared, and two new signals corresponding to [**1**]^+^ at *δ* = 75.84 and 79.12 ppm appeared. All these spectral changes provide strong evidence of the successful formation of the desired [**1**]^+^[B(C_6_F_5_)_4_]^−^.

**FIGURE 3:**
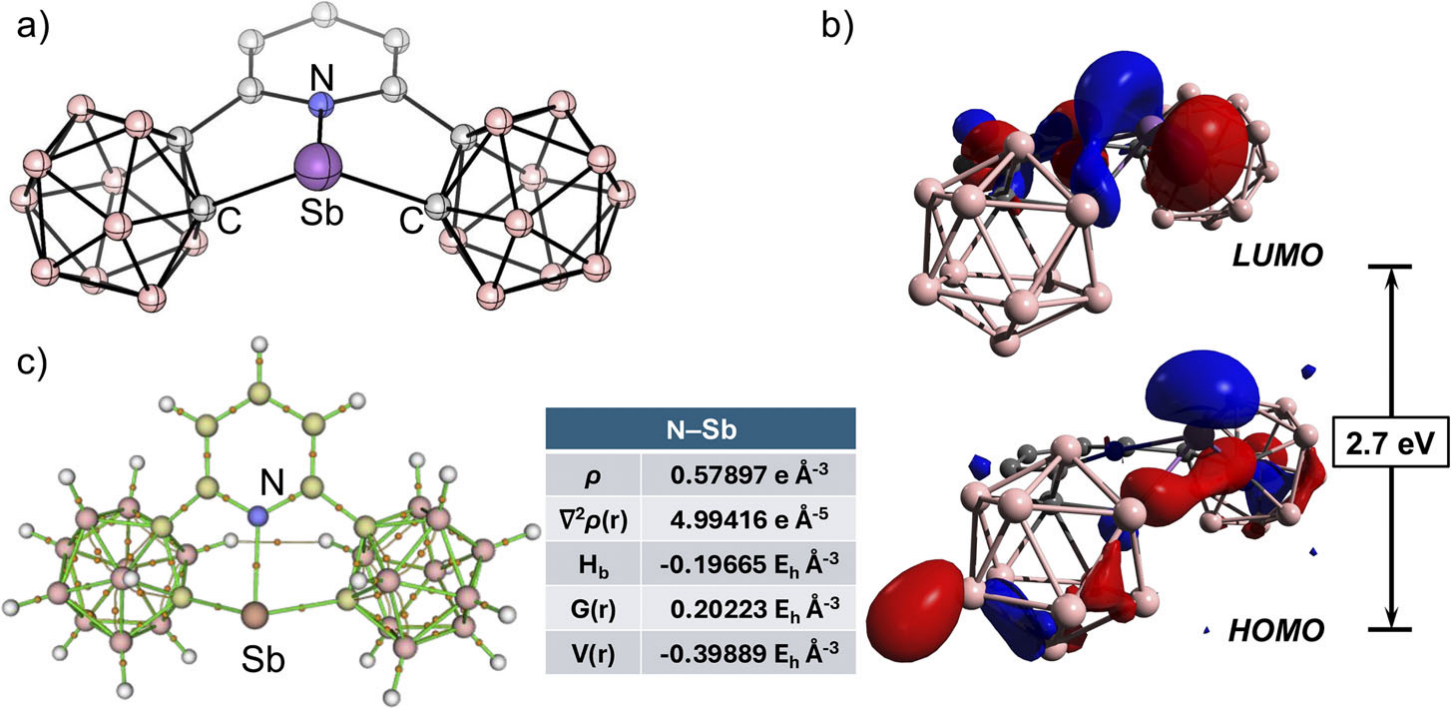
(a) Optimized structure of [**1**]^+^; (b) FMO analysis; (c) AIM analysis.

Despite the inability to obtain an experimental molecular structure of [**1**]^+^[B(C_6_F_5_)_4_]^−^, we investigated [**1**]^+^ by DFT calculations to gain insight into its structural characteristics. The structure of [**1**]^+^ was optimized in the gas phase at the BP86‐D3 level of theory [35, 36], with Ahlrichs' def2‐TZVP basis set [37], and with the relativistic effect of Sb, which was accounted for by the Stuttgart‐Dresden (SDD) effective core potential (ECP) [38, 39]. The optimized geometry around the Sb center in [**1**]^+^ corresponds to a heavily distorted trigonal pyramidal arrangement with local *C_s_* symmetry (Figure 3a) [40]. The two ∠C−Sb−N bond angles are equivalent (77.4°), while the ∠C−Sb−C bond angle is widened to 119.7°. Overall, the local geometry around the Sb approximates a cis‐divacant pseudo‐trigonal bipyramid, with the two carbon atoms occupying equatorial positions and the nitrogen atom located axially. The two Sb−C bond lengths (2.210 Å) fall within the range of typical Sb−C single bonds [40], whereas the N−Sb bond length (2.190 Å) is slightly longer than a typical N−Sb single bond [15, 16], however, yet shorter than a typical N → Sb dative bond [41, 42], likely reflecting the influence of ring strain imposed by the rigid ligand framework.

The electronic structure of [**1**]^+^ was examined using DFT calculations. Frontier molecular orbital (FMO) analysis revealed that both the HOMO and LUMO are largely localized on the Sb center, with an energy gap of 2.7 eV, sufficiently small to suggest considerable ambiphilic reactivity (Figure 3b). Natural population analysis (NPA) indicated charges of −0.51687 and +1.76767 on the N and Sb centers, respectively, consistent with a highly electron‐deficient Sb center. The computed Wiberg bond index (WBI) value of 0.4705 for the N−Sb bond indicates its polar nature. To gain deeper insight, the N−Sb bonding was analyzed using Bader's quantum theory of atoms in molecules (AIM) (Figure 3c) [43]. The bond critical point exhibited a positive Laplacian (∇^2^ *ρ*(r_BCP_) = 4.99416 e Å^−5^) and low electron density (*ρ* = 0.57897 e Å^−3^) at the N−Sb bond critical point, together with *G(r)/ρ(r)* = 0.34929 and *H(r)/ρ(r)* = −0.33965, all consistent with a donor‐acceptor interaction, defining the electronic nature of this bond.

To investigate the reactivity of [**1**]^+^[B(C_6_F_5_)_4_]^−^ toward small molecules, we began with its reaction with H_2_ to determine whether, similar to its phosphenium analog [25], it would activate the H−H bond. A J‐Young NMR tube containing [**1**]^+^[B(C_6_F_5_)_4_]^−^ in *o*DFB was pressurized with H_2_ gas (4 atm). However, in contrast to previously reported phosphenium, no oxidative addition (OA)‐type reaction between [**1**]^+^[B(C_6_F_5_)_4_]^−^ and H_2_ was observed, even after extended reaction times and heating. To rationalize this difference, DFT calculations were performed for the OA of the H−H bond to the Sb center, affording the corresponding dihydrostibonium cation. In contrast to H_2_ activation by the phosphenium (Δ*H* = −18.1 and Δ*G* = −9.4 kcal mol^−1^) [25], the process for [**1**]^+^ was found to be both an endothermic and endergonic process (Δ*H* = 5.1 and Δ*G* = 13.5 kcal mol^−1^; see ESI for details), which can be attributed to the inherently weak nature of Sb–H bonds [29, 44].

**FIGURE 4:**
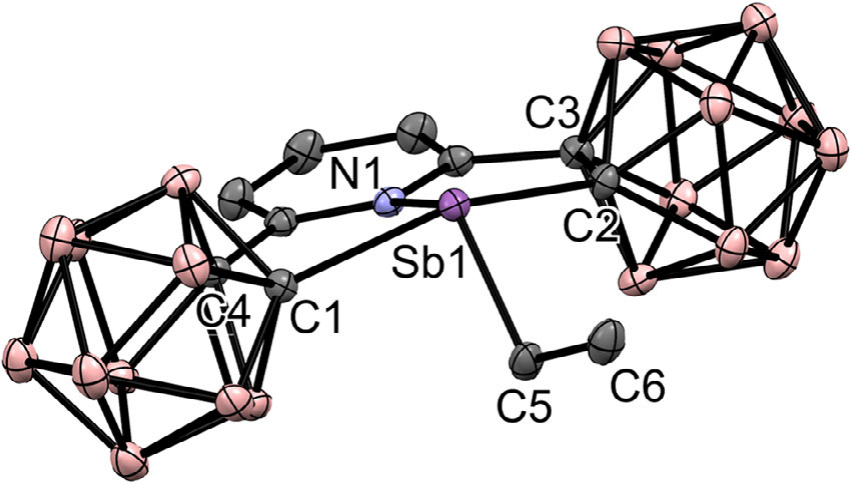
POV‐ray depiction of **1‐Et**. Thermal ellipsoids at 30% probability; hydrogen atoms were omitted for clarity.

**SCHEME 2:**
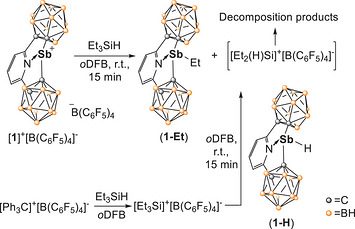
Reaction of [**1**]^+^[B(C_6_F_5_)_4_]^−^ with Et_3_SiH.

Next, the reactivity of [**1**]^+^[B(C_6_F_5_)_4_]^−^ with hydrosilanes was examined. Treatment of [**1**]^+^[B(C_6_F_5_)_4_]^−^ with Et_3_SiH in *o*DFB led, after 15 min, to complete consumption of the starting materials. The ^1^H NMR spectrum of the reaction mixture displayed two new signals, a triplet at *δ* = 1.48 ppm and a quartet at *δ* = 2.18 ppm. The ^13^C NMR spectrum showed a shift of the *o*Cb carbons from *δ* = 75.84 and 79.12 ppm to *δ* = 71.97 and 69.44 ppm, along with two new aliphatic carbons at *δ* = 10.64 and 25.70 ppm. After 48 h, crystals precipitated from the reaction mixture, and their molecular structure, determined by SCXRD, was identified as **1‐Et** (Figure 4). This unexpected outcome was striking, as it indicated activation of the more inert Si−C bond by [**1**]^+^[B(C_6_F_5_)_4_]^−^ (Scheme 2), rather than the anticipated Si−H bond activation. Closer examination of the ^19^F and ^11^B NMR spectra showed that the [B(C_6_F_5_)_4_]^−^ anion had partially decomposed to B(C_6_F_5_)_3_ (see Figures S49 and S50), possibly through the reaction with its counter cation, [Et_2_(H)Si]^+^ (Scheme 2) [45].

**FIGURE 5:**
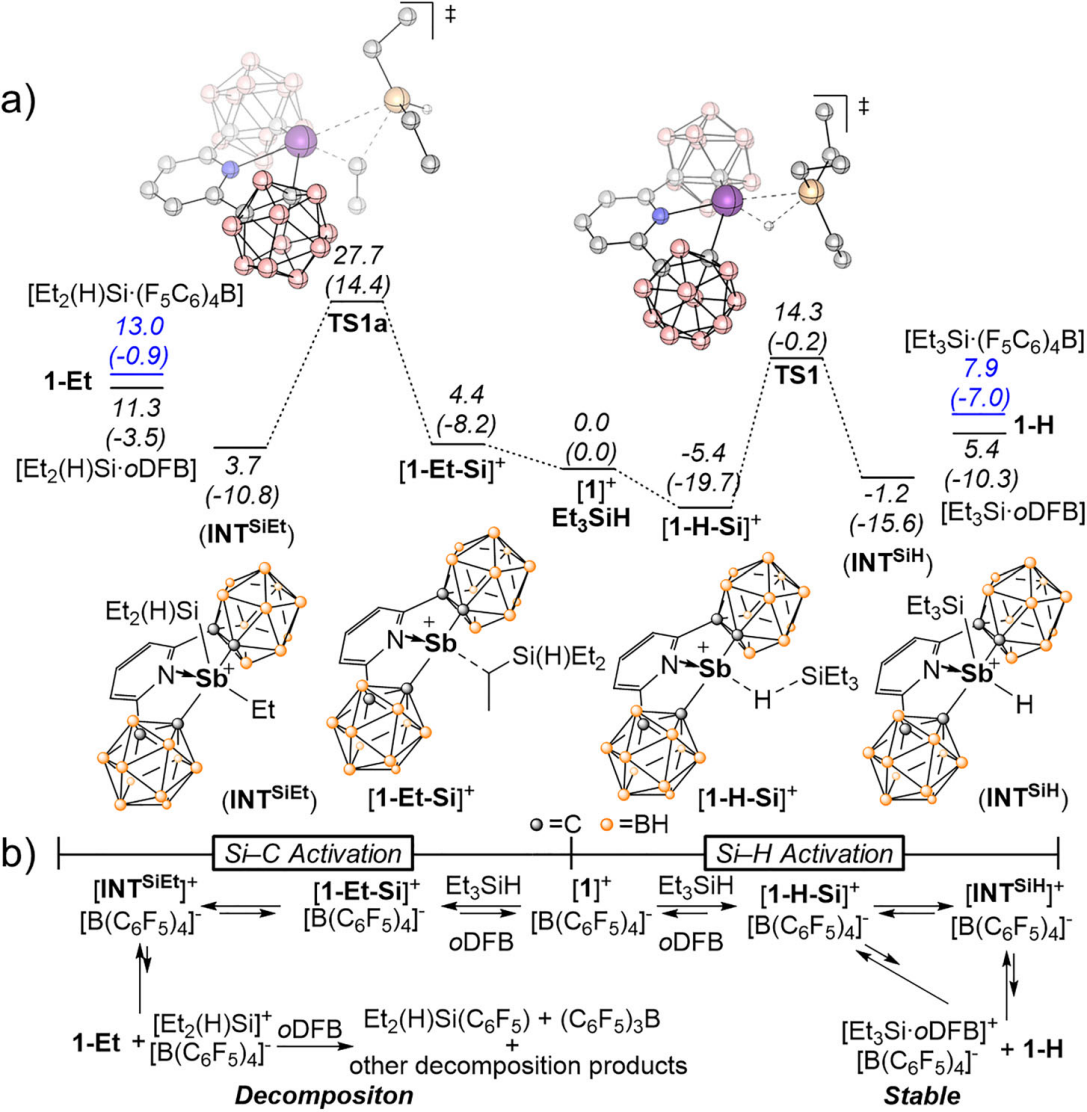
(a) DFT‐calculated mechanism for the activation of Et_3_SiH by [**1**]^+^, Δ*G*(Δ*H*) are given in kcal mol^−1^; (b) Proposed equilibrium leading to the formation of the **1‐Et**, Et_2_(H)Si(C_6_F_5_), and (C_6_F_5_)_3_B.

Unexpectedly, when **1‐H** was added to in situ‐generated [Et_3_Si]^+^[B(C_6_F_5_)_4_]^−^ in *o*DFB (Scheme 2), with the expectation of obtaining the oxidative addition (OA) product of the Si−H bond (**INT^SiH^** in Figure 5), the same products were obtained as in the direct reaction between [**1**]^+^[B(C_6_F_5_)_4_]^−^ and Et_3_SiH, namely, **1‐Et** and [B(C_6_F_5_)_4_]^−^ decomposition products. This observation suggests an equilibrium between **1‐H** and [Et_3_Si]^+^[B(C_6_F_5_)_4_]^−^ generating [**1**]^+^[B(C_6_F_5_)_4_]^−^ and Et_3_SiH, subsequently reacting to yield the observed products.

To gain deeper insight into the mechanism and assess the feasibility of OA of the Si−H and Si−C bonds to the Sb center, DFT calculations were performed at the BP86‐D3/def2‐TZVP(SDD(Sb)) level of theory (for gas‐phase calculations see ESI) [35, 36, 37, 38, 39]. Solvent effects of *o*DFB were accounted for using the conductor‐like polarizable continuum model (CPCM) through single‐point corrections [46, 47]. The adduct between the cationic Sb^III^ center in [**1**]^+^ and Et_3_SiH, affording [**1‐H‐SiEt_3_**]^+^, was computed to be exothermic and slightly exergonic (Δ*H* = −19.7 and Δ*G* = −5.4 kcal mol^−1^), representing the most favorable point on this reaction coordinate (Figure 5a). Subsequent migration of Et_3_Si^+^ to Sb, corresponding to formal OA of the Si−H bond, leading to intermediate **INT^SiH^**, is slightly uphill (ΔΔ*H* = 4.1 and ΔΔ*G* = 4.2 kcal mol^−1^) and proceeds via transition state (**TS1**) (ΔΔ*G* ^‡^ = 19.7 kcal mol^−1^) (Figure 5a). The *o*DFB‐ or [B(C_6_F_5_)_4_]^−^ ‐assisted dissociation of the [Et_3_Si]^+^ cation from either [**1‐H‐SiEt_3_**]^+^ or **INT^SiH^**, giving **1‐H** and either [Et_3_Si·*o*DFB]^+^ or zwitterionic [Et_3_Si·(C_6_F_5_)_4_B], was found to be endothermic and modestly endergonic in both cases (Figure 5a). Nevertheless, the relatively small Gibbs free energy differences (ΔΔG = 6.6 and 9.1 kcal mol^−1^ for the formation of [Et_3_Si·*o*DFB]^+^ and [Et_3_Si·(C_6_F_5_)_4_B], respectively) suggest that this step is reversible, with the equilibrium favoring [**1‐H‐SiEt_3_**]^+^ or **INT^SiH^** (Figure 5b). The overall energetics thus suggest that activation of the Si−H bond by [**1**]^+^ is a potentially reversible process (Figure 5b).

In contrast, adduct formation through the ethyl group, producing [**1‐Et‐Si(H)Et_2_**]^+^, is less favorable, being exothermic (Δ*H* = −8.2 kcal mol^−1^) and slightly endergonic (Δ*G* = 4.4 kcal mol^−1^). The subsequent migration of [Et_2_(H)Si]^+^ to Sb, corresponding to OA of the Si−C bond, leading to **INT^SiEt^**, was calculated to be marginally exothermic and exergonic (ΔΔ*H* = −2.0 and ΔΔ*G* = −0.7 kcal mol^−1^), proceeding via **TS1a** (ΔΔ*G* ^‡^ = 23.3 kcal mol^−1^) (Figure 5a). Expectedly, the *o*DFB or [B(C_6_F_5_)_4_]^−^ ‐assisted dissociation of the [Et_2_(H)Si]^+^ cation from **INT^SiEt^** forming **1‐Et** and either [Et_2_(H)Si·*o*DFB]^+^ or [Et_2_(H)Si·(C_6_F_5_)_4_B], was calculated to be slightly more endergonic (ΔΔ*G* = 7.6 and 9.3 kcal mol^−1^, respectively) compared to the Si−H pathway (Figure 5a), indicating that this equilibrium is also accessible. Importantly, the in situ‐generated [Et_2_(H)Si]^+^[B(C_6_F_5_)_4_]^−^ in *o*DFB is experimentally unstable and undergoes irreversible decomposition to afford, among other unidentifiable products, (C_6_F_5_)_3_B and Et_2_(H)Si(C_6_F_5_) (see ESI Figures S53–S55 for more details) [45], effectively driving the equilibrium toward **1‐Et** formation (Figure 5b).

Taken together, the computational and experimental data support the notion that the Si−C OA pathway to the Sb center is accessible. However, the instability and subsequent decomposition of [Et_2_(H)Si]^+^[B(C_6_F_5_)_4_]^−^ salt render the overall reaction irreversible, favoring the formation of the observed product **1‐Et** (Scheme 2). Importantly, to the best of our knowledge, the OA of the Si−C bond to main‐group centers has not been reported to date.

**SCHEME 3:**
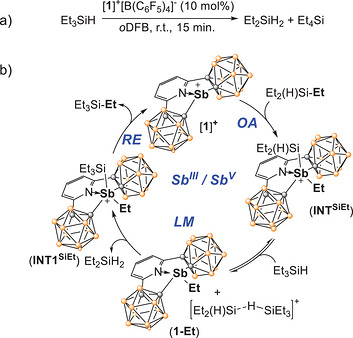
[**1**]^+^[B(C_6_F_5_)_4_]^−^ catalyzed redistribution of Et_3_SiH.

Remarkably, when [**1**]^+^[B(C_6_F_5_)_4_]^−^ was reacted with an excess of Et_3_SiH (10 equiv) in *o*DFB, complete consumption of Et_3_SiH was observed within 15 min by both ^1^H and ^29^Si NMR spectroscopy. The formation of Et_4_Si and Et_2_SiH_2_ was detected, indicating that [**1**]^+^[B(C_6_F_5_)_4_]^−^ acts as a catalyst for the silane redistribution reaction (Scheme 3a). Notably, the silane redistribution process is an important synthetic tool that provides an elegant route to various silanes, is used for ring‐opening polymerization of cyclic organosilanes and is employed in the formation of silicon‐containing bicyclic compounds [48, 49, 50, 51, 52, 53, 54, 55, 56, 57, 58].

We propose that [**1**]^+^[B(C_6_F_5_)_4_]^−^ catalyzes the redistribution of Et_3_SiH via the following metallomimetic mechanism. In the initial step, OA of the Et_2_(H)Si−Et bond to the Sb center in [**1**]^+^ occurs, generating intermediate **INT^SiEt^**. This is followed by an Et_3_SiH‐assisted dissociation of the [Et_2_(H)Si]^+^ cation from **INT^SiEt^**, leading to the formation of **1‐Et** and [Et_2_(H)Si‐H‐SiEt_3_]^+^[B(C_6_F_5_)_4_]^−^. The latter species subsequently dissociates to give intermediate **INT1^SiEt^** and Et_2_SiH_2_, one of the observed products. **INT1^SiEt^** then undergoes a reductive elimination (RE) step, affording Et_4_Si as the second product of the reaction and regenerating the catalyst (Scheme 3b).

**FIGURE 6:**
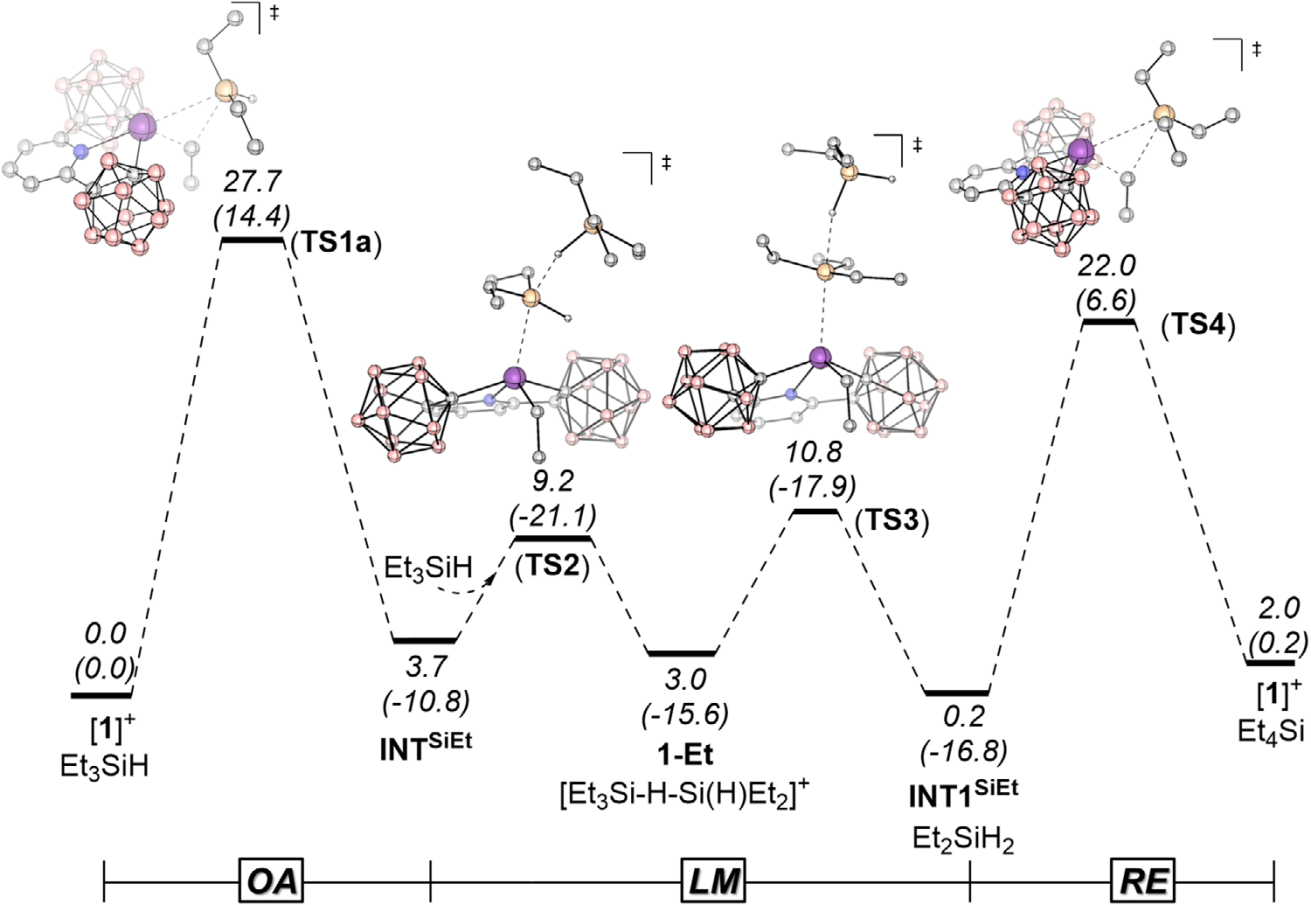
DFT‐calculated mechanism for the redistribution of Et_3_SiH catalyzed by [**1**]^+^, Δ*G* (Δ*H*) are given in kcal mol^−1^.

To support the proposed mechanism, DFT calculations at BP86‐D3/def2‐TZVP(SDD(Sb)) level of theory (for gas‐phase calculations, see ESI) with a CPCM(*o*DFB) single‐point correction, were carried out [35, 36, 37, 38, 39, 46, 47]. The first step, the OA of the Et_2_(H)Si−Et to the Sb center, was described above (Figure 5) and is calculated to be exothermic and slightly endergonic (Δ*H* = −10.8 and Δ*G* = 3.7 kcal mol^−1^) proceeding through **TS1a** (Δ*G* ^‡^ = 27.7 kcal mol^−1^). The subsequent Et_3_SiH‐assisted dissociation of the Et_2_(H)Si^+^ cation, yielding **1‐Et** and [Et_2_(H)Si‐H‐SiEt_3_]^+^, is slightly exothermic and Gibbs free energy neutral (ΔΔ*H* = −4.8 and ΔΔ*G* = −0.7 kcal mol^−1^), indicating that this step is reversible. The energy barrier for this step (**TS2**) was calculated to be ΔΔ*G* ^‡^ = 5.5 kcal mol^−1^. Dissociation of [Et_2_(H)Si‐H‐SiEt_3_]^+^ to Et_2_SiH_2_ and **INT1^SiEt^** is enthalpy neutral and slightly exergonic (ΔΔ*H* = −1.2 and ΔΔ*G* = −2.8 kcal mol^−1^) with a Gibbs free energy barrier (**TS3**) of ΔΔ*G* ^‡^ = 7.8 kcal mol^−1^, also consistent with an equilibrium process. The RE of Et_4_Si from **INT1^SiEt^** is endothermic and marginally endergonic (ΔΔ*H* = 17.0 and ΔΔ*G* = 1.8 kcal mol^−1^) with a calculated barrier of ΔΔ*G* ^‡^ = 21.8 kcal mol^−1^ (Figure 6). The relatively high ΔΔH of this final step can be rationalized by noting that, under catalytic conditions, [**1**]^+^ would rapidly react with Et_3_SiH, thereby driving the reaction forward. Importantly, the overall redistribution reaction is thermoneutral (Δ*H* = 0.2 and Δ*G* = 2.0 kcal mol^−1^), implying that, as long as [**1**]^+^ is “alive,” a statistical mixture of products is expected.

**FIGURE 7:**
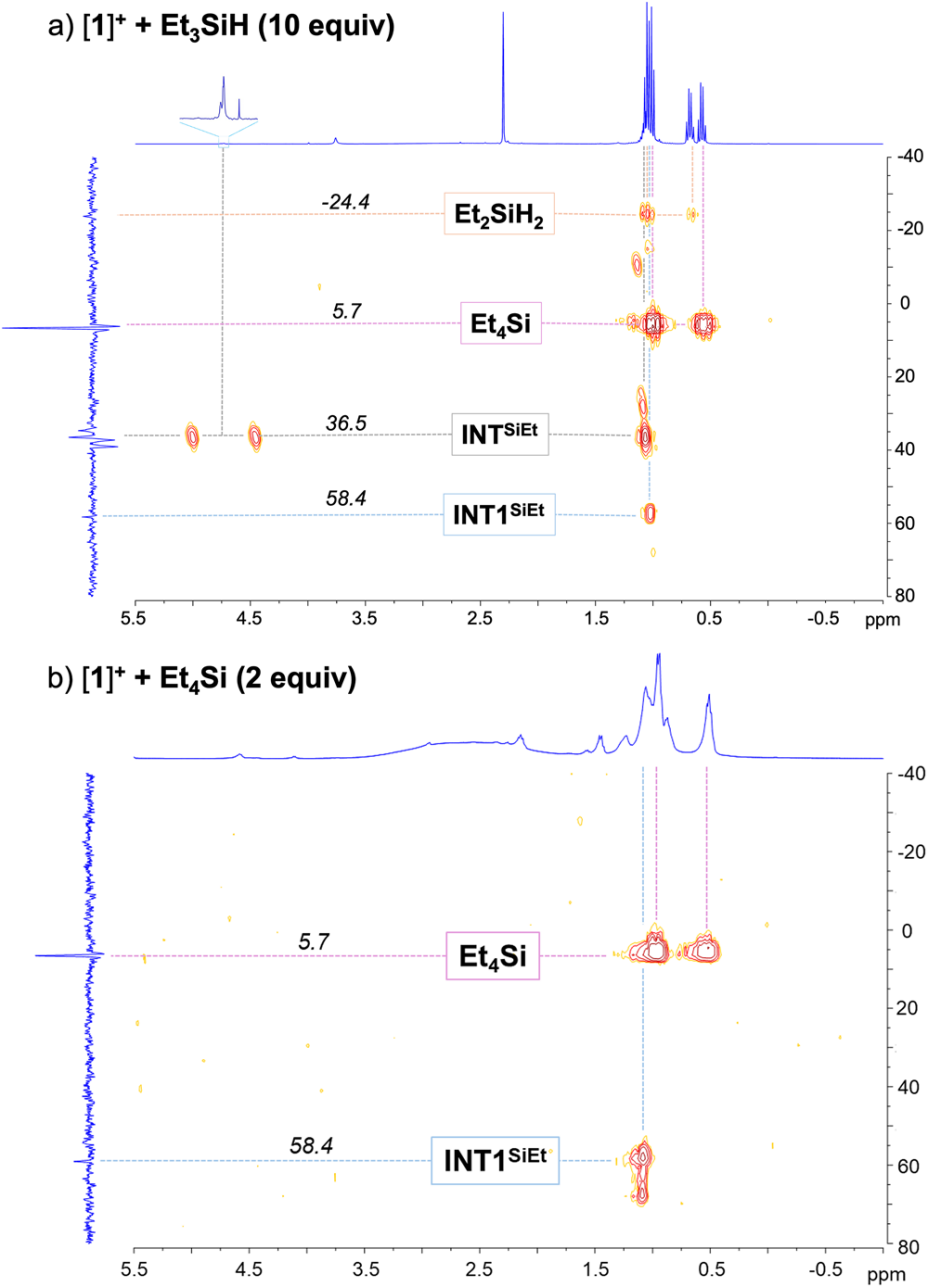
^1^H‐^29^Si HMBC NMR spectra of (a) the reaction between [**1**]^+^[B(C_6_F_5_)_4_]^−^ and Et_3_SiH (10 equiv) in *o*DFB, and (b) the reaction between [**1**]^+^[B(C_6_F_5_)_4_]^−^ and Et_4_Si (2 equiv) in *o*DFB.

Importantly, based on the catalysis shown in Scheme 3, and the DFT‐computed mechanism (Figure 6), together with the silane redistribution products, Et_4_Si and Et_2_SiH_2_, the key intermediates **INT^SiEt^** and **INT1^SiEt^** should also be observable by NMR spectroscopy. To prove the presence of these species, ^1^H‐^29^Si heteronuclear multiple bond correlation (HMBC) NMR spectra of the catalytic Et_3_SiH redistribution reaction (Scheme 3a) were recorded (Figure 7a). In addition to a signal at *δ*(^29^Si) = 5.7 ppm, correlating with ^1^H resonances at *δ* = 0.58 and 1.00 ppm assigned to Et_4_Si [59], and a signal at *δ*(^29^Si) = −24.4 ppm correlating with ^1^H resonances at *δ* = 0.65 and 1.05 ppm assigned to Et_2_SiH_2_ [60], two additional ^29^Si signals were observed. A resonance at *δ*(^29^Si) = 36.5 ppm [61] shows correlations to ^1^H signals at *δ* = 1.07 ppm, attributed to the **Et**‐Si moiety, and at *δ* = 4.45 and 5.00 ppm, attributed to the Si–**H** moiety observed via correlations through ^13^C satellites (Figure 7a; for ^1^H‐^29^Si heteronuclear single‐quantum correlation (HSQC), experiment see Figure S58). On the basis of these correlations, this signal was assigned to the intermediate **INT^SiEt^**. The second signal at *δ*(^29^Si) = 58.4 ppm [61], which correlates with a ^1^H signal at *δ* = 1.02 ppm associated with the **Et**‐Si moiety, was assigned to the second low‐energy intermediate **INT1^SiEt^** (Figure 7a).

To test this assignment, Et_4_Si (2 equiv) was reacted with [**1**]^+^[B(C_6_F_5_)_4_]^−^ in *o*DFB. Based on the proposed mechanism (Scheme 3b and Figure 6), we reasoned that the reverse process, namely OA of the Et_3_Si−Et bond at the cationic Sb center, leading to the formation of **INT1^SiEt^**, should also be feasible. ^1^H‐^29^Si HMBC NMR spectra of this reaction were recorded (Figure 7b). To our delight, in addition to the signal corresponding to Et_4_Si (*δ*(^29^Si) = 5.7 ppm, correlating with ^1^H resonances at *δ* = 0.58 and 1.00 ppm), a second signal, which was assigned to **INT1^SiEt^** in the Et_3_SiH catalytic redistribution (Figure 7a) at *δ*(^29^Si) = 58.4 ppm, correlating with a ^1^H resonance at *δ* = 1.02 ppm, was observed in this case as well (Figure 7b). Remarkably, this experiment further demonstrates that [**1**]^+^ is capable of activating Si−C bonds via OA at the cationic Sb center, even in otherwise inert tetraalkylsilanes.

Taken together with the calculated reaction coordinates (Figure 6), these ^1^H‐^29^Si HMBC (and HSQC) NMR results support the feasibility of the proposed intermediates, **INT^SiEt^** and **INT1^SiEt^**, as well as the metallomimetic nature of the operating catalytic cycle, as shown in Scheme 3b. Notably, similar key mechanistic steps (OA, LM, and RE) have previously been demonstrated in transition‐metal chemistry [62, 63, 64, 65], including in silane redistribution reactions [66, 67, 68].

Since the reaction of Et_4_Si with [**1**]^+^[B(C_6_F_5_)_4_]^−^ described above (Figure 7b), unlike the reaction with Et_3_SiH, does not result in an observable redistribution of substituents, additional evidence for Si−C bond activation was required. To this end, we explored the cross‐redistribution of two different tetraalkylsilanes. Thus, a mixture of Et_4_Si and Me_4_Si was treated with 10 mol% of [**1**]^+^[B(C_6_F_5_)_4_]^−^ in *o*DFB, leading within 30 min to the formation of redistribution products, Me_3_SiEt, Me_2_SiEt_2_, and MeSiEt_3_ (Table 1, entry 1). These results unequivocally demonstrate activation of Si−C bonds in tetraalkylsilanes by [**1**]^+^. We next examined whether [**1**]^+^ can catalyze silane redistribution involving arylsilanes. Accordingly, [**1**]^+^[B(C_6_F_5_)_4_]^−^ (10 mol%) was added to an *o*DFB solution containing Ph_4_Si and Et_4_Si. ^29^Si NMR spectra recorded after 30 min showed complete consumption of the starting silanes and formation of a mixture of redistribution products, Et_3_SiPh, Et_2_SiPh_2_, and EtSiPh_3_ (Table 1, entry 2) [69]. We believe that the mechanism of these redistribution reactions is analogous to that described in Scheme 3b.

**TABLE 1:** Catalytic redistribution of silanes.

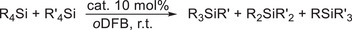				
*Entry*	*Substrates*	*Products*	*t [h]*	*Cat*.
***1***	Me_4_Si Et_4_Si	Me_3_SiEt Me_2_SiEt_2_ MeSiEt_3_	0.5	[**1**]^+^[B(C_6_F_5_)_4_]^−^
***2***	Et_4_Si Ph_4_Si	Et_3_SiPh Et_2_SiPh_2_ EtSiPh_3_	0.5	[**1**]^+^[B(C_6_F_5_)_4_]^−^
3*	Et_4_Si Ph_4_Si	Et_3_SiPh Et_2_SiPh_2_ EtSiPh_3_	24	[Et_3_Si]^+^[B(C_6_F_5_)_4_]^−^

^*^Full consumption of the starting silanes was not achieved after 24 h.

Notably, although transition‐metal‐free silane redistribution has been reported previously, such processes invariably require a hydrosilane to generate a silylium cation that serves as the key active species in catalysis [49, 50, 51, 52, 53, 54, 55, 56, 57, 58]. To the best of our knowledge, catalytic redistribution of tetraorganosilanes proceeding directly, without the involvement of hydrosilanes as a silylium source, has not been reported.

Finally, to assess whether the observed redistribution could instead be catalyzed by an in situ‐generated [Et_3_Si]^+^[B(C_6_F_5_)_4_]^−^, a control experiment was performed using Ph_4_Si and Et_4_Si in the presence of [Et_3_Si]^+^[B(C_6_F_5_)_4_]^−^ (10 mol%) under otherwise identical conditions. In this case, ^29^Si NMR spectroscopy revealed slow formation of the same redistribution products observed in the [**1**]^+^[B(C_6_F_5_)_4_]^−^ ‐catalyzed reaction, however, even after 24 h, complete conversion was not achieved (Table 1, entry 3). These results unambiguously demonstrate that [**1**]^+^[B(C_6_F_5_)_4_]^−^, rather than [Et_3_Si]^+^[B(C_6_F_5_)_4_]^−^ potentially formed in situ, is the active catalyst in this process.

## 3 Conclusion

To conclude, we have synthesized a new cationic, structurally constrained stibenium cation supported by bis(o‐carborano)pyridine, [**1**]^+^[B(C_6_F_5_)_4_]^−^. Reaction of [**1**]^+^[B(C_6_F_5_)_4_]^−^ with a stoichiometric amount of Et_3_SiH led to Si−C bond cleavage, affording **1‐Et**. Combined experimental and DFT mechanistic studies indicate that this activation proceeds via formal oxidative addition of the Si−C bond to the cationic center Sb in [**1**]^+^. The reaction of Et_3_SiH with a catalytic amount of [**1**]^+^[B(C_6_F_5_)_4_]^−^ (10 mol%) at r.t. led to rapid (15 min) silane redistribution. In this Sb^III^/Sb^V^‐based catalysis, [**1**]^+^[B(C_6_F_5_)_4_]^−^ mimics transition‐metal catalysts, following similar key steps (oxidative addition → ligand metathesis → reductive elimination). Remarkably, apolar, less reactive tetraorganosilanes also undergo efficient, catalytic cross‐redistribution. We continue to study the chemistry of the structurally constrained pnictogen centers and their potential in catalysis.

## Author Contributions

**Donia Toami**: investigation, writing – original draft, methodology. **Roman Dobrovetsky**: supervision, conceptualization, investigation, funding acquisition, writing – original draft, writing – review and editing.

## Conflicts of Interest

The authors declare no conflicts of interest.

## Supporting information




**Supporting File 1**: anie72420‐sup‐0001‐SuppMat.pdf.

**Supporting File 2**: anie72420‐sup‐0002‐DataFile.zip.

## Data Availability

The data that supports the findings of this study are available in the supporting information of this article.
