# Supplementary material for: Structurally Constrained Stibenium: Metallomimetic C−Si Bond Activation
Source: Angew Chem Int Ed Engl. 2026 May 1;65(25):e3059944. doi: 10.1002/anie.3059944 (PMC13266915; doi:10.1002/anie.3059944)
Supplement: Supplementary file 1 — Supporting File 1: anie72420‐sup‐0001‐SuppMat.pdf. [file ANIE-65-e3059944-s001.pdf]

Supplementary Information

Structurally Constrained Stibenium: Metallomimetic C−Si Bond Activation

Donia Toami and Roman Dobrovetsky*†

† School of Chemistry, Raymond and Beverly Sackler Faculty of Exact Sciences, Tel Aviv University, Tel Aviv 69978, Israel

Table of Contents

1. General experimental considerations..S3

2. X-ray Crystallography S3

3. Synthetic Procedures S4

4. NMR spectra of the isolated compounds, intermediates and reaction mixtures………………S9

5. DFT calculations…..S46

6. References...S83

1. General experimental considerations

All preparations were carried out under an anhydrous N_2 atmosphere using standard Schlenk and glove box techniques. All glassware was dried and cooled under vacuum before use. Commercial reagents were purchased from Yuanli Tech., Sigma Aldrich, Strem, or Apollo Scientific and used without further purification unless indicated otherwise. **LH₂** and $[Et_3Si][B(C_6F_5)_4]$ were prepared following the reported procedure.[1,2] NMR spectra were recorded at room temperature using a Bruker Avance III-400 MHz and 500MHz spectrometer. Data for ^1H NMR are reported as follows: chemical shift (δ ppm), integration, multiplicity (s = singlet, d = doublet, t = triplet, q = quartet, quin = quintet, sep = septet, m = multiplet, br = broad), coupling constant (Hz), assignment. High-resolution mass spectrometry was performed with a Waters SYNAPT system. GC-MS analyses were obtained on a 7890B GC system equipped with a DB-5ms UI column (30 m × 0.25 mm ID × 0.25 μm film) and 5977A mass selective detector (Agilent Technologies).

2. X-ray Crystallography

The crystals for all the reported compounds herein were mounted on a cryoloop with Paratone oil, and all data were collected at 110(2) K. Single crystal X-ray diffraction data for **1-Cl** was collected on a Bruker KAPPA APEXDuo diffractometer equipped with an APEX II CCD detector using a TRIUMPH monochromator with a Mo*Kα* X-ray source (*α* = 0.71073 Å). Unit cell determination, refinement and data collection were done using the Bruker APEX-III suite[3] data reduction and integration were performed using SAINT v8.34A (Bruker)[4] and absorption corrections and scaling were done using SADABS-2014/5 (Bruker)[5]. The crystal structure of **1-Cl** was solved through the OLEX2[6] package using SHELXT[7] and the structures were refined using SHELXL[8]. All non-hydrogen atoms were refined anisotropically. Single crystal X-ray diffraction data for **1-H** and **1-Et** were collected on a Bruker D8 VENTURE FIXED CHI diffractometer with a microfocus sealed tube using a multilayer mirror as monochromator and a Bruker PHOTON III CPAD detector. The diffractometer was equipped with an Oxford Cryostream 1000 low temperature device and used MoK_α radiation (λ = 0.71073 Å). All data were integrated with SAINT V8.40B[9]. Multi-Scan absorption correction using SADABS 2016/2 was applied.[10] The structure was solved by Intrinsic Phasing methods with SHELXT 2018/2 and refined by full-matrix least-squares methods against F^2 using SHELXL-2019/2.[7,8] All non-hydrogen atoms were refined with anisotropic displacement parameters. CCDC numbers for the compounds **1-Cl**, **1-H** and **1-Et** are 2501485, 2501486 and 2501487, respectively.

3. Synthetic Procedures

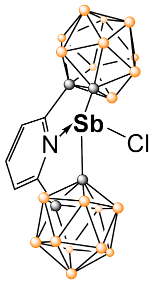

3.1 Synthesis of 1-Cl: ^{n}BuLi (6.2 mL, 9.90 mmol, 1.6 M in hexane) was added to a Et_2O solution (50 mL) of **LH_2** (1.5 g, 4.13 mmol) at 0 °C. The reaction mixture was allowed to warm to room temperature. The formation of a light brown oil could be observed as a separate layer at the bottom of the flask upon standing. The solution was stirred for an additional 48 h at room temperature. Et_2O was evaporated under reduced pressure to give a sticky solid, which was dissolved in 50 mL of benzene. A solution of $SbCl_3$ (0.94 g, 4.13 mmol) dissolved in benzene (7 mL) was added to the reaction mixture at −30 °C. An immediate change to a yellow-colored solution was observed after the addition. The reaction mixture was allowed to warm slowly to room temperature and stirred for 18 h. All the volatiles were removed under vacuum, and the solid was extracted with CH_2Cl_2 (50 mL). A light-yellow precipitate was obtained during the concentration of the solution. The obtained solid was crystallized by slow evaporation from CH_2Cl_2/hexane mixture (4:1), affording yellow crystals of **1-Cl**. Isolated yield: 1.2 g (56%).

^{1}H NMR (400 MHz, $CDCl_3$); δ = 8.11 (t, 1H, J = 7.8 Hz, *p*-Ar-*H*), 7.66 (d, 2H, J = 7.6 Hz, *m*-Ar-*H*), 2.36 (br, 20H, B-*H*) ppm. ^{13}C NMR (100 MHz, $CDCl_3$); δ = 149.84, 144.12, 118.11, 77.86, 72.85 ppm. ^{11}B NMR (128 MHz, $CDCl_3$); δ = -2.01 (d, J = 147.2 Hz), -5.83 (t, J = 160.0 Hz), -9.26 (m), -12.55 (m) ppm. HRMS (APCI$^+$): Calculated for $C_9H_{23}{}^{10}B_8{}^{11}B_{12}N^{35}Cl^{121}Sb$: 513.2709 [M]$^+$; Obs: 513.2704.

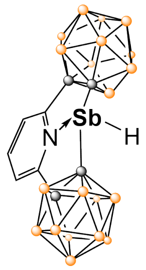

3.2 Synthesis of 1-H: DIBAL-H (0.48 mL, 0.58 mmol, 1.2 M in toluene) was added to a solution of **1-Cl** (300 mg, 0.58 mmol) dissolved in 10 mL toluene. The reaction mixture was stirred for 18 h at room temperature. All the volatiles were removed under reduced pressure, and the obtained solid was washed with 2 x 10 mL hexane to afford a brown solid of **1-H**. **1-H** was crystallized by slow evaporation from benzene. Isolated yield: 182 mg (65%).

^{1}H NMR (400 MHz, $CDCl_3$); δ = 11.24 (s, 1H), 8.01 (t, 1H, J = 7.8 Hz, *p*-Ar-*H*), 7.65 (d, 2H, J = 8 Hz, *m*-Ar-*H*), 2.52 (br, 20H, B-*H*) ppm. ^{13}C NMR (100 MHz, $CDCl_3$); δ =148.96, 142.68, 122.26, 71.34, 66.65 ppm. ^{11}B NMR (128 MHz, $CDCl_3$); δ = -3.07 (d, J = 150.91 Hz), -5.13 (d, J = 152.06 Hz), -9.09 (m), -11.93 (m) ppm. HRMS (APPI$^+$): Calculated for $C_9H_{23}{}^{10}B_3{}^{11}B_{17}N^{121}Sb$: 483.2839 [M-H$^-$]$^+$; Obs: 483.2847.

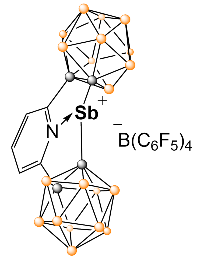

3.3 Synthesis of [1]$^+$[B(C_6F_5)$_4$]$^-$: [Et_3Si]$^+$[B(C_6F_5)$_4$]$^-$ was prepared by the addition of Et_3SiH (185.3 µL, 1.16 mmol) to a 10 mL toluene solution containing [Ph_3C]$^+$[B(C_6F_5)$_4$]$^-$ (533 mg, 0.58 mmol). The reaction was allowed to stir for 20 minutes to afford light brown oil. The oil obtained was further washed with 2 x 10 mL toluene. A white solid was obtained upon washing this oil with 15 mL of hexane. A toluene solution (10 mL) of **1-Cl** (300 mg, 0.58 mmol) was added to the obtained solid. The mixture was stirred for 18 h at room temperature, producing brown oil. The oil was separated and washed with 2 x 10 mL hexane to afford a yellow solid of [**1**]$^+$[B(C_6F_5)$_4$]$^-$. Yield: 230 mg (34%).

^{1}H NMR (400 MHz, *o*DFB, DMSO-D_6 capillary); *δ* = 8.18 (t, 1H, *J* = 7.6 Hz, *p*-Ar-*H*), 7.72 (d, 2H, *J* = 7.6 Hz, *m*-Ar-*H*), 2.67 (br, 20H, B-*H*) ppm. ^{13}C NMR (100 MHz, *o*DFB, DMSO-D_6 capillary); *δ* = 150.20, 147.89 (d, $J_{C\text{-}F}$ = 240 Hz, ArC-F), 146.19, 137.73 (d, $J_{C\text{-}F}$ = 245 Hz, ArC-F), 135.87 (d, $J_{C\text{-}F}$ = 242 Hz, ArC-F), 123.10, 79.12, 75.84 ppm. ^{11}B NMR (128 MHz, *o*DFB, DMSO-D_6 capillary); *δ* = -1.26 (m), -8.31 (m), -16.86 (s) ppm. ^{19}F NMR (376.5 MHz, *o*DFB, DMSO-D_6 capillary); *δ* = -133.03, -164.20, -168.03 ppm. HRMS (APPI$^+$): Calculated for $C_9H_{23}{}^{10}B_8{}^{11}B_{12}N^{121}Sb$: 478.3020 [M]$^+$; Obs: 478.3011.

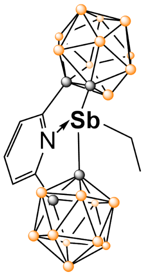

3.4 Synthesis of 1-Et: Et_3Si-H (6.9 µL, 0.04 mmol) was added to a solution of [**1**]$^+$[B(C_6F_5)$_4$]$^-$ (50 mg, 0.04 mmol) dissolved in 1,2-difluorobenzene (*o*DFB). After stirring for 15 minutes at room temperature, full consumption of the starting materials was observed by multinuclear NMR, along with **1-Et** formation. Slow evaporation of the solvent afforded yellow crystals of **1-Et**. Yield: 12 mg (55%).

^{1}H NMR (400 MHz, $CDCl_3$); *δ* = 7.96 (t, 1H, *J* = 7.8 Hz, *p*-Ar-*H*), 7.62 (d, 2H, *J* = 7.6 Hz, *m*-Ar-*H*), 2.18 (q, 2H, *J* = 7.8 Hz, -***CH₂***-CH₃), 1.48 (t, 3H, *J* = 8 Hz, -CH₂-***CH₃***), 2.44 (br, 20H, B-*H*) ppm. ^{13}C NMR (100 MHz, $CDCl_3$); *δ* = 148.44, 141.52, 121.50, 71.97, 69.44, 25.70, 10.64 ppm. ^{11}B NMR (128 MHz, $CDCl_3$); *δ* = -2.46 to -11.58 ppm (*o*Cb).

3.5 Redistribution of triethyl silane: triethyl silane (50 µL, 0.3 mmol) was added to a J-Young NMR tube charged with 0.5 mL *o*DFB solution of [**1**]$^+$[B(C_6F_5)$_4$]$^-$ (36 mg, 0.03mmol). The reaction mixture afforded after 15 minutes at RT a full conversion into the redistributed silane products: di- and tetra-ethyl silane. The formation of these products was confirmed by multinuclear NMR of the reaction mixture.

diethyl silane: ^{1}H NMR (400 MHz, *o*DFB, DMSO-D_6 capillary); δ = 0.63 (q, 4H, *J* = 7.8 Hz), 1.01 (t, 6H, *J* = 8 Hz), 3.71 (s, 2H) ppm. ^{13}C NMR (100 MHz, *o*DFB, DMSO-D_6 capillary); *δ* = 0.48, 8.00 ppm. ^{29}Si NMR (79.5 MHz, *o*DFB, DMSO-D_6 capillary); δ = -24.47 ppm. **tetraethyl silane:** ^{1}H NMR (400 MHz, *o*DFB, DMSO-D_6 capillary); δ = 0.52 (q, 8H, *J* = 8 Hz), 0.96 (t, 12H, *J* = 7.8 Hz) ppm. ^{13}C NMR (100 MHz, *o*DFB, DMSO-D_6 capillary); *δ* = 2.47, 6.51 ppm. ^{29}Si NMR (79.5 MHz, *o*DFB, DMSO-D_6 capillary); δ = 6.64 ppm. ^{11}B NMR (128 MHz, *o*DFB, DMSO-D_6 capillary); δ = -16.29 (s, $^-$B(C_6F_5)$_4$), -2.72 to -12.02 (*o*Cb) ppm. ^{19}F NMR (376.5 MHz, *o*DFB, DMSO-D_6 capillary); δ = -167.70, -163.92, -132.55 ppm.

3.6 Redistribution of dimethylphenyl silane: dimethylphenyl silane (26 µL, 0.17 mmol) was added to a J-Young NMR tube charged with 0.5 mL *o*DFB solution of [**1**]$^+$[B(C_6F_5)$_4$]$^-$ (20 mg, 0.017 mmol). The reaction mixture afforded after 30 minutes at RT a full conversion into the redistributed silane products: tetramethyl silane, trimethylphenyl silane, dimethyldiphenyl silane, and methyltriphenyl silane. The formation of these products was confirmed by multinuclear NMR of the reaction mixture and GC-MS spectrometry.

^{1}H NMR (400 MHz, *o*DFB, DMSO-D_6 capillary); δ = 0.08 (s, 12H, (CH_3)$_4$Si), 0.34 (s, 9H, (CH_3)$_3$SiPh), 0.60 (s, 6H, (CH_3)$_2$SiPh_2), 0.85 (s, 3H, CH_3SiPh_3) ppm. All the aromatic hydrogens of the three products are merged at δ = 7.36, 7.58 ppm. ^{13}C NMR (100 MHz, *o*DFB, DMSO-D_6 capillary); *δ* = -1.78 (CH_3)$_4$Si, -2.91, -4.17, -5.07 (the -CH_3 signals of the remaining three products), 126.96 to 128.94 (meta and para positions of the aromatic carbons of the three products), 132.40 to 135.58 (ortho positions of the aromatic carbons of the three products) ppm. ^{29}Si NMR (79.5 MHz, *o*DFB, DMSO-D_6 capillary); δ = -1.13 ((CH_3)$_4$Si), -5.33 ((CH_3)$_3$SiPh), -9.31 ((CH_3)$_2$SiPh_2), -12.31 (CH_3SiPh_3) ppm. ^{11}B NMR (128 MHz, *o*DFB, DMSO-D_6 capillary); δ = -16.19 (s, $^-$B(C_6F_5)$_4$), -2.62 to -12.19 (*o*Cb) ppm. ^{19}F NMR (376.5 MHz, *o*DFB, DMSO-D_6 capillary); δ = -167.46, -163.66, -132.36 ppm.

3.7 Redistribution of tetramethyl silane and tetraethyl silane: tetramethyl silane (35 µL, 0.26 mmol) and tetraethyl silane (32 µL, 0.17 mmol) were added to a J-Young NMR tube charged with 0.5 mL *o*DFB solution of $[\mathbf{1}]^+[B(C_6F_5)_4]^-$ (20 mg, 0.017 mmol). The reaction mixture afforded after 30 minutes at RT a full conversion into the redistributed silane products: ethyltrimethyl silane, diethyldimethyl silane, and triethylmethyl silane. The formation of these products was confirmed by multinuclear NMR of the reaction mixture and GC-MS spectrometry.

^{1}H NMR (400 MHz, *o*DFB, DMSO-D_6 capillary); = -0.06 (s, $(CH_3)_4Si$ – residuals due to the excess), -0.03, 0.01, 0.04 (s, Si-CH_3 groups of the three products, 18H), merged signals of the three products at δ = 0.49 (q, Si-CH_2CH$_3$, 12H, *J* = 7.6 Hz), 0.96 (t, Si-CH$_2$CH_3, 18H, *J* = 7.8 Hz) ppm. ^{13}C NMR (100 MHz, *o*DFB, DMSO-D_6 capillary); ethyltrimethyl silane: *δ* = -3.30, 6.05, 6.50 ppm; diethyldimethyl silane: *δ* = -5.51, 7.89, 6.50 ppm; triethylmethyl silane: *δ* = -7.73, 4.25, 6.50 ppm. ^{29}Si NMR (79.5 MHz, *o*DFB, DMSO-D_6 capillary); ethyltrimethyl silane: *δ* = 1.90 ppm. diethyldimethyl silane: *δ* = 4.24 ppm. triethylmethyl silane: *δ* = 6.14 ppm. ^{11}B NMR (128 MHz, *o*DFB, DMSO-D_6 capillary); δ = -16.90 (s, $^-B(C_6F_5)_4$), -3.18 to -12.80 (*o*Cb) ppm. ^{19}F NMR (376.5 MHz, *o*DFB, DMSO-D_6 capillary); δ = -167.68, -163.80, -132.65 ppm.

3.8 Redistribution of tetramethyl silane and tetraphenyl silane: tetramethyl silane (47 µL, 0.34 mmol) and tetraphenyl silane (57 mg, 0.17 mmol) were added to a J-Young NMR tube charged with 0.5 mL DFB solution of $[\mathbf{1}]^+[B(C_6F_5)_4]^-$ (20 mg, 0.017 mmol). The reaction mixture afforded after 30 minutes at RT a full conversion into the redistributed silane products: trimethylphenyl, dimethyldiphenyl silane, and methyltriphenyl silane. The formation of these products was confirmed by multinuclear NMR of the reaction mixture and GC-MS spectrometry.

^{1}H NMR (400 MHz, *o*DFB, DMSO-D_6 capillary); 0.06 (s, $(CH_3)_4Si$ – residuals due to the excess), 0.30 (s, 9H, $((CH_3)_3SiPh)$), 0.56 (s, 6H, $((CH_3)_2SiPh_2)$), 0.70 (s, 3H, (CH_3SiPh_3)), All the aromatic hydrogens of the three products are merged at δ = 7.34, 7.56 ppm. ^{13}C NMR (100 MHz, *o*DFB, DMSO-D_6 capillary); *δ* = -1.58 ($(CH_3)_4Si$ – due to excess), -2.68, -3.90, -4.88 (-CH_3 signals of all the products), 127.07 to 128.66 (meta and para positions of the aromatic carbons of all the products), 132.58, 133.46, 134.57 (ortho position of the aromatic carbons of all the products) ppm. ^{29}Si NMR (79.5 MHz, *o*DFB, DMSO-D_6 capillary); $(CH_3)_4Si$: *δ* = -0.73 ppm, $((CH_3)_3SiPh)$**:** *δ* = -4.92 ppm, $((CH_3)_2SiPh_2)$**:** *δ* = -8.92 ppm, (CH_3SiPh_3): *δ* = -11.88 ppm. ^{11}B NMR (128 MHz, *o*DFB, DMSO-D_6 capillary); δ = -16.76 (s, $^-B(C_6F_5)_4$), -3.02 to -12.10 (*o*Cb) ppm. ^{19}F NMR (376.5 MHz, *o*DFB, DMSO-D_6 capillary); δ = -168.06, -164.28, -132.95 ppm.

3.9 Redistribution of tetraethyl silane and tetraphenyl silane: tetraethyl silane (32 µL, 0.17 mmol) and tetraphenyl silane (58 mg, 0.17 mmol) were added to a J-Young NMR tube charged with 0.5 mL *o*DFB solution of [**1**]$^+$[B(C_6F_5)$_4$]$^-$ (20 mg, 0.017 mmol). The reaction mixture afforded after 30 minutes at RT a full conversion into the redistributed silane products: triethylphenyl silane, diethyldiphenyl silane, and ethyltriphenyl silane. The formation of these products was confirmed by multinuclear NMR of the reaction mixture and GC-MS spectrometry.

^{1}H NMR (400 MHz, *o*DFB, DMSO-D$_6$ capillary); Ethyl signals of triethylphenyl silane: δ = 0.90 (q), 1.05 to 1.11 (m) ppm, diethyldiphenyl silane**:** δ = 1.05 to 1.17 ppm, ethyltriphenyl silane: δ = 1.17 to 1.24, 1.45 to 1.57 ppm. All the aromatic hydrogens of the three products are merged at δ = 7.43, 7.70 ppm. ^{13}C NMR (100 MHz, *o*DFB, DMSO-D$_6$ capillary); δ = 2.50 to 8.45 (ethyl signals of the three products), 127.19 to 128.96 (meta and para positions of the aromatic carbons of the three products), 134.25, 135.00, 135.79 (ortho position of the aromatic carbons of the three products) ppm. ^{29}Si NMR (79.5 MHz, *o*DFB, DMSO-D$_6$ capillary); triethylphenyl silane**:** δ = 0.44 ppm, diethyldiphenyl silane**:** δ = -5.42 ppm, ethyltriphenyl silane: δ = -10.85 ppm. ^{11}B NMR (128 MHz, *o*DFB, DMSO-D$_6$ capillary); δ = -16.61 (s, $^-$B(C_6F_5)$_4$), -2.69 to -12.11 (*o*Cb) ppm. ^{19}F NMR (376.5 MHz, *o*DFB, DMSO-D$_6$ capillary); δ = -167.98, -164.14, -132.89 ppm.

4. NMR spectra of the isolated compounds, intermediates and reaction mixtures

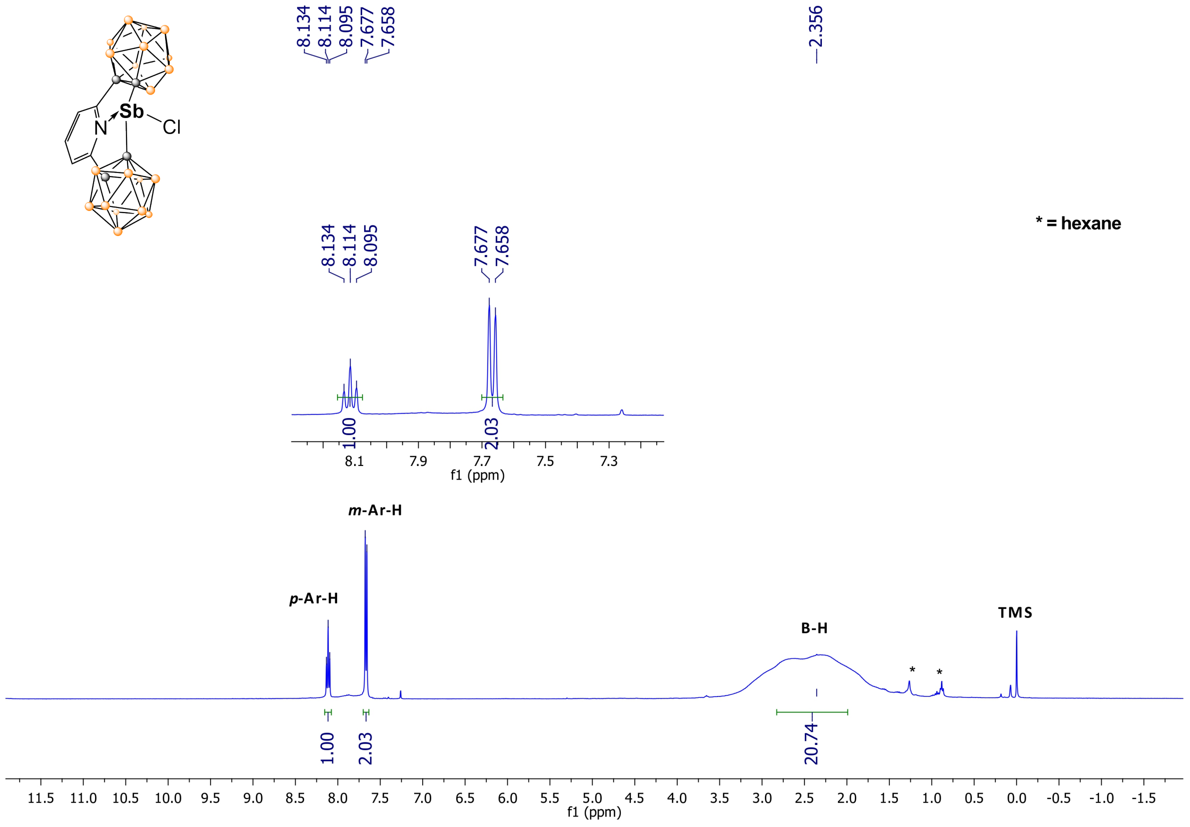

Figure S1. ^{1}H NMR spectrum (400 MHz, $CDCl_3$) of **1-Cl**.

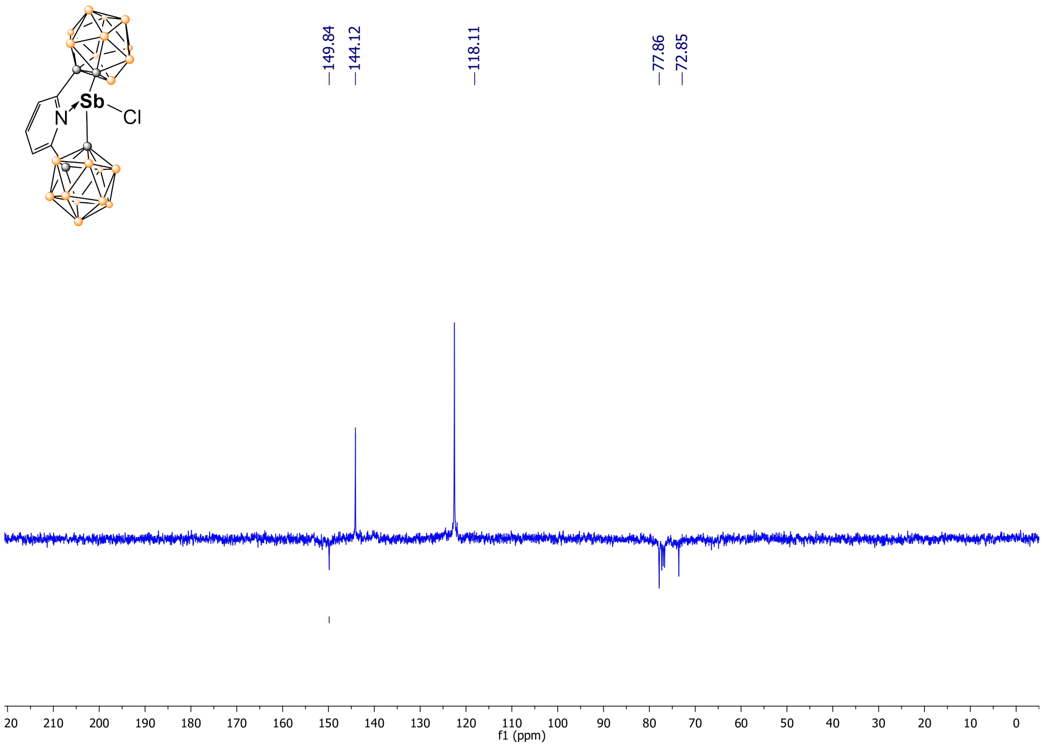

Figure S2. ^{13}C-JMOD NMR spectrum (100 MHz, $CDCl_3$) of **1-Cl**.

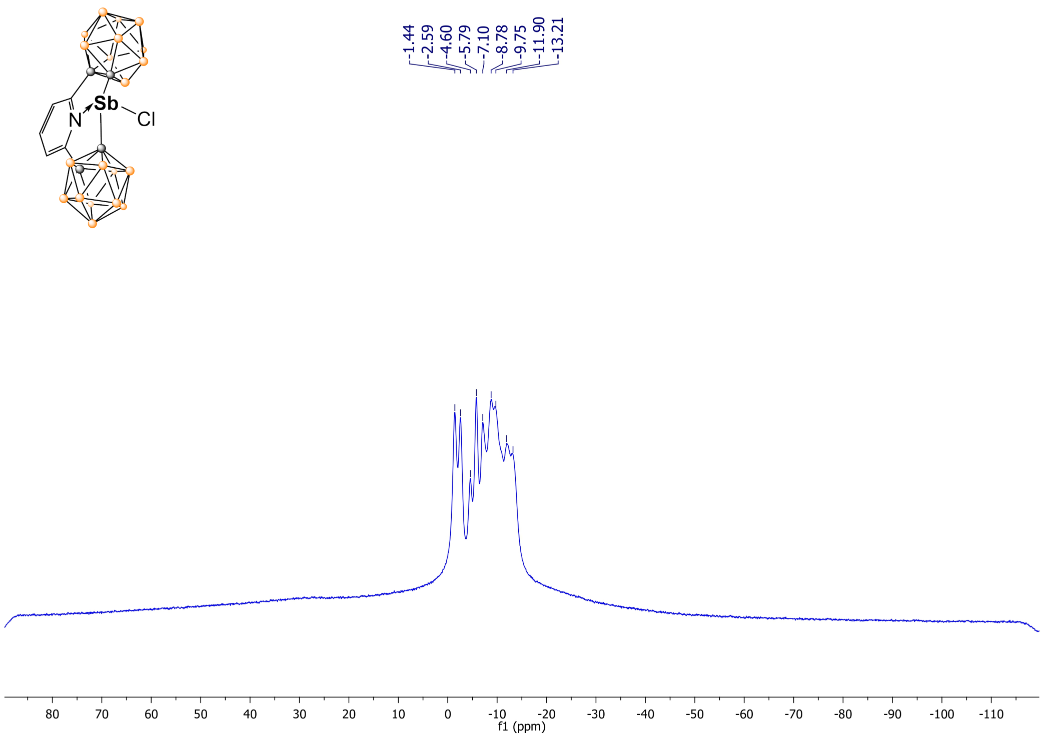

Figure S3. ^{11}B NMR spectrum (128 MHz, $CDCl_3$) of **1-Cl**.

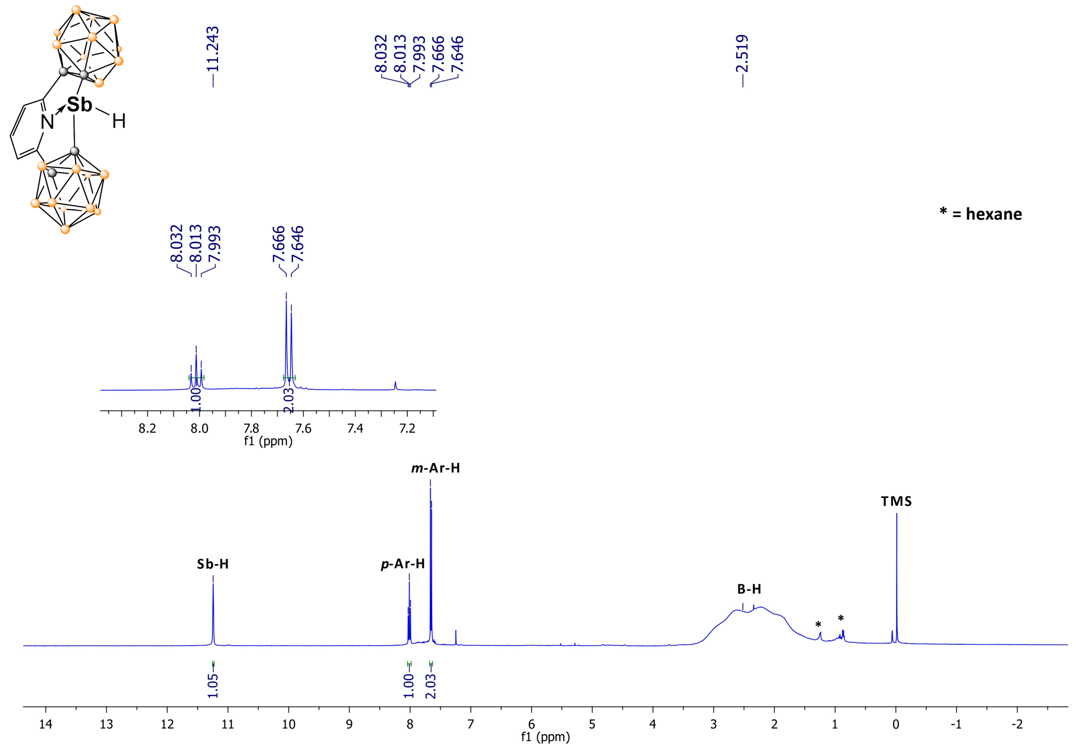

Figure S4. ^{1}H NMR spectrum (400 MHz, $CDCl_3$) of **1-H**.

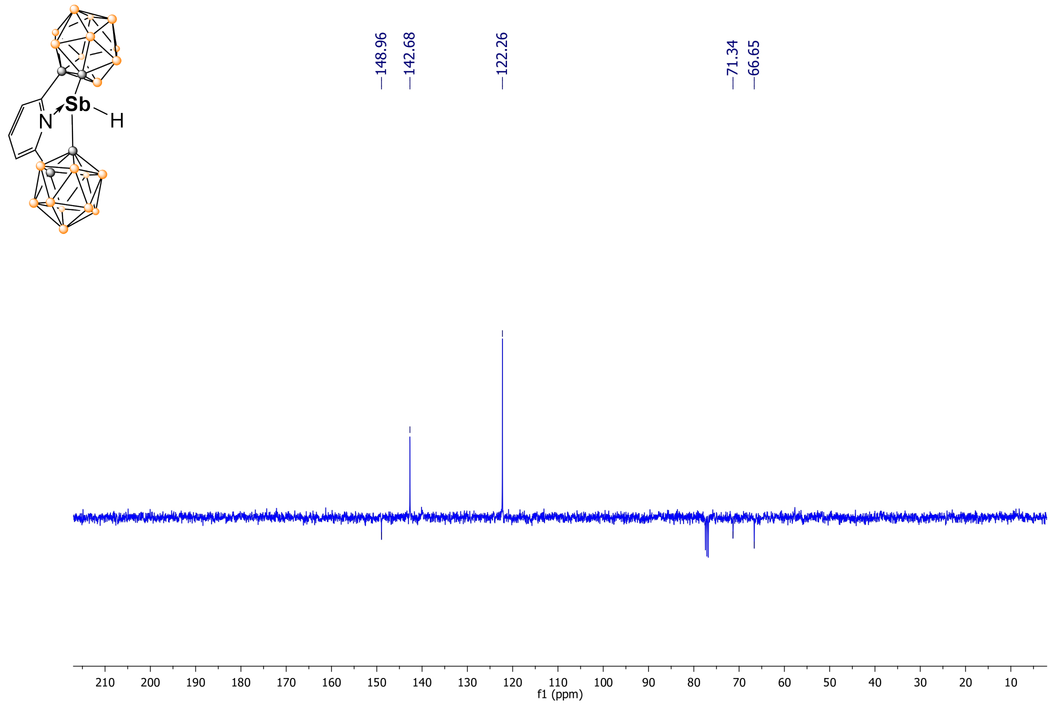

Figure S5. ^{13}C-JMOD NMR spectrum (100 MHz, $CDCl_3$) of **1-H**.

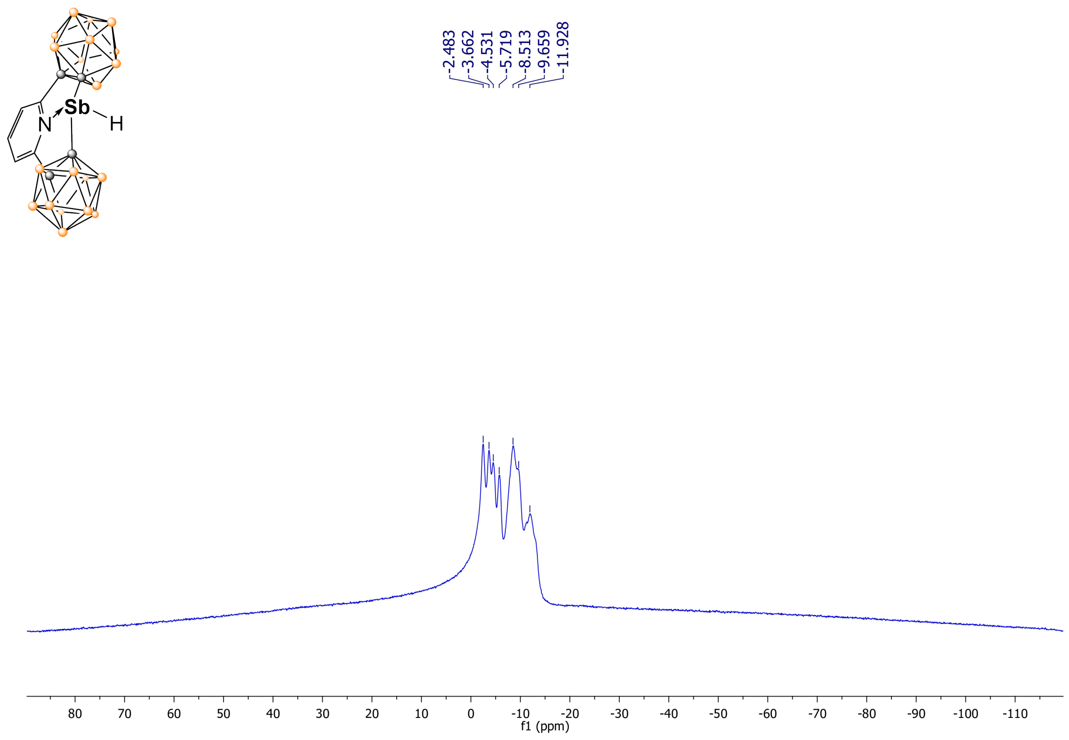

Figure S6. ^{11}B NMR spectrum (128 MHz, $CDCl_3$) of **1-H**.

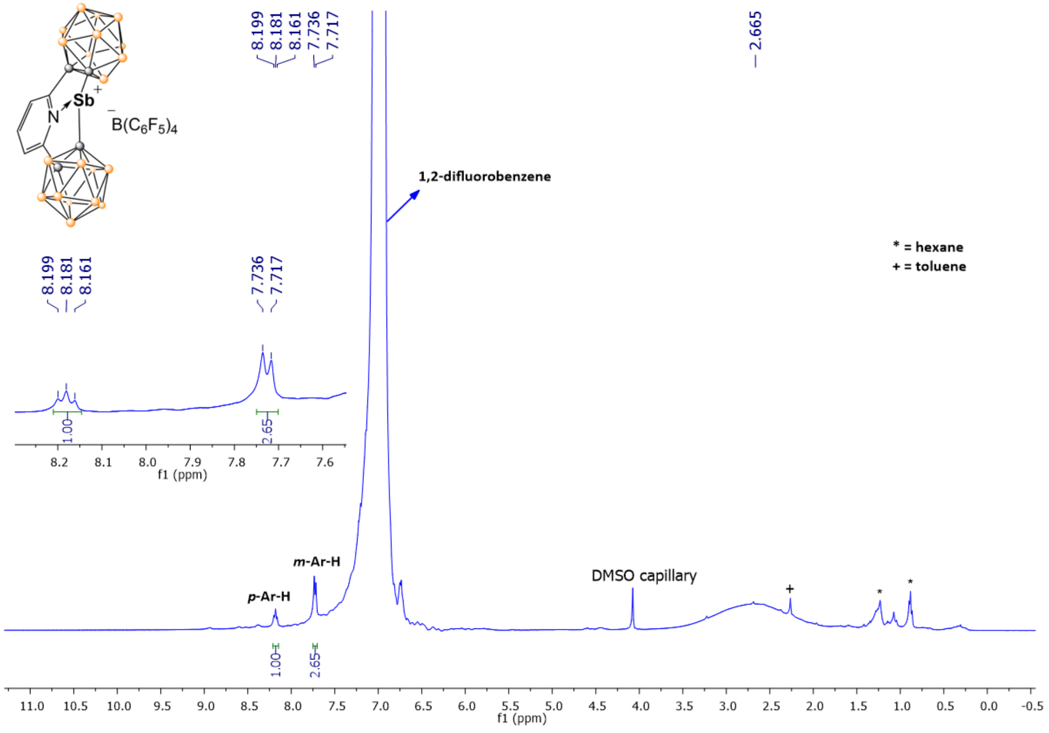

Figure S7. ^{1}H NMR spectrum (400 MHz, *o*DFB, DMSO-D$_6$ capillary) of $[\mathbf{1}]^+[B(C_6F_5)_4]^-$.

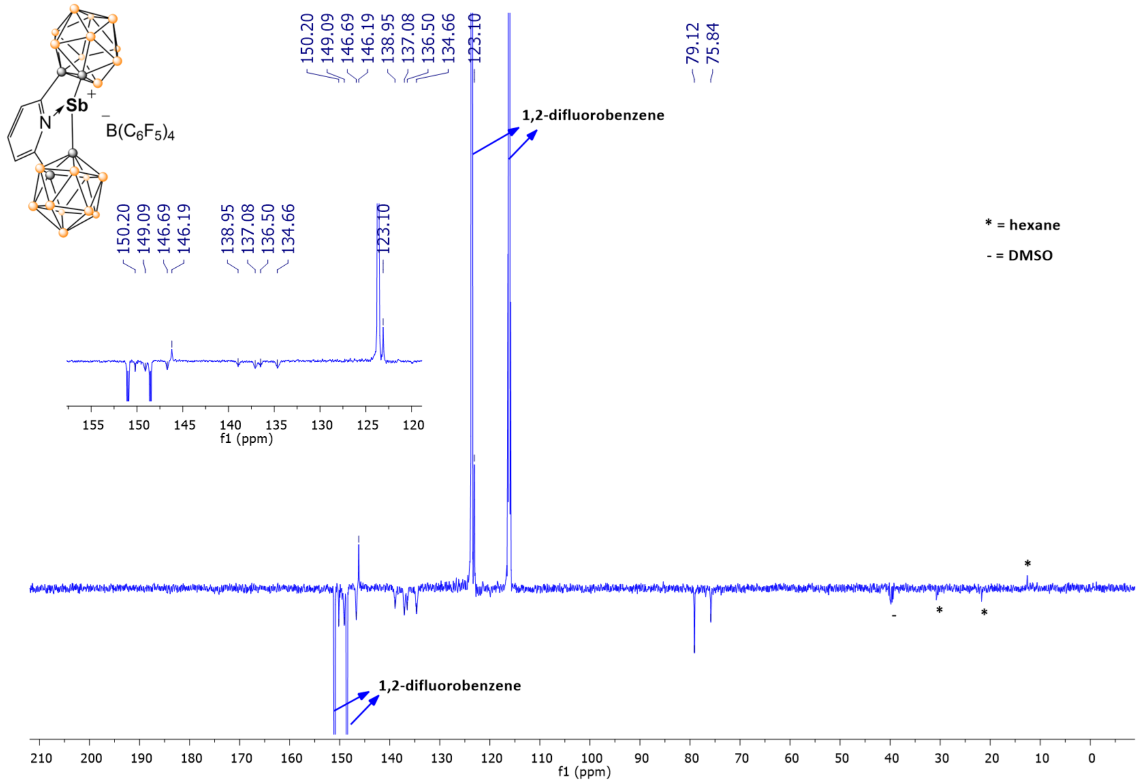

Figure S8. ^{13}C-JMOD NMR spectrum (100 MHz, *o*DFB, DMSO-D$_6$ capillary) of [**1**]$^+$[B(C$_6$F$_5$)$_4$]$^-$.

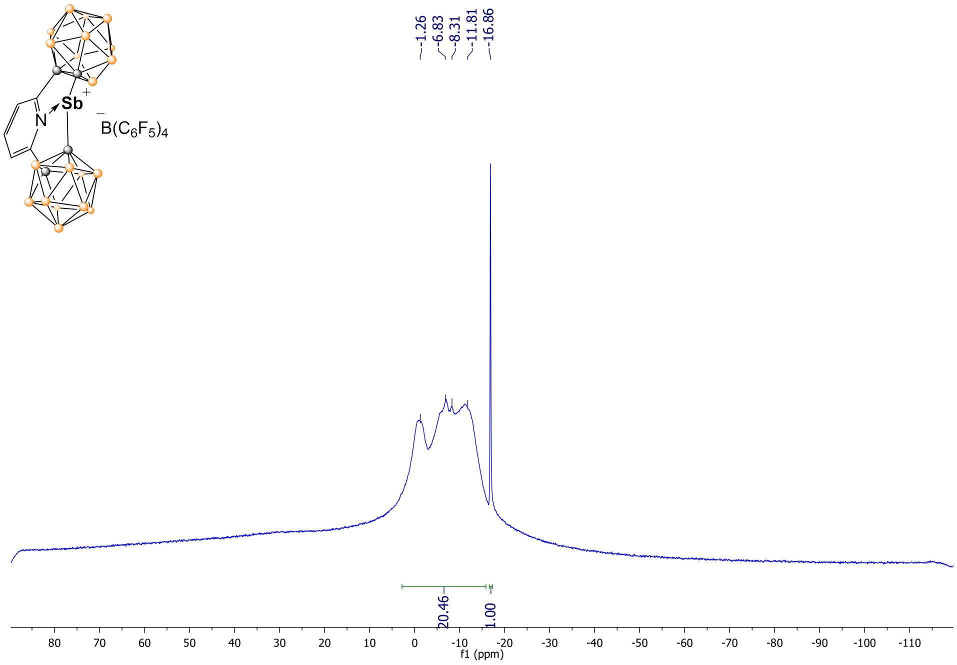

Figure S9. ^{11}B NMR spectrum (128 MHz, *o*DFB, DMSO-D$_6$ capillary) of [**1**]$^+$[B(C$_6$F$_5$)$_4$]$^-$.

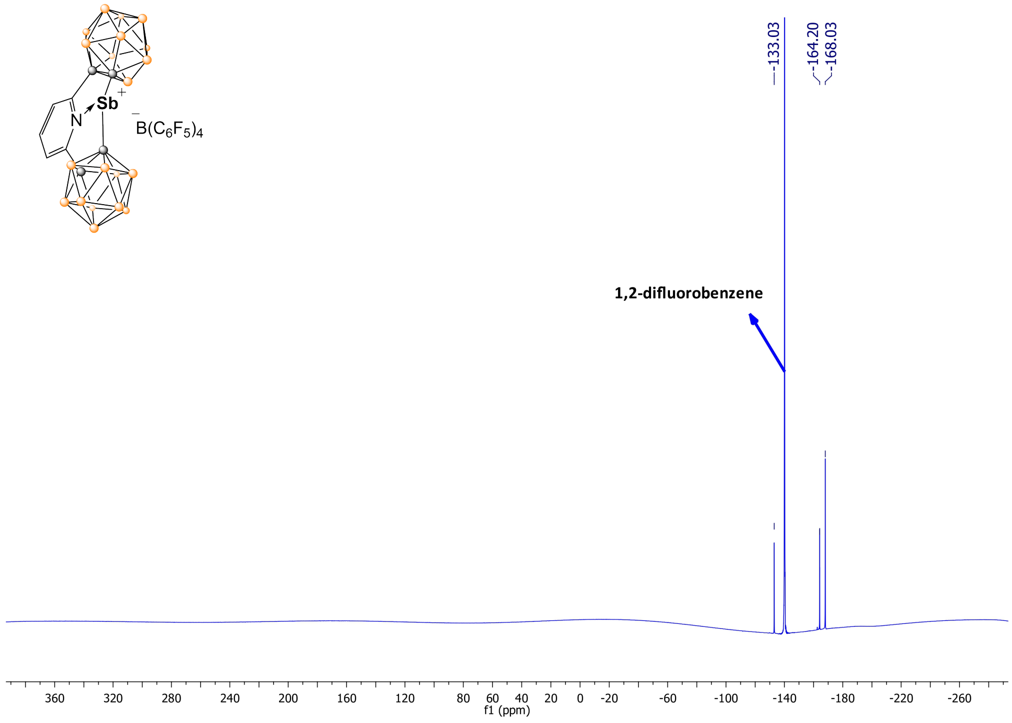

Figure S10. ^{19}F NMR spectrum (376.5 MHz, *o*DFB, DMSO-D$_6$ capillary) of $[\mathbf{1}]^+[B(C_6F_5)_4]^-$.

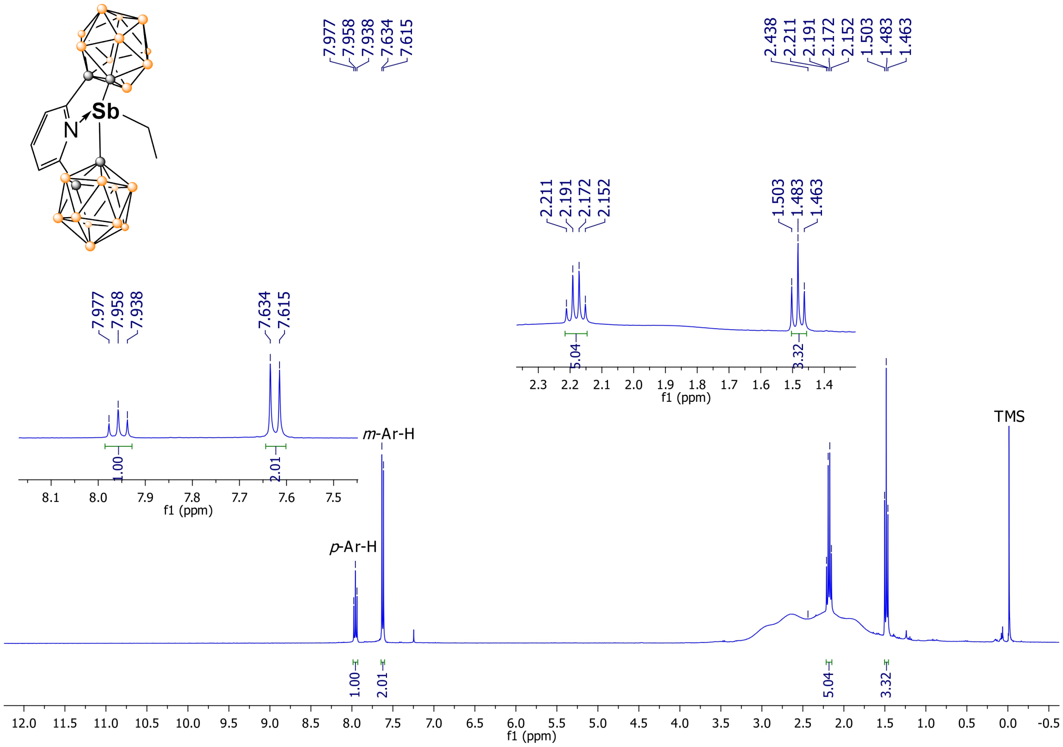

Figure S11. ^{1}H NMR spectrum (400 MHz, $CDCl_3$) of **1-Et.**

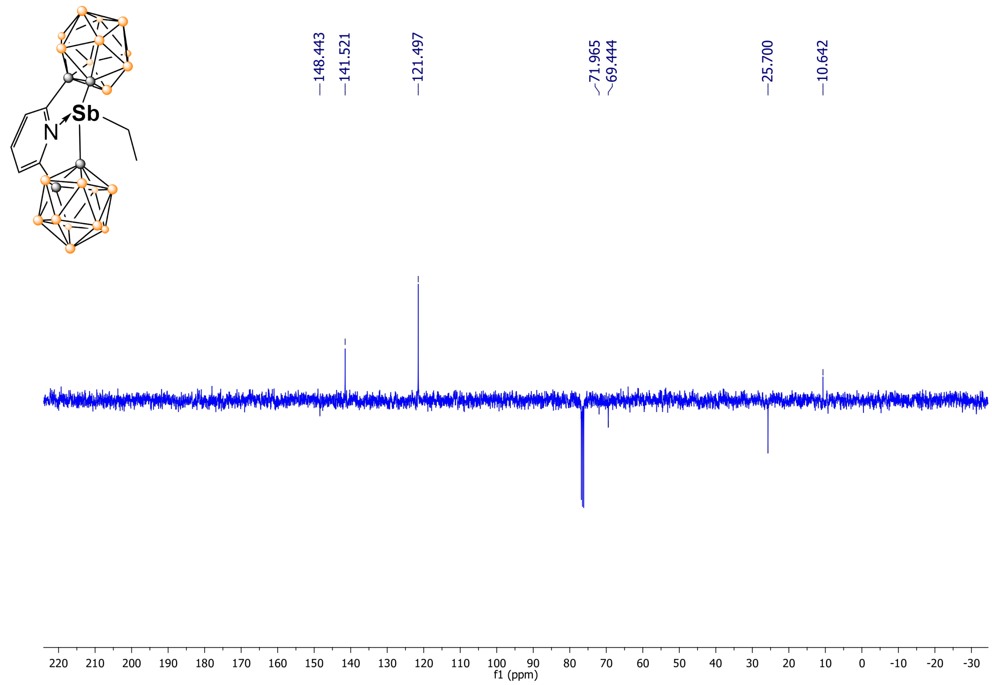

Figure S12. ^{13}C-JMOD NMR spectrum (100 MHz, $CDCl_3$) of **1-Et**.

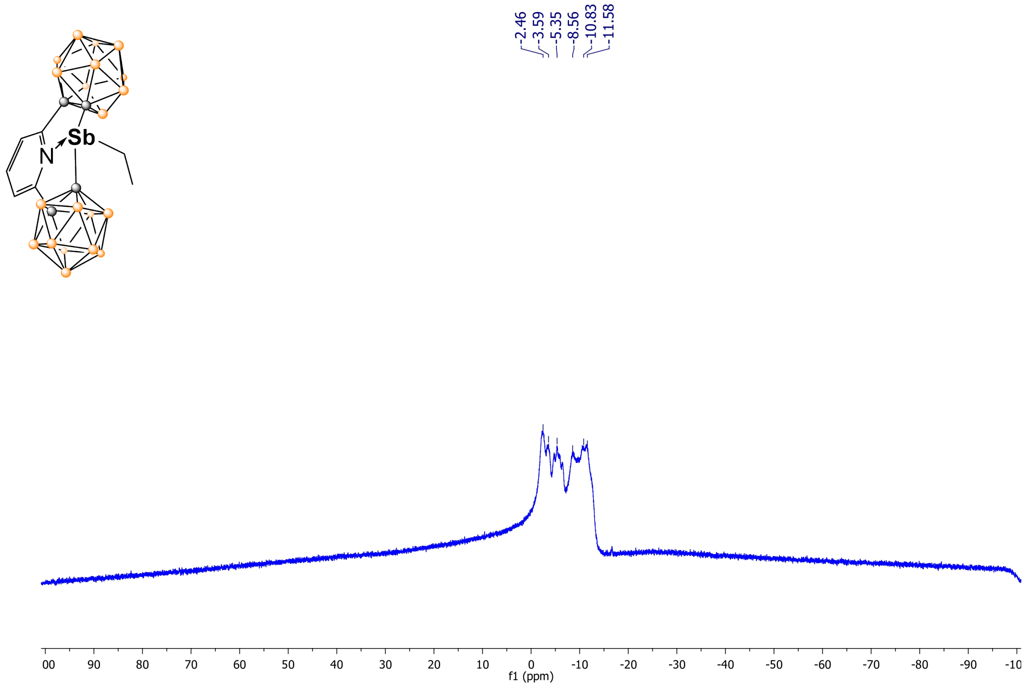

Figure S13. ^{11}B NMR spectrum (128 MHz, $CDCl_3$) of **1-Et**.

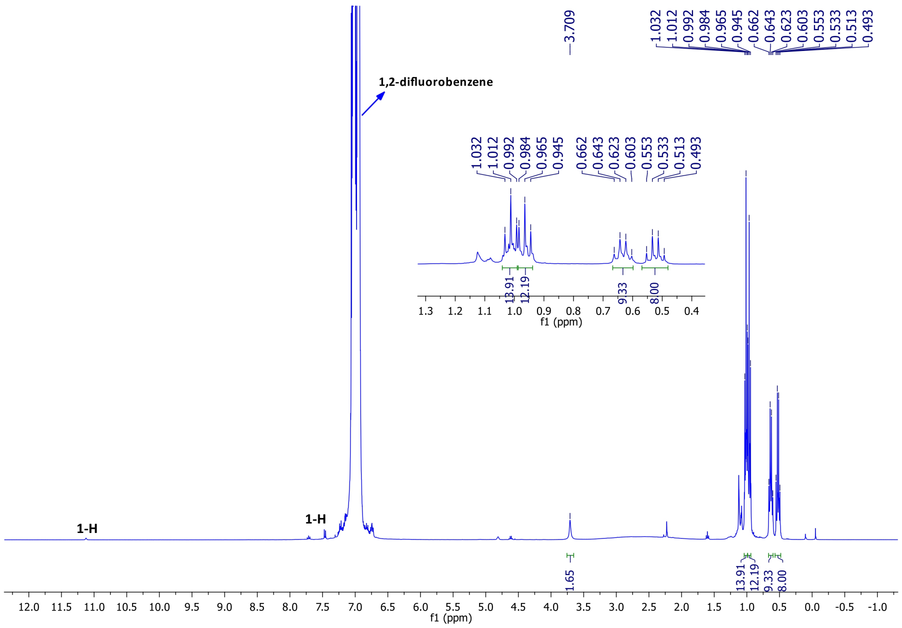

Figure S14. ^{1}H NMR spectrum (400 MHz, *o*DFB, DMSO-D$_6$ capillary) of the reaction mixture between [**1**]$^+$[B(C_6F_5)$_4$]$^-$ and Et_3SiH.

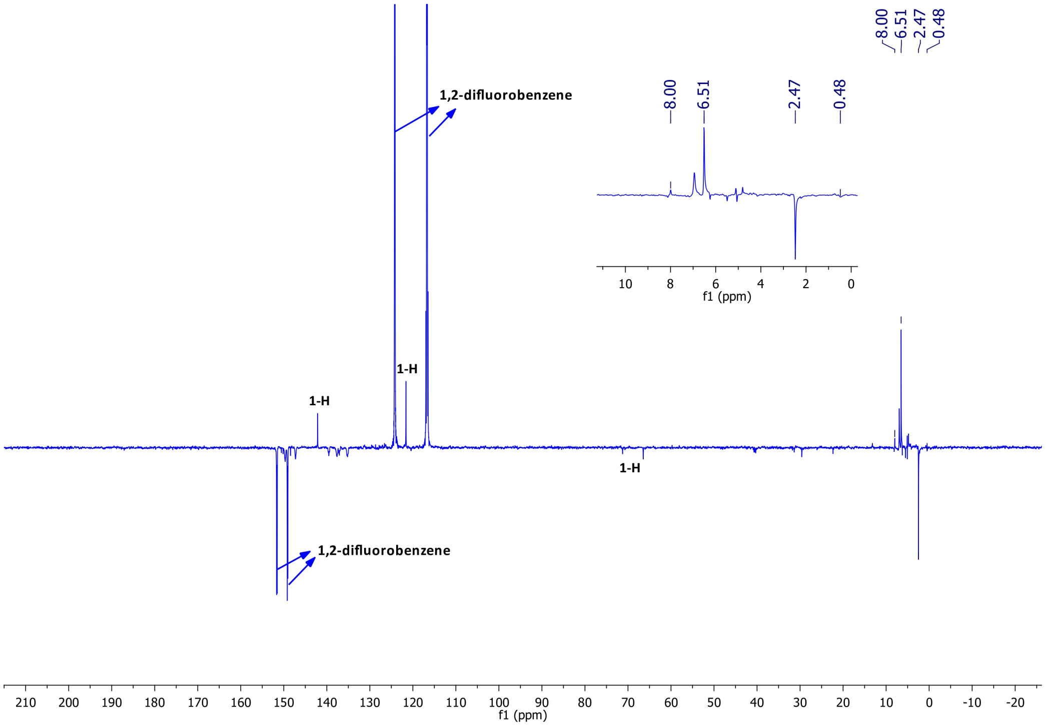

Figure S15. ^{13}C-JMOD NMR spectrum (100 MHz, *o*DFB, DMSO-D$_6$ capillary) of the reaction mixture between [**1**]$^+$[B(C_6F_5)$_4$]$^-$ and Et_3SiH.

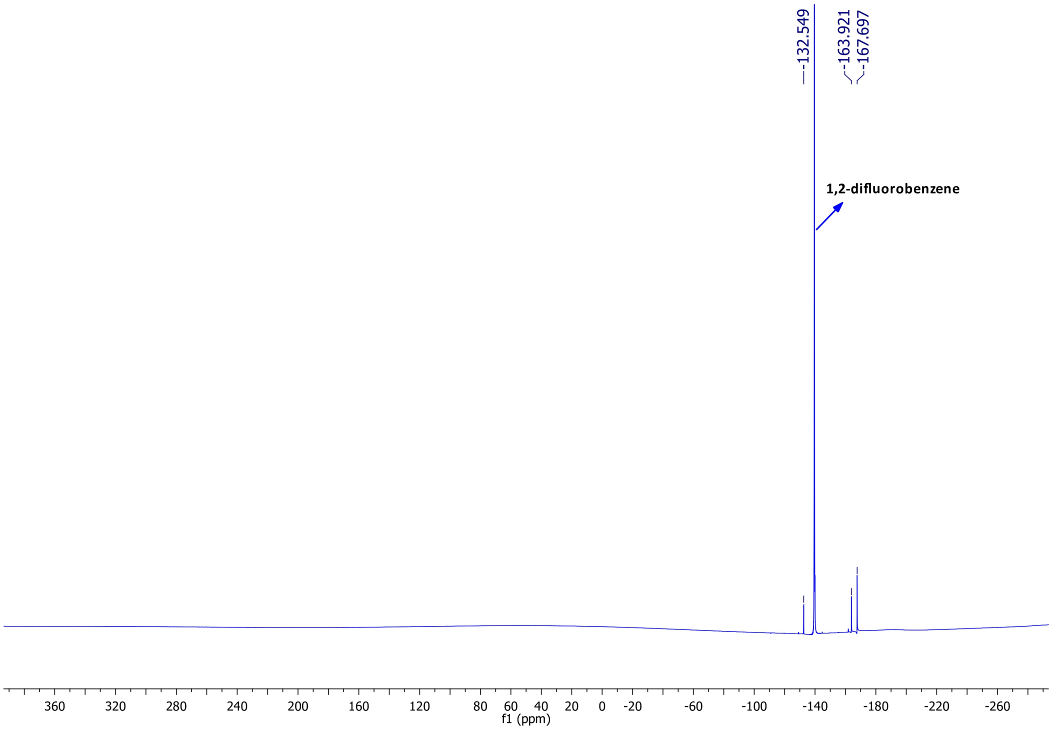

Figure S16. ^{19}F NMR spectrum (376.5 MHz, *o*DFB, DMSO-D_6 capillary) of the reaction mixture between [**1**]$^+$[$B(C_6F_5)_4$]$^-$ and Et_3SiH.

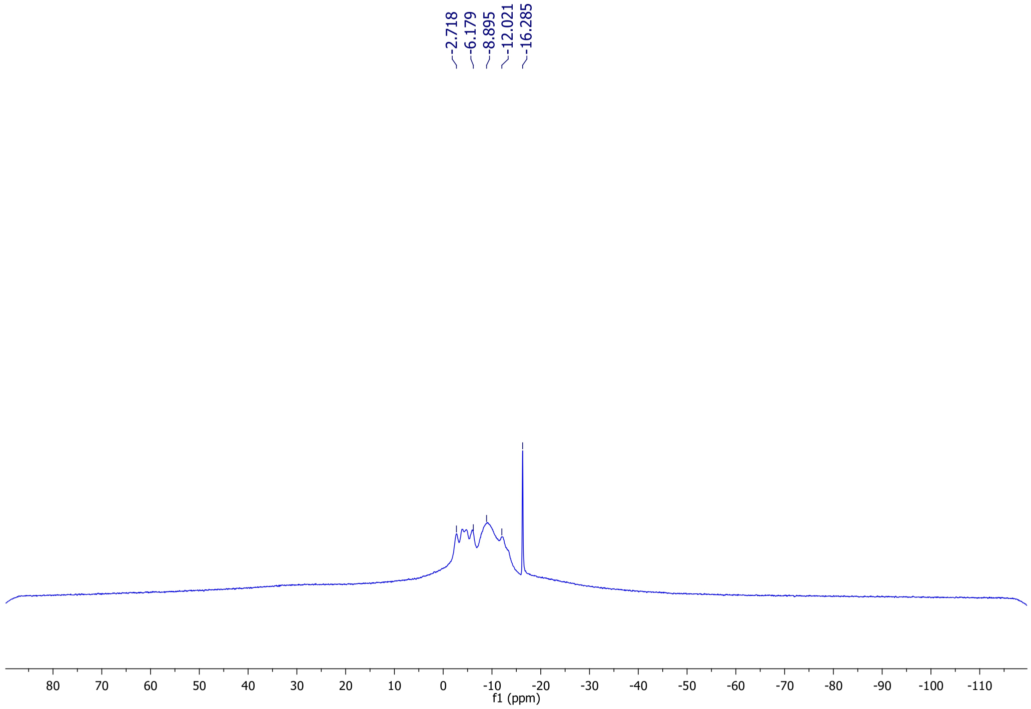

Figure S17. ^{11}B NMR spectrum (128 MHz, *o*DFB, DMSO-D_6 capillary) of the reaction mixture between [**1**]$^+$[$B(C_6F_5)_4$]$^-$ and Et_3SiH.

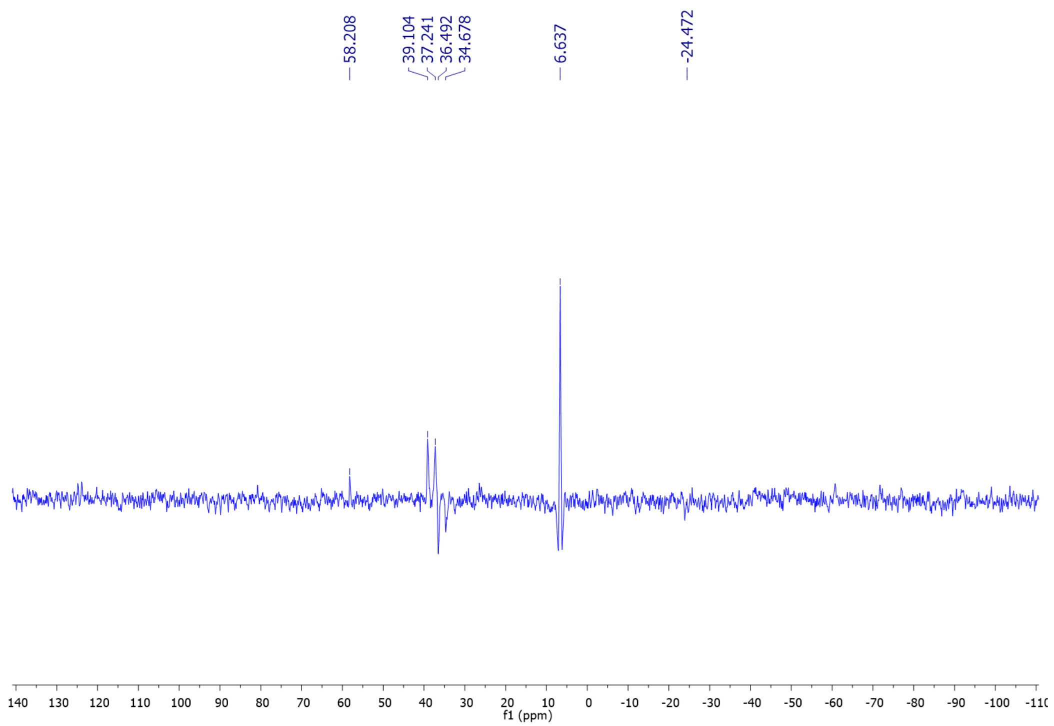

Figure S18. ^{29}Si NMR spectrum (79.5 MHz, *o*DFB, DMSO-D$_6$ capillary) of the reaction mixture between [**1**]$^+$[B(C$_6$F$_5$)$_4$]$^-$ and Et$_3$SiH.

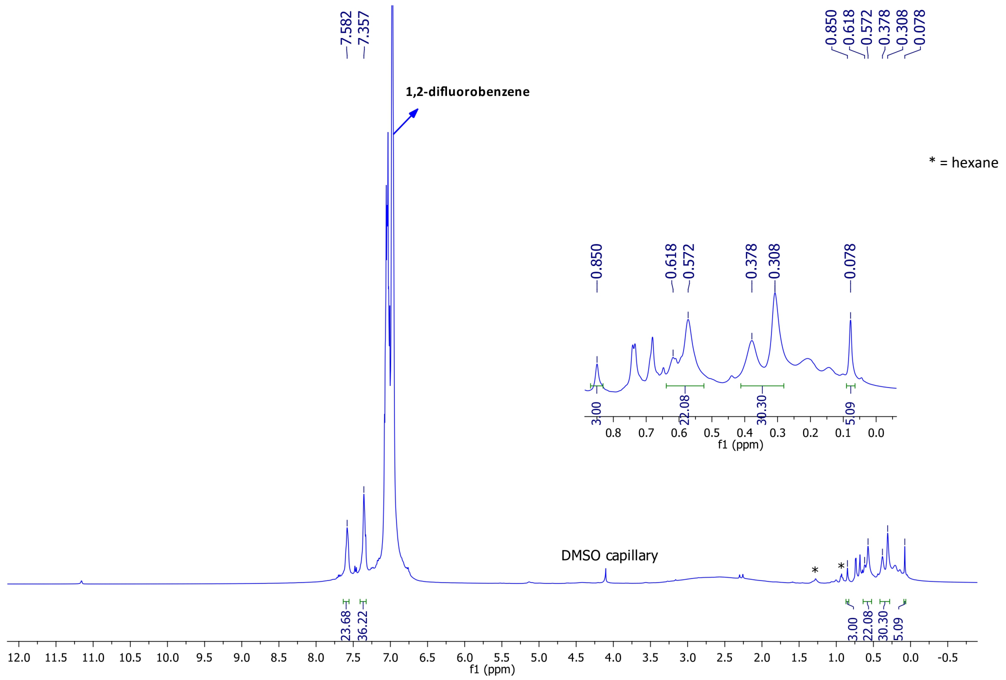

Figure S19. ^{1}H NMR spectrum (400 MHz, *o*DFB, DMSO-D$_6$ capillary) of the reaction mixture between [**1**]$^+$[B(C$_6$F$_5$)$_4$]$^-$ and Me$_2$PhSiH.

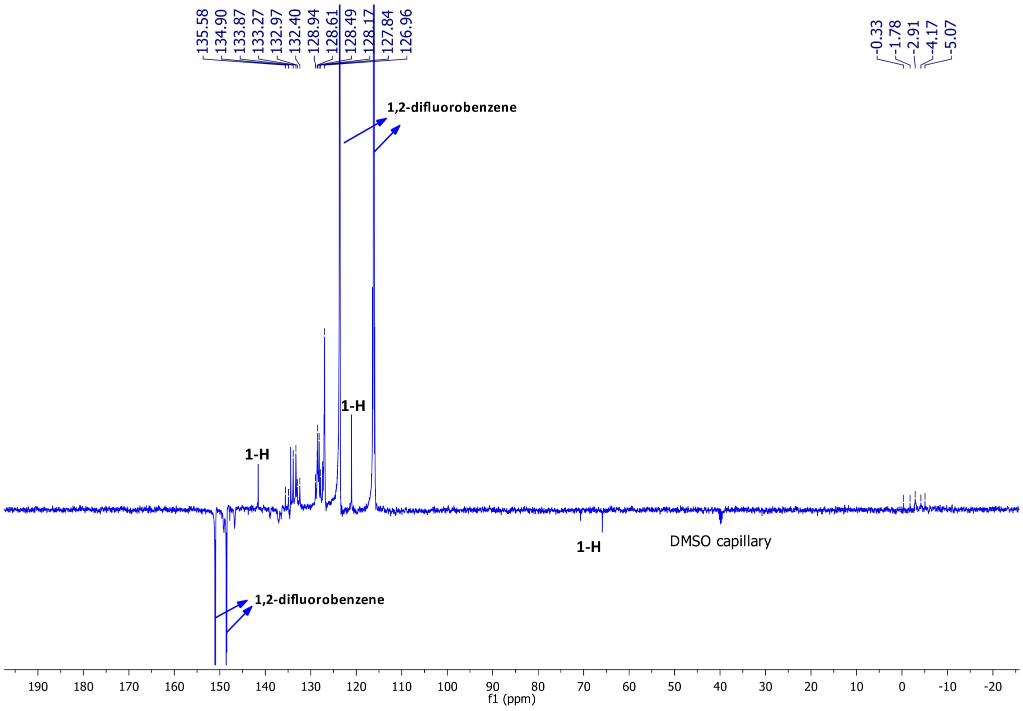

Figure S20. ^{13}C-JMOD NMR spectrum (100 MHz, *o*DFB, DMSO-D$_6$ capillary) of the reaction mixture between [**1**]$^+$[B(C$_6$F$_5$)$_4$]$^-$ and Me$_2$PhSiH.

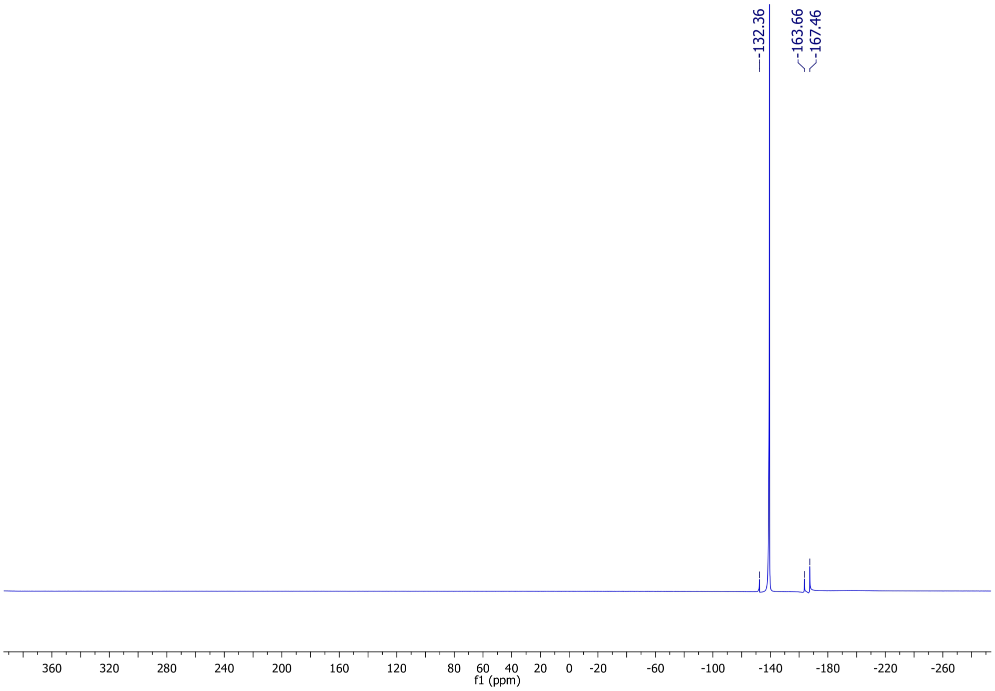

Figure S21. ^{19}F NMR spectrum (376.5 MHz, *o*DFB, DMSO-D$_6$ capillary) of the reaction mixture between [**1**]$^+$[B(C$_6$F$_5$)$_4$]$^-$ and Me$_2$PhSiH.

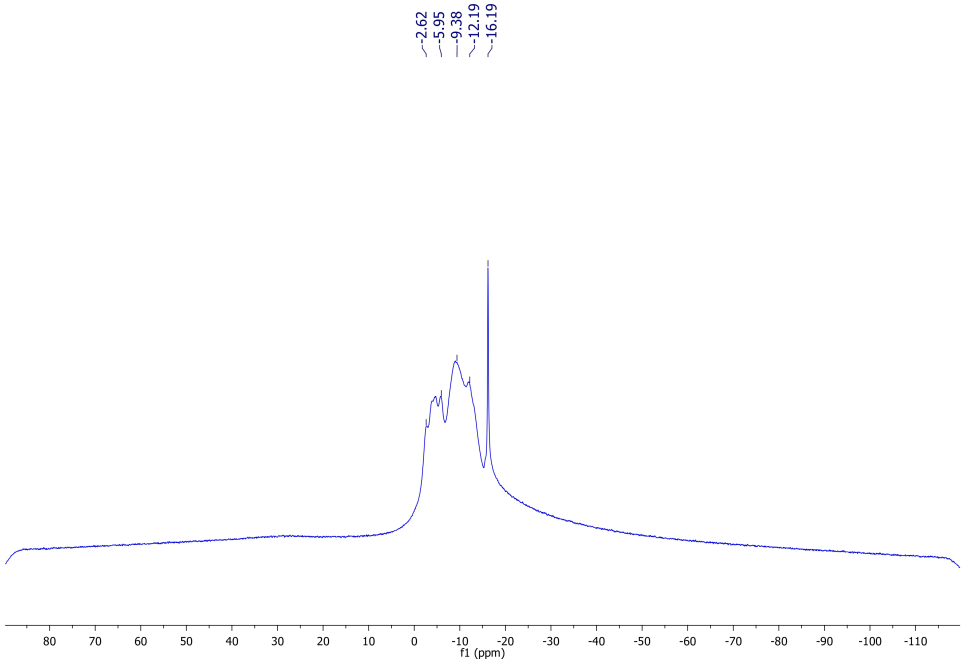

Figure S22. ^{11}B NMR spectrum (128 MHz, *o*DFB, DMSO-D$_6$ capillary) of the reaction mixture between [**1**]$^+$[B(C$_6$F$_5$)$_4$]$^-$ and Me$_2$PhSiH.

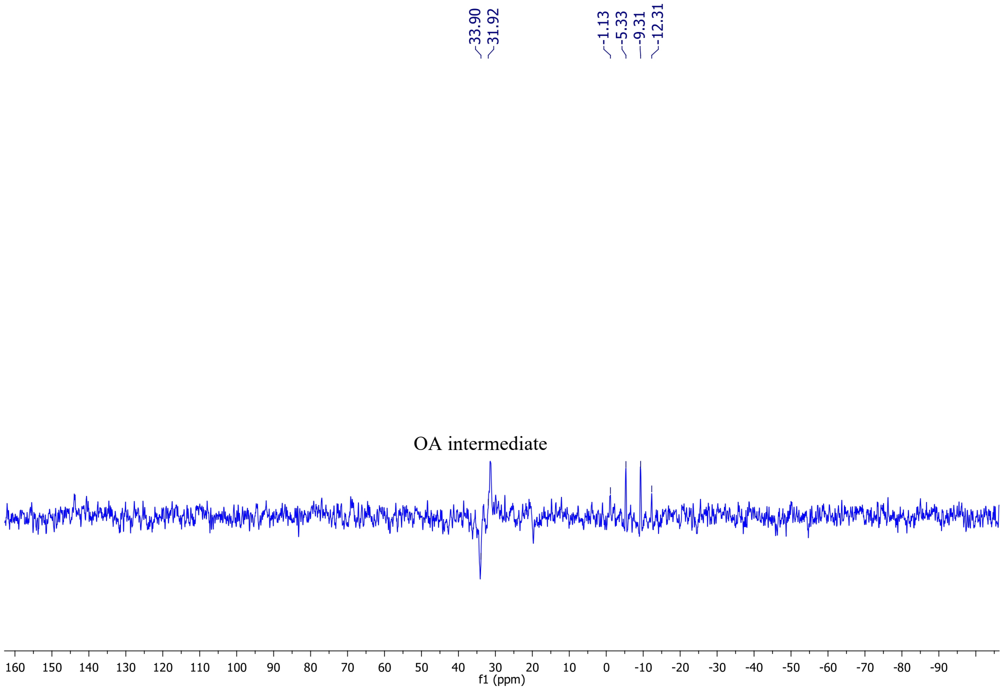

Figure S23. ^{29}Si NMR spectrum (79.5 MHz, *o*DFB, DMSO-D$_6$ capillary) of the reaction mixture between [**1**]$^+$[B(C$_6$F$_5$)$_4$]$^-$ and Me$_2$PhSiH.

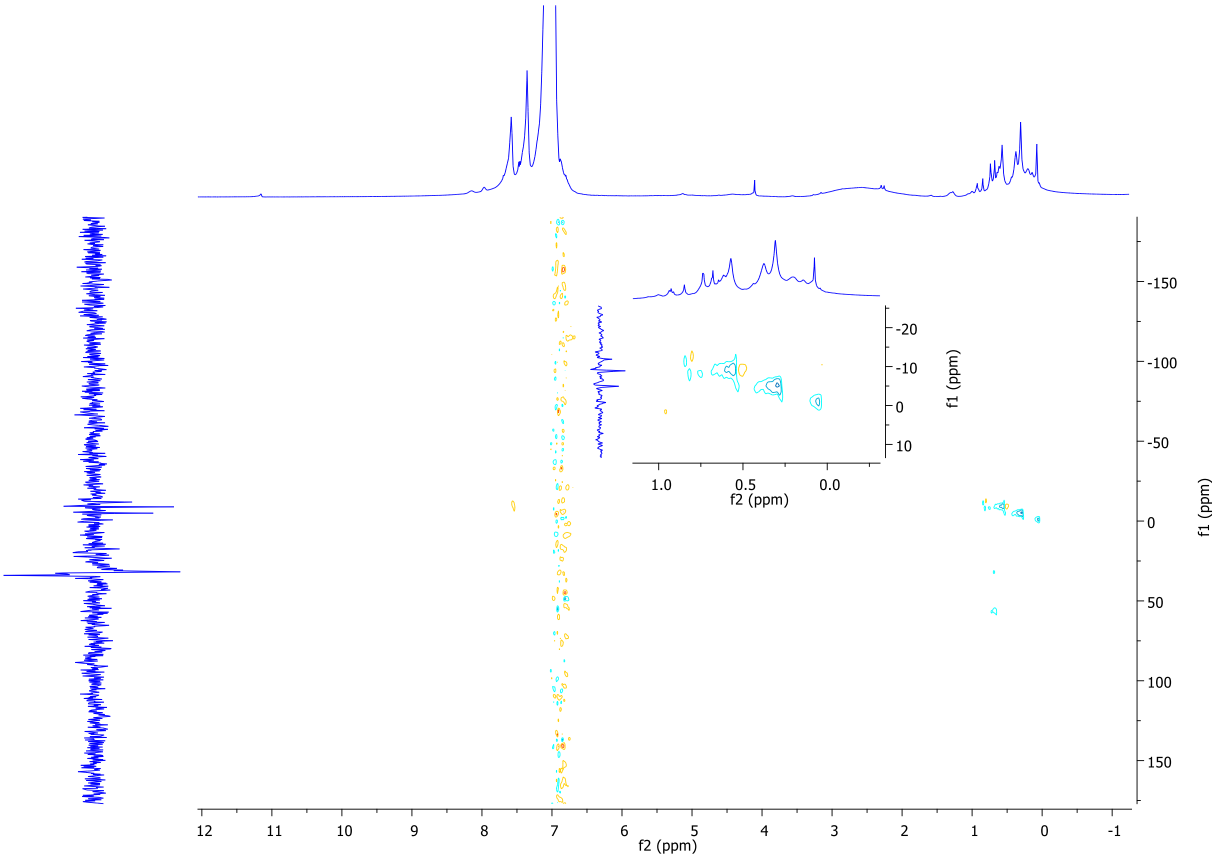

Figure S24. $^{1}H/^{29}Si$ HSQC NMR spectrum (500/79.5 MHz, *o*DFB, DMSO-D_6 capillary) of the reaction mixture between [**1**]$^+$[B(C_6F_5)$_4$]$^-$ and Me_2PhSiH.

<u>GC-MS data:</u>

Integration Peak List

Peak	RT	Height	Area	Area%	Area Sum%
1	1.504	663940.19	449586.22	3.12	0.79
2	11.367	4324881.42	8866367.24	61.61	15.5
5	26.705	6090514.2	14390592	100	25.16
8	34.739	4709175.67	5142079.59	35.73	8.99

Compound Table

Compound Label	RT	Name	DB Formula	Hits (DB)
Cpd 1: Silane, tetramethyl-	1.504	Silane, tetramethyl-	C4H12Si	3
Cpd 12: Silane, trimethylphenyl-	11.367	Silane, trimethylphenyl-	C9H14Si	3
Cpd 15: Dimethyl diphenylsilane	26.705	Dimethyl diphenylsilane	C14H16Si	3
Cpd 19: Silane, methyltriphenyl-	34.739	Silane, methyltriphenyl-	C19H18Si	3

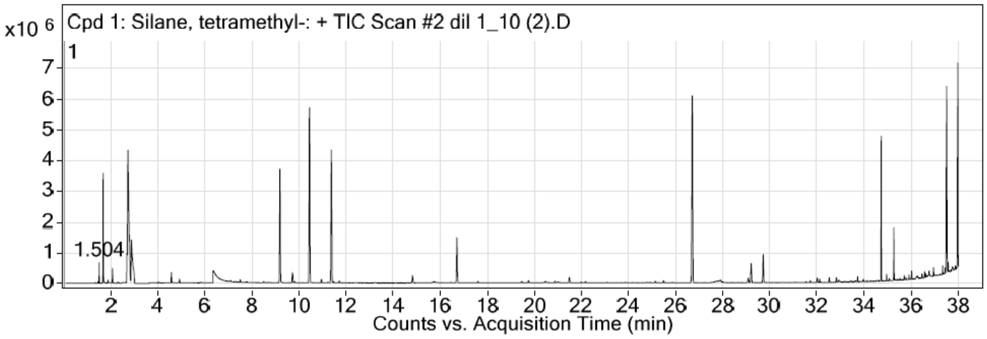

MS Spectrum

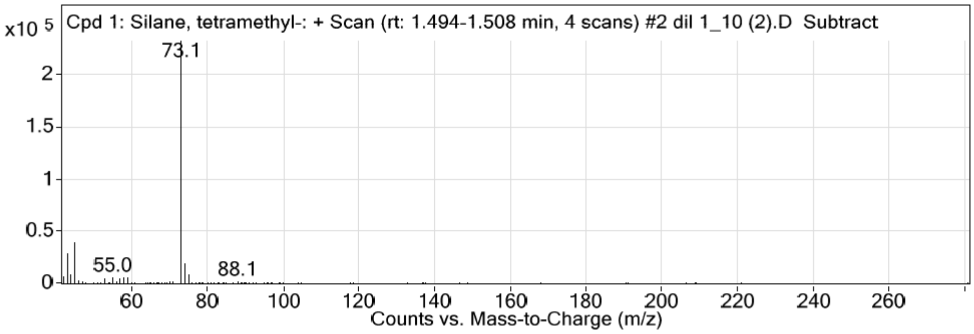

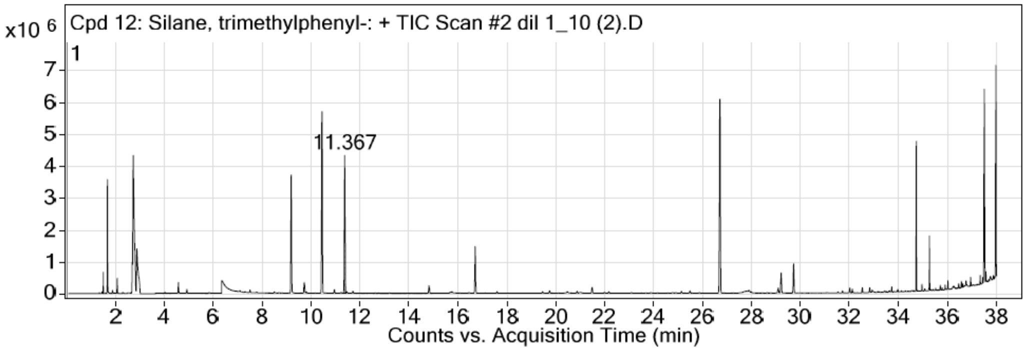

MS Spectrum

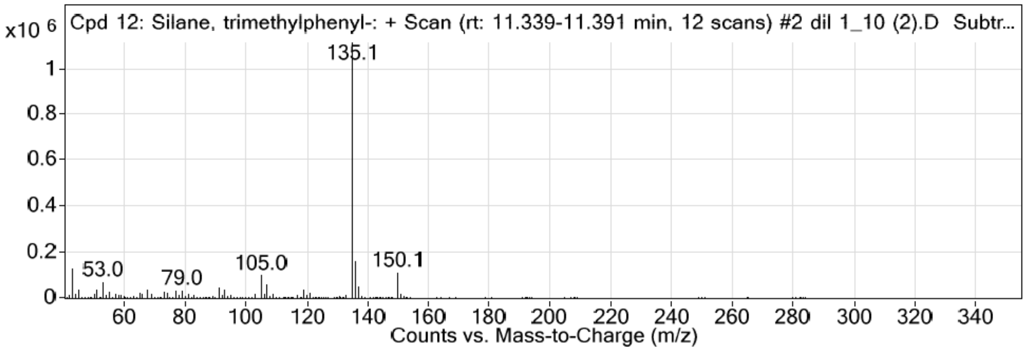

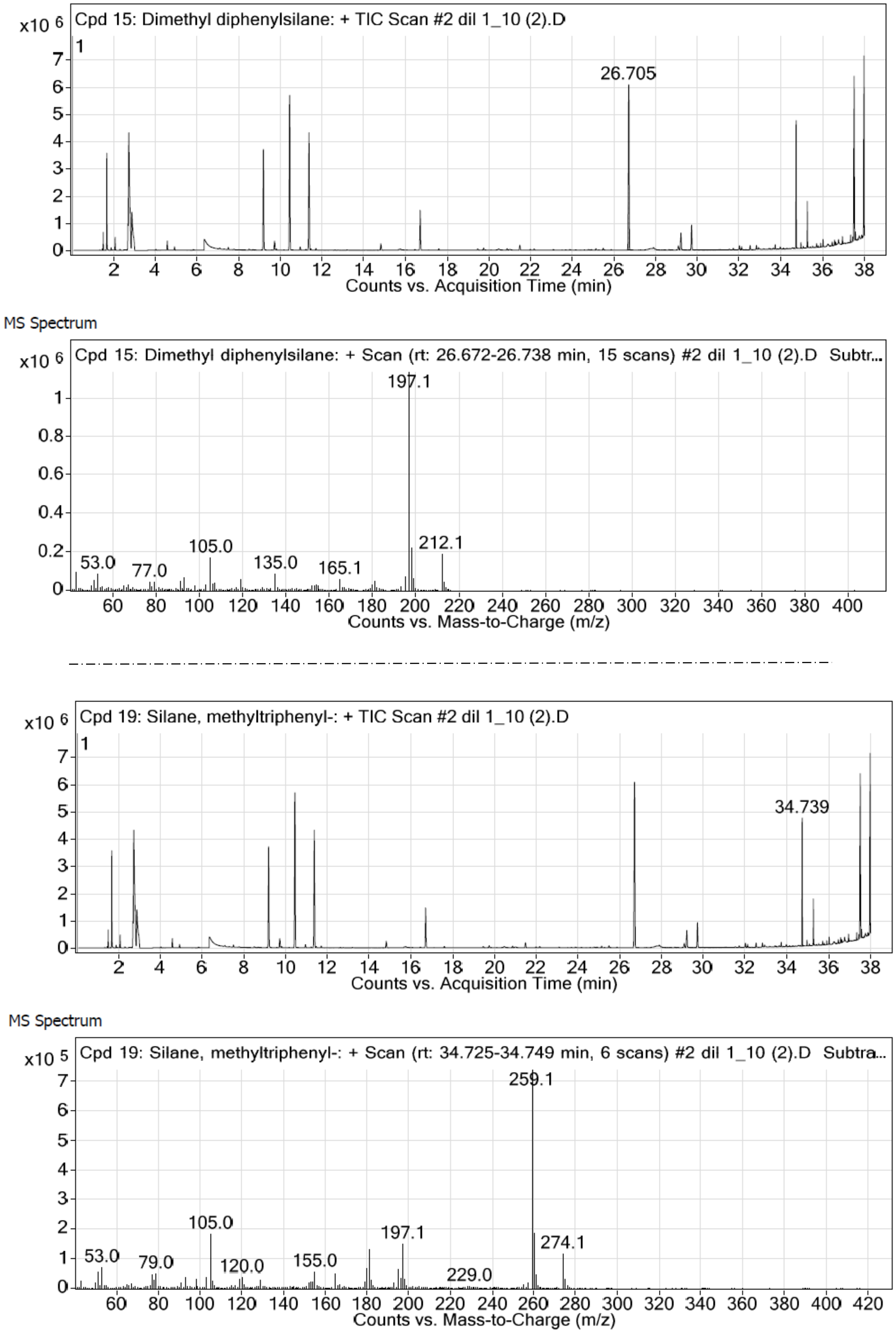

Figure S25. GC-MS data of the reaction mixture between $[\mathbf{1}]^{+}[B(C_6F_5)_4]^{-}$ and Me_2PhSiH (1:10 ratio).

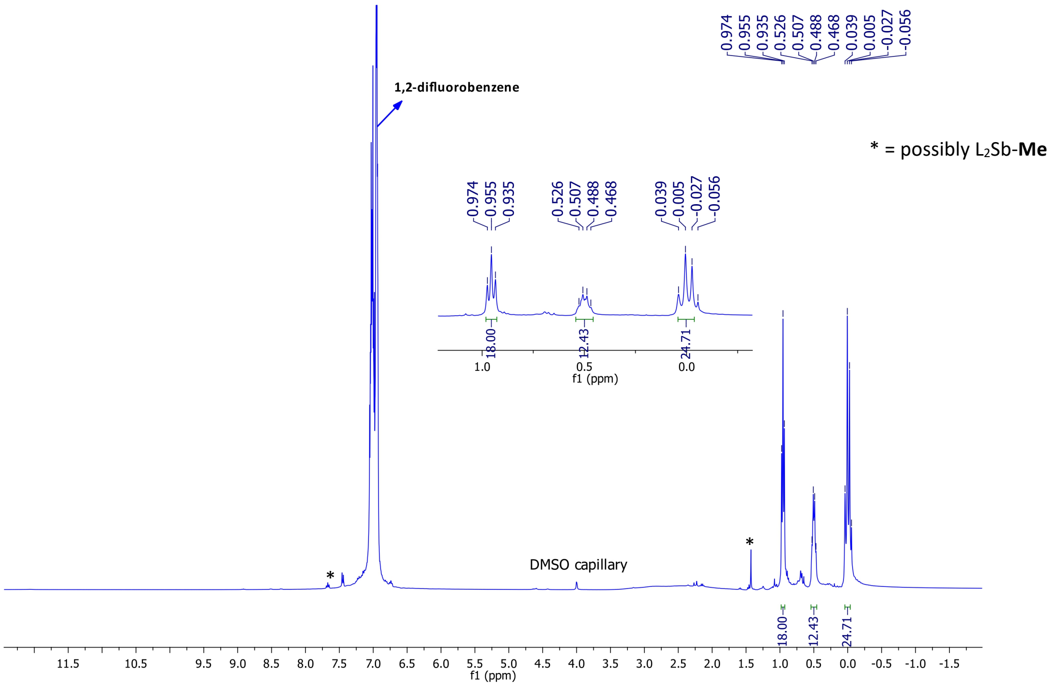

Figure S26. ^{1}H NMR spectrum (400 MHz, *o*DFB, DMSO-D_6 capillary) of the reaction mixture between $[\mathbf{1}]^{+}[B(C_6F_5)_4]^{-}$ and a mixture of Et_4Si and Me_4Si.

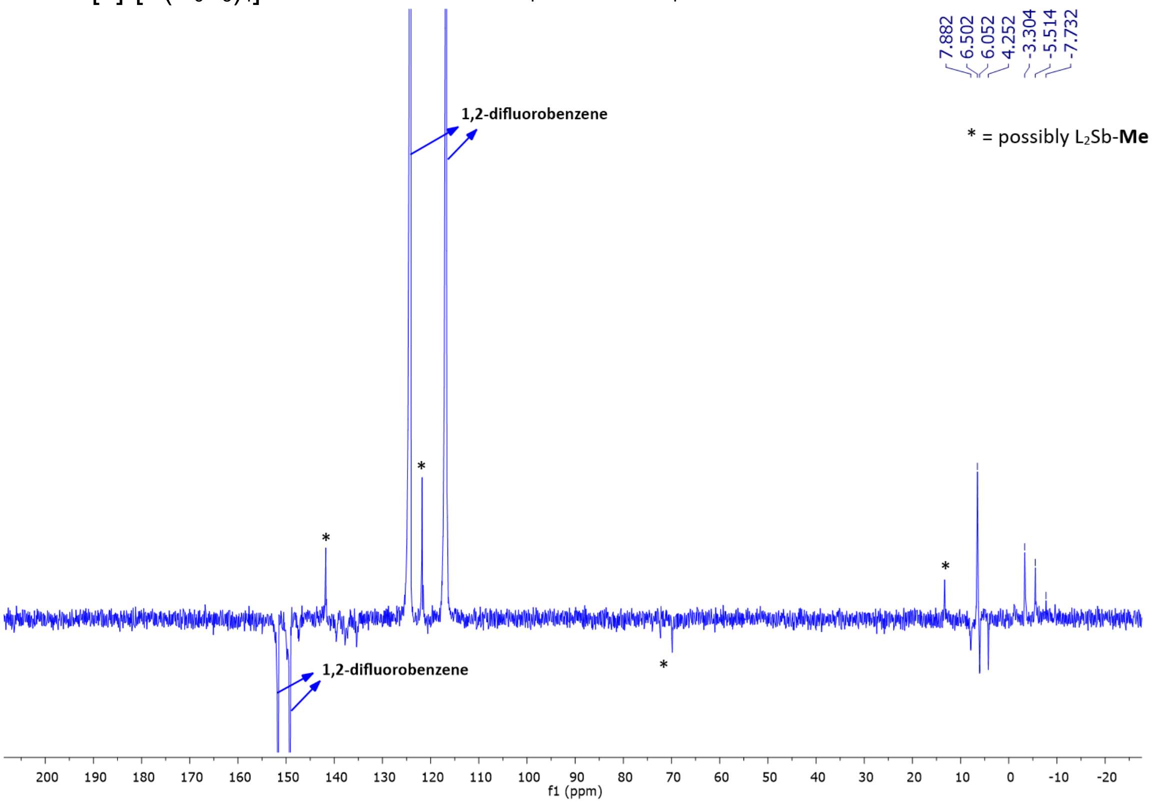

Figure S27. ^{13}C-JMOD NMR spectrum (100 MHz, *o*DFB, DMSO-D_6 capillary) of the reaction mixture between $[\mathbf{1}]^{+}[B(C_6F_5)_4]^{-}$ and a mixture of Et_4Si and Me_4Si.

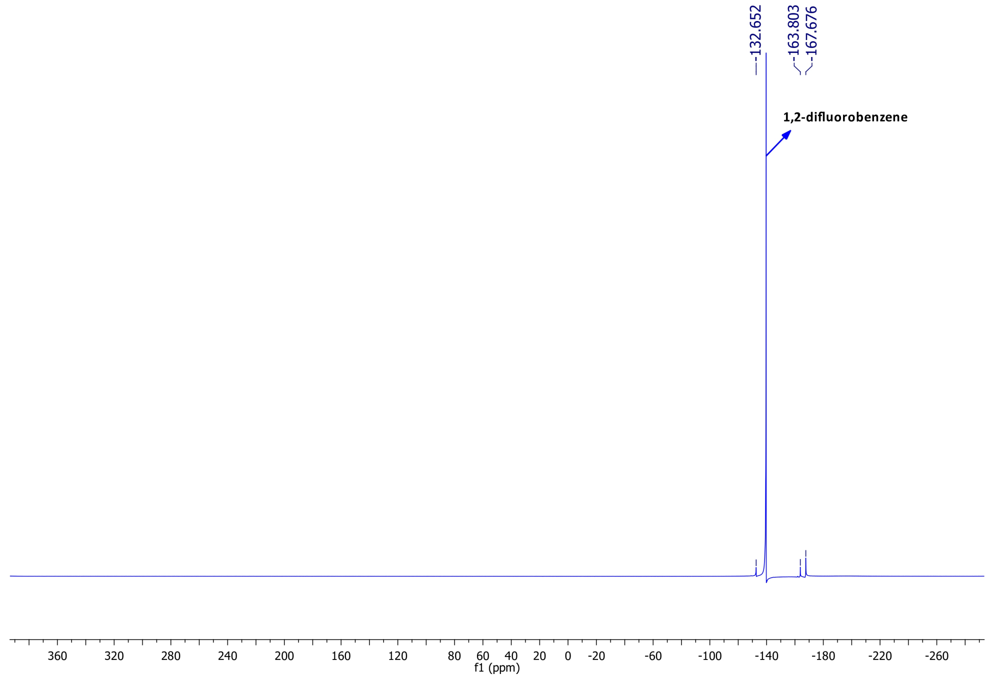

Figure S28. ^{19}F NMR spectrum (376.5 MHz, *o*DFB, DMSO-D_6 capillary) of the reaction mixture between [**1**]$^+$[$B(C_6F_5)_4$]$^-$ and a mixture of Et_4Si and Me_4Si.

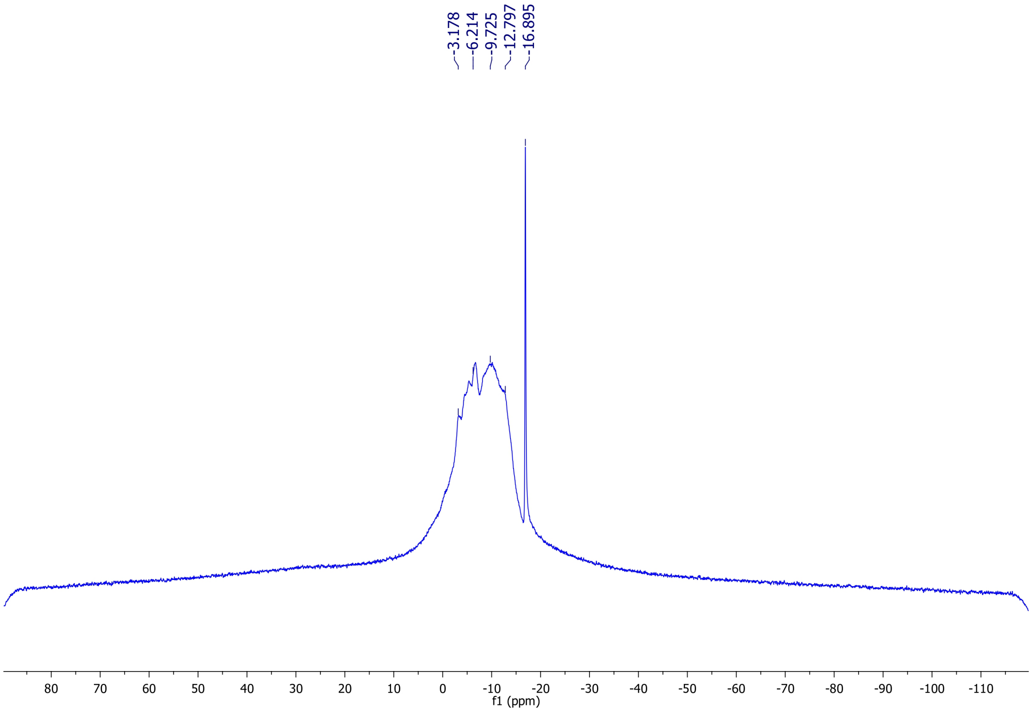

Figure S29. ^{11}B NMR spectrum (128 MHz, *o*DFB, DMSO-D_6 capillary) of the reaction mixture between [**1**]$^+$[$B(C_6F_5)_4$]$^-$ and a mixture of Et_4Si and Me_4Si.

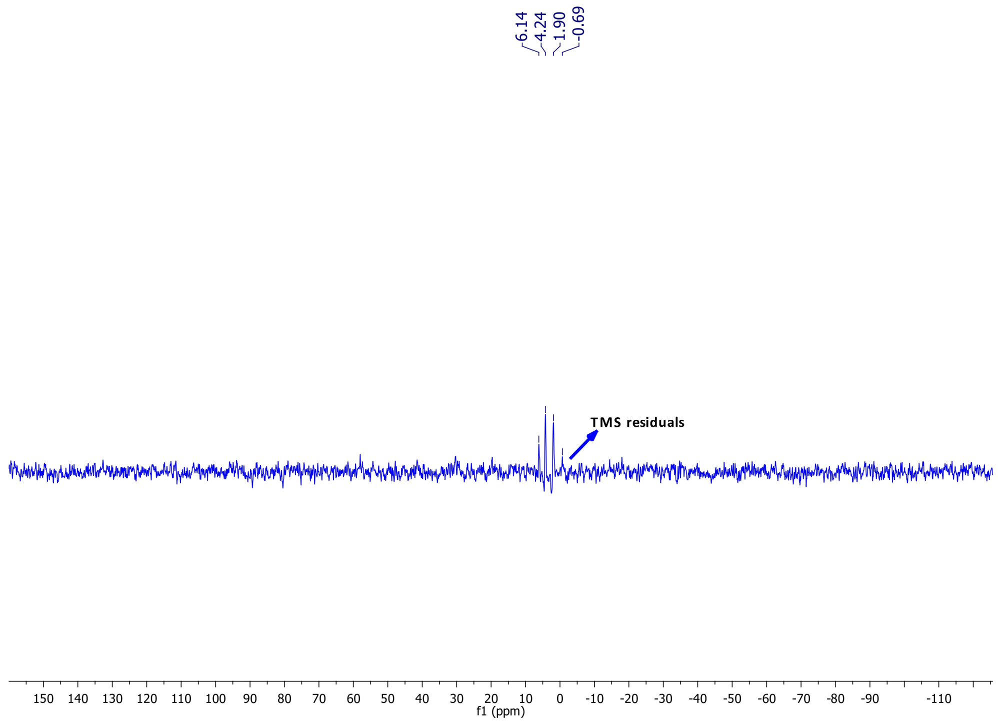

Figure S30. ^{29}Si NMR spectrum (79.5 MHz, *o*DFB, DMSO-D$_6$ capillary) of the reaction mixture between [**1**]$^+$[B(C$_6$F$_5$)$_4$]$^-$ and a mixture of Et$_4$Si and Me$_4$Si.

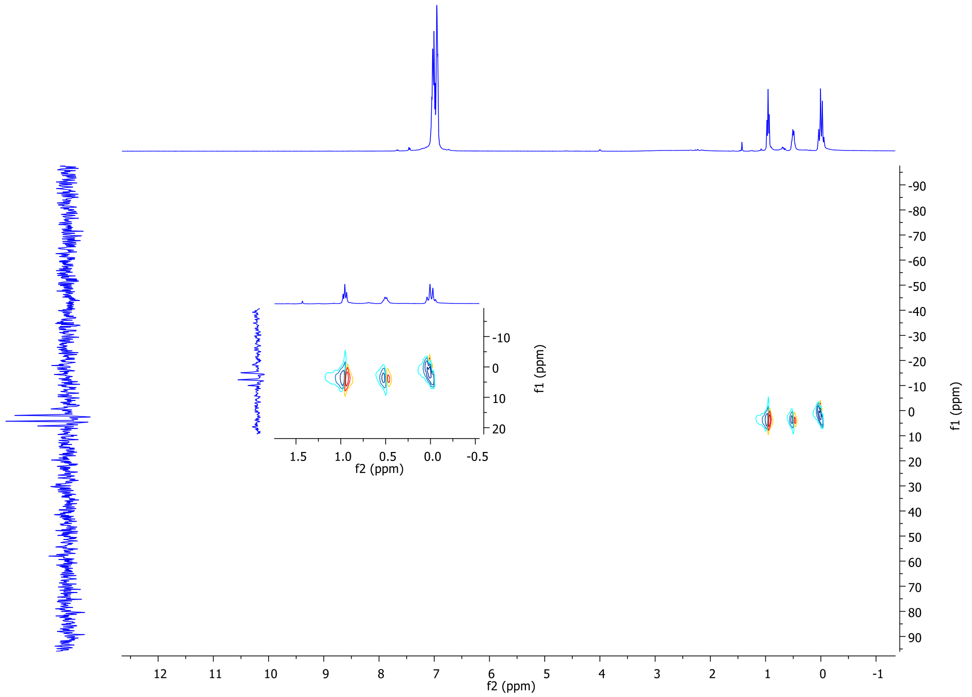

Figure S31. ^{1}H/^{29}Si HSQC NMR spectrum (500/79.5 MHz, *o*DFB, DMSO-D$_6$ capillary) of the reaction mixture between [**1**]$^+$[B(C$_6$F$_5$)$_4$]$^-$ and a mixture of Et$_4$Si and Me$_4$Si.

GC-MS data:

Integration Peak List

Peak	RT	Height	Area	Area %	Area SUM %
1	1.501	4734002.62	3048981.48	9.21	4.31
2	1.879	21737535.01	20425994.39	61.67	28.91
3	3.002	13297836.28	33122304.41	100	46.87
4	5.655	7260258.38	14066919.57	42.47	19.91

Compound Table

Compound Label	RT	Name	DB Formula	Hits (DB)
Cpd 1: Silane, tetramethyl-	1.501	Silane, tetramethyl-	C4H12Si	3
Cpd 2: Silane, ethyltrimethyl-	1.879	Silane, ethyltrimethyl-	C5H14Si	3
Cpd 5: Silane, diethyldimethyl	3.002	Silane, diethyldimethyl-	C6H16Si	3
Cpd 9: Triethylmethylsilane	5.655	Triethylmethylsilane	C7H18Si	3

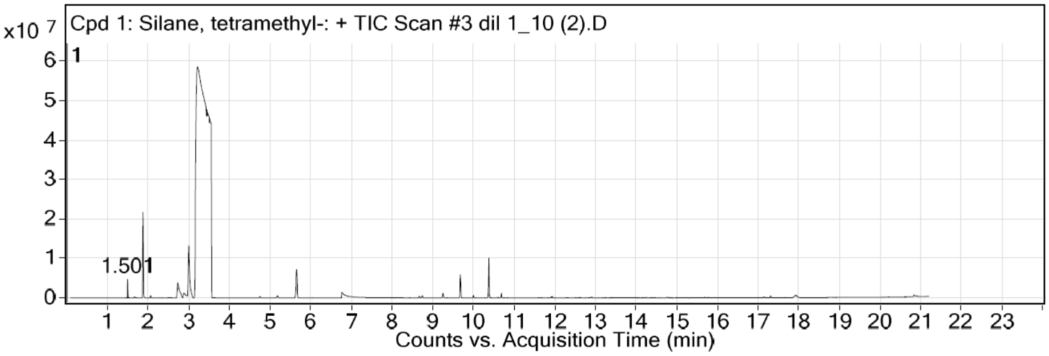

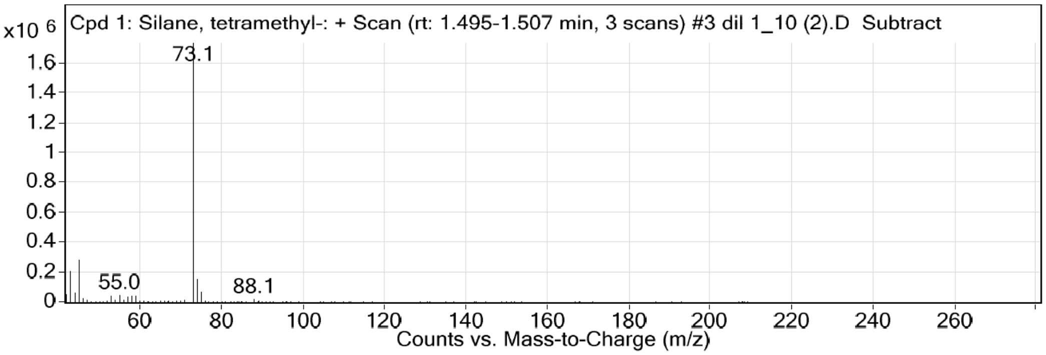

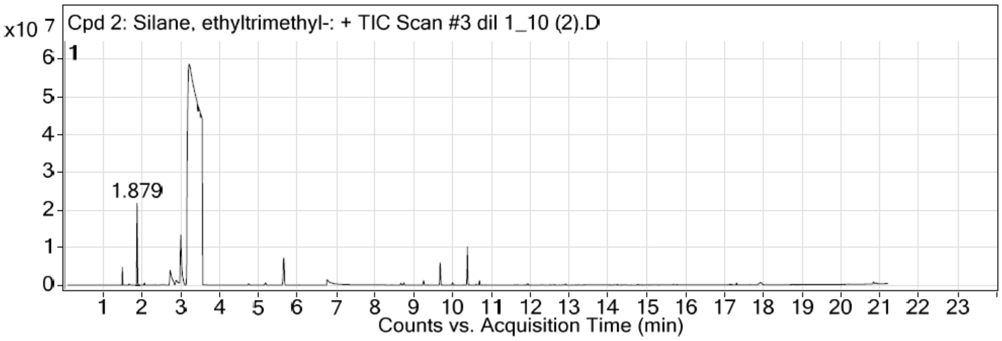

MS Spectrum

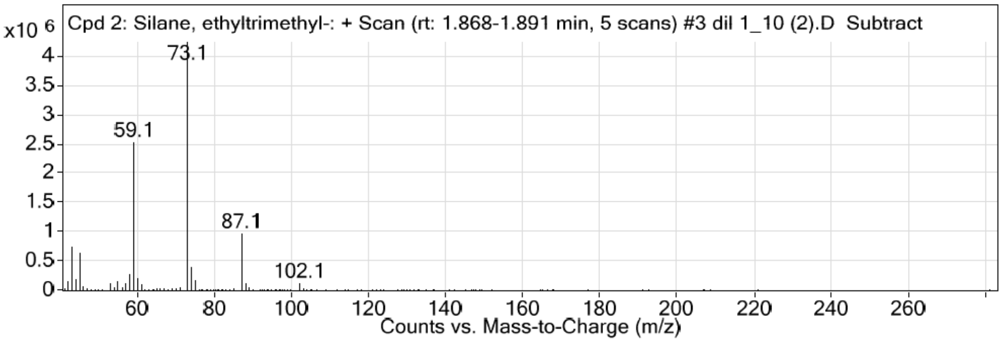

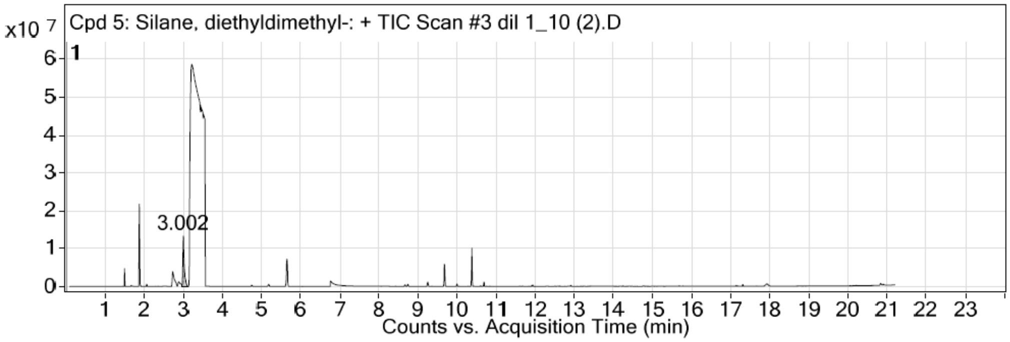

MS Spectrum

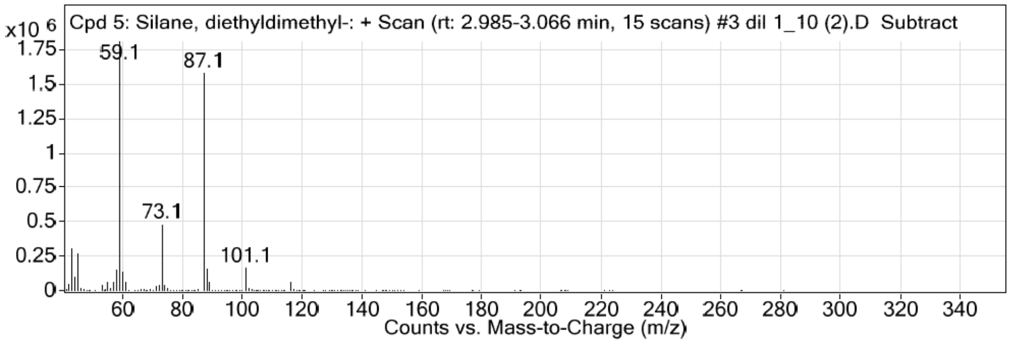

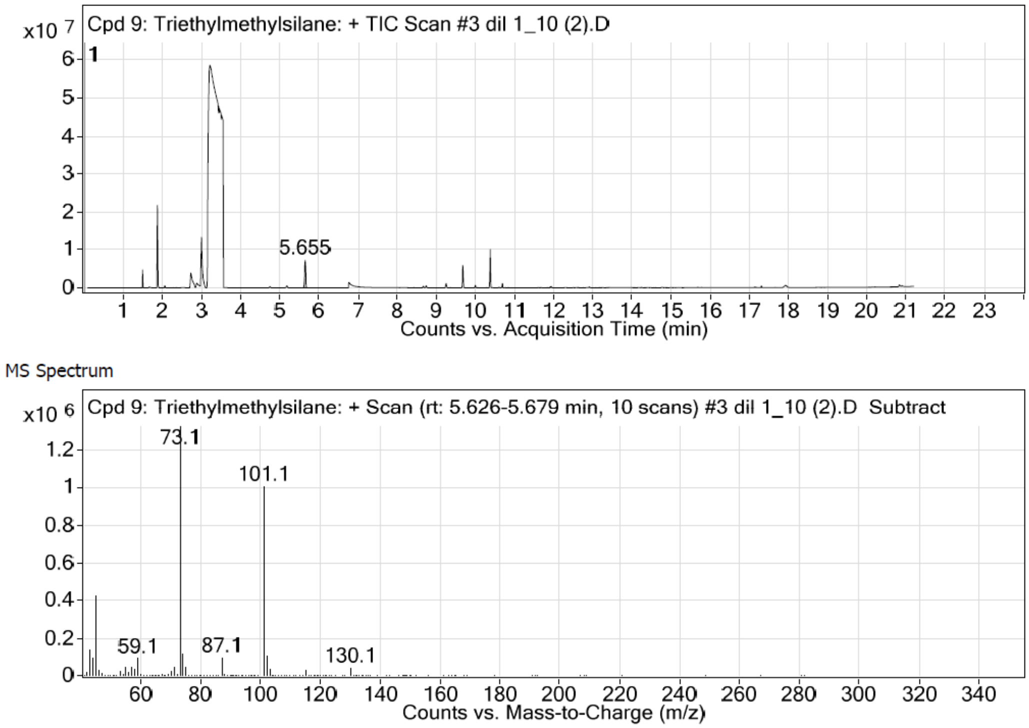

Figure S32. GC-MS data of the reaction mixture between [**1**]$^+$[B(C_6F_5)$_4$]$^-$ and a mixture of Et_4Si and Me_4Si.

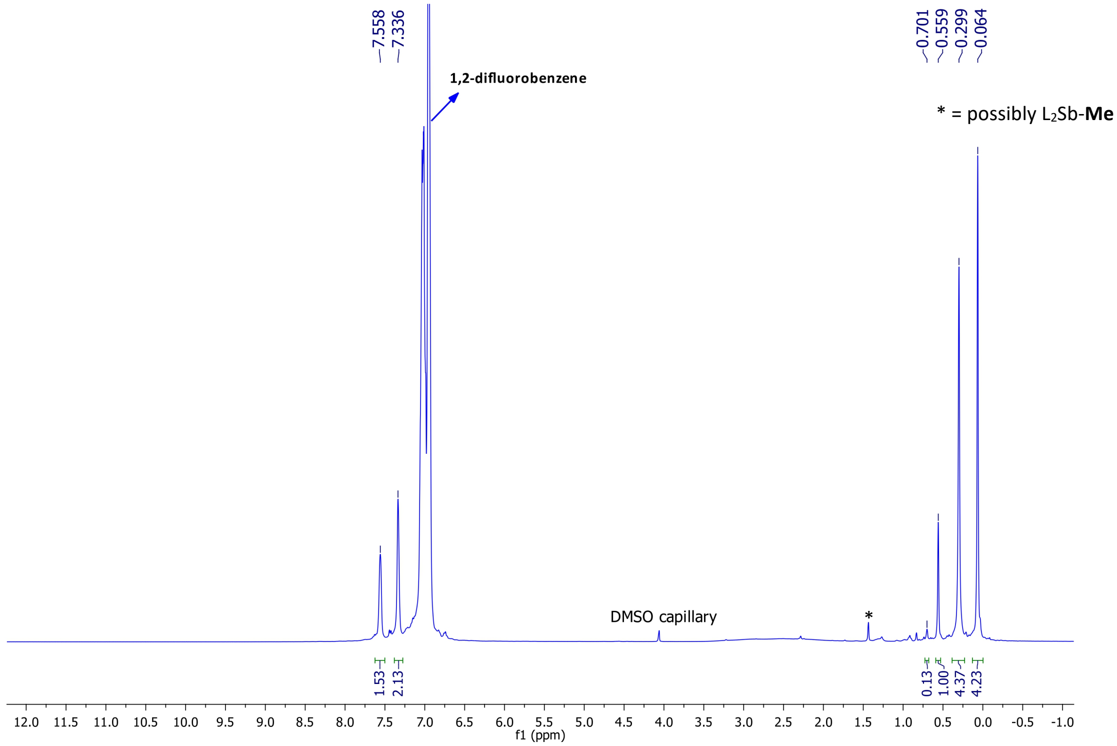

Figure S33. ^{1}H NMR spectrum (400 MHz, *o*DFB, DMSO-D_6 capillary) of the reaction mixture between [**1**]$^+$[B(C_6F_5)$_4$]$^-$ and a mixture of Ph_4Si and Me_4Si.

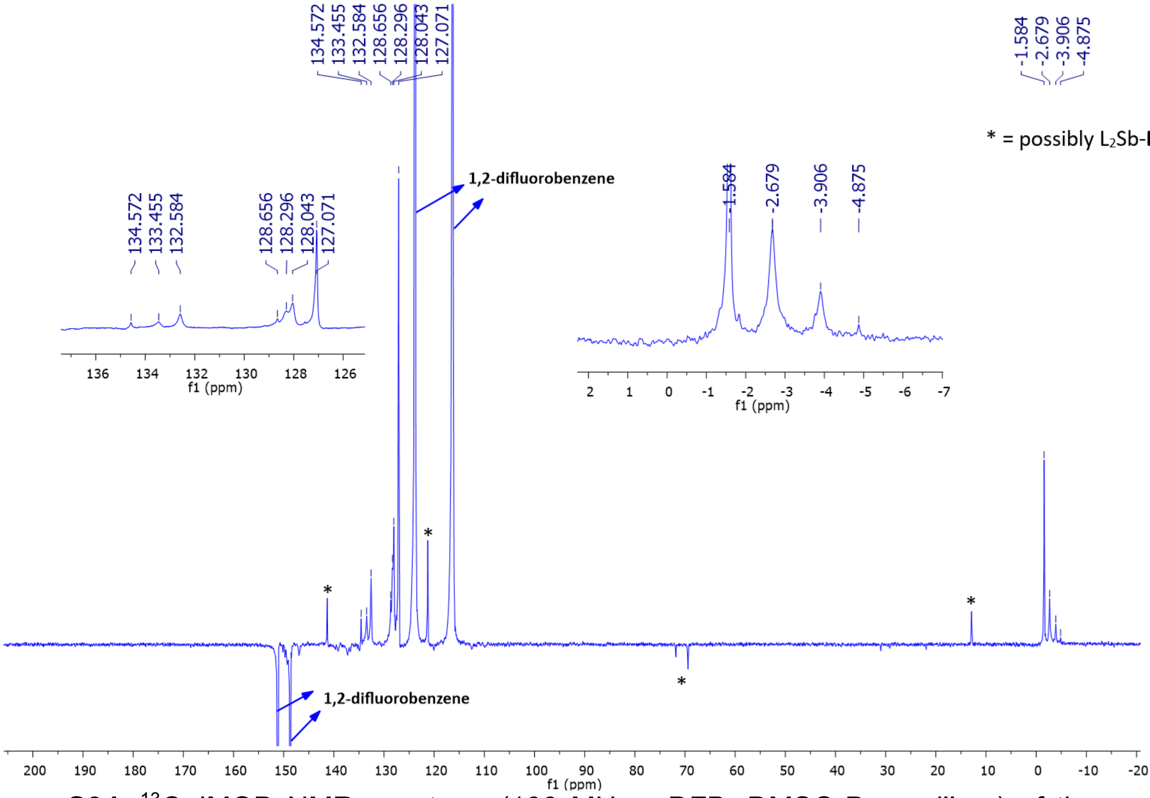

Figure S34. ^{13}C-JMOD NMR spectrum (100 MHz, *o*DFB, DMSO-D$_6$ capillary) of the reaction mixture between [**1**]$^+$[B(C$_6$F$_5$)$_4$]$^-$ and a mixture of Ph$_4$Si and Me$_4$Si.

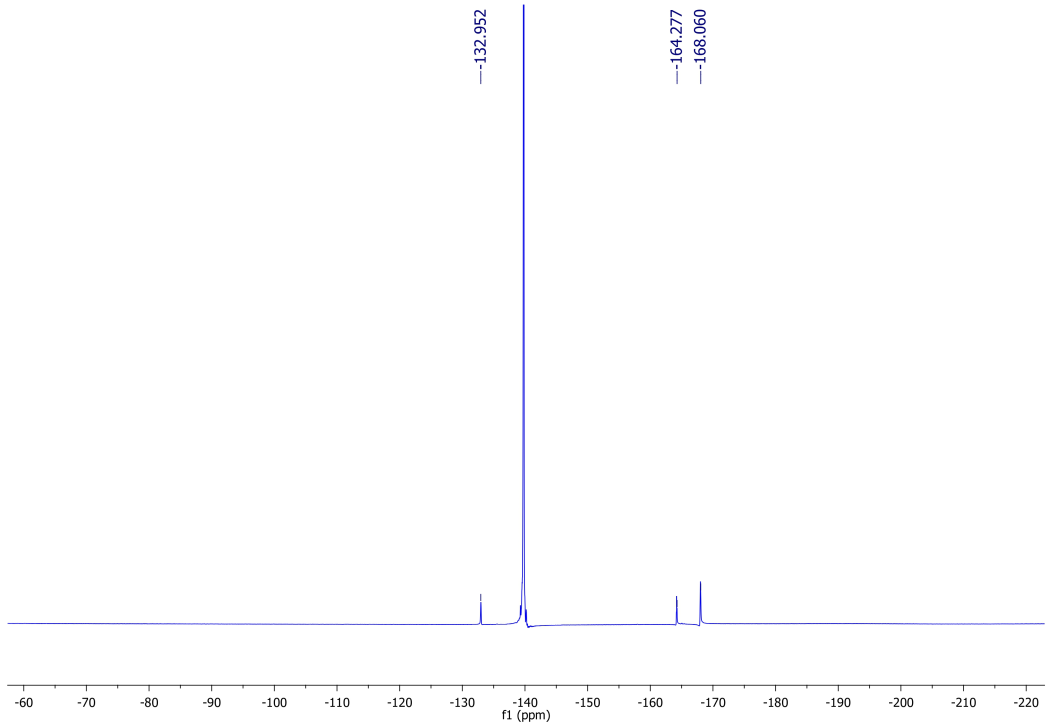

Figure S35. ^{19}F NMR spectrum (376.5 MHz, *o*DFB, DMSO-D$_6$ capillary) of the reaction mixture between [**1**]$^+$[B(C$_6$F$_5$)$_4$]$^-$ and a mixture of Ph$_4$Si and Me$_4$Si.

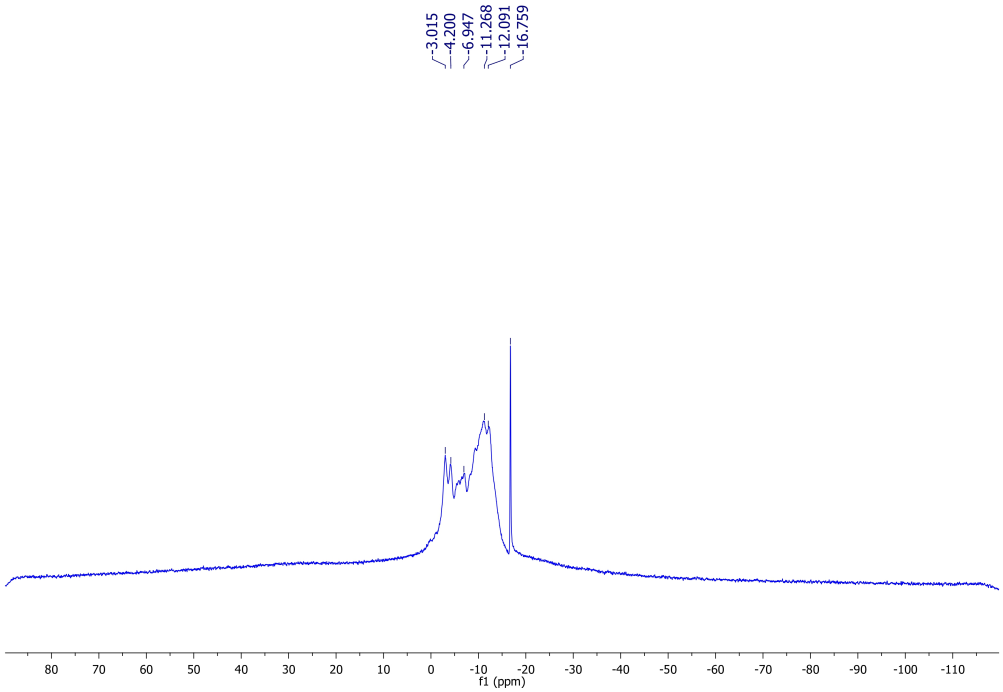

Figure S36. ^{11}B NMR spectrum (128 MHz, *o*DFB, DMSO-D$_6$ capillary) of the reaction mixture between [**1**]$^+$[B(C_6F_5)$_4$]$^-$ and a mixture of Ph_4Si and Me_4Si.

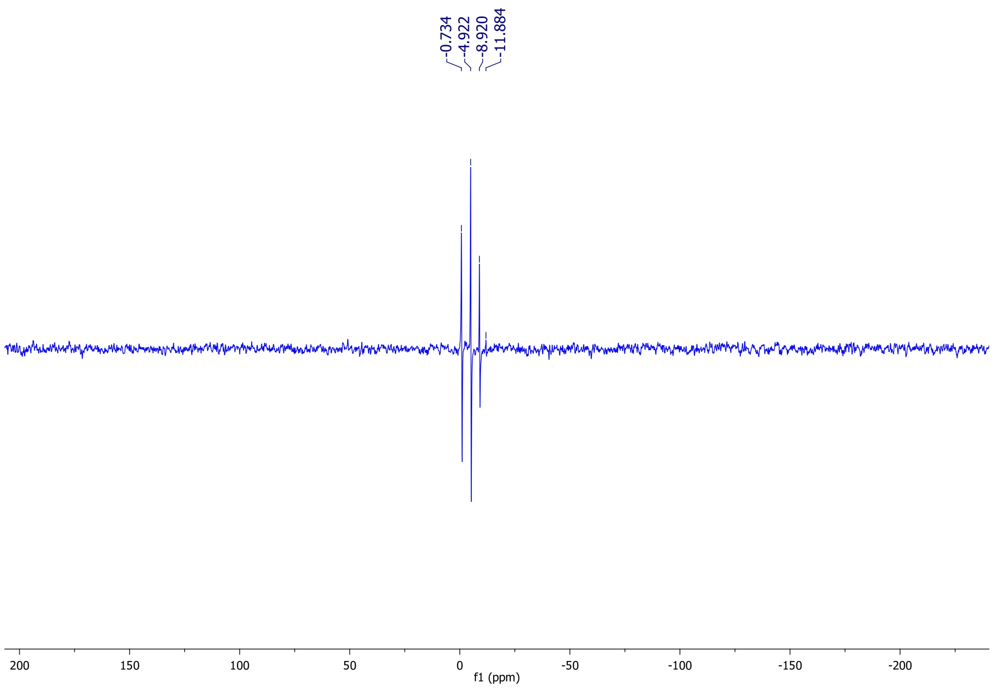

Figure S37. ^{29}Si NMR spectrum (79.5 MHz, *o*DFB, DMSO-D$_6$ capillary) of the reaction mixture between [**1**]$^+$[B(C_6F_5)$_4$]$^-$ and a mixture of Ph_4Si and Me_4Si.

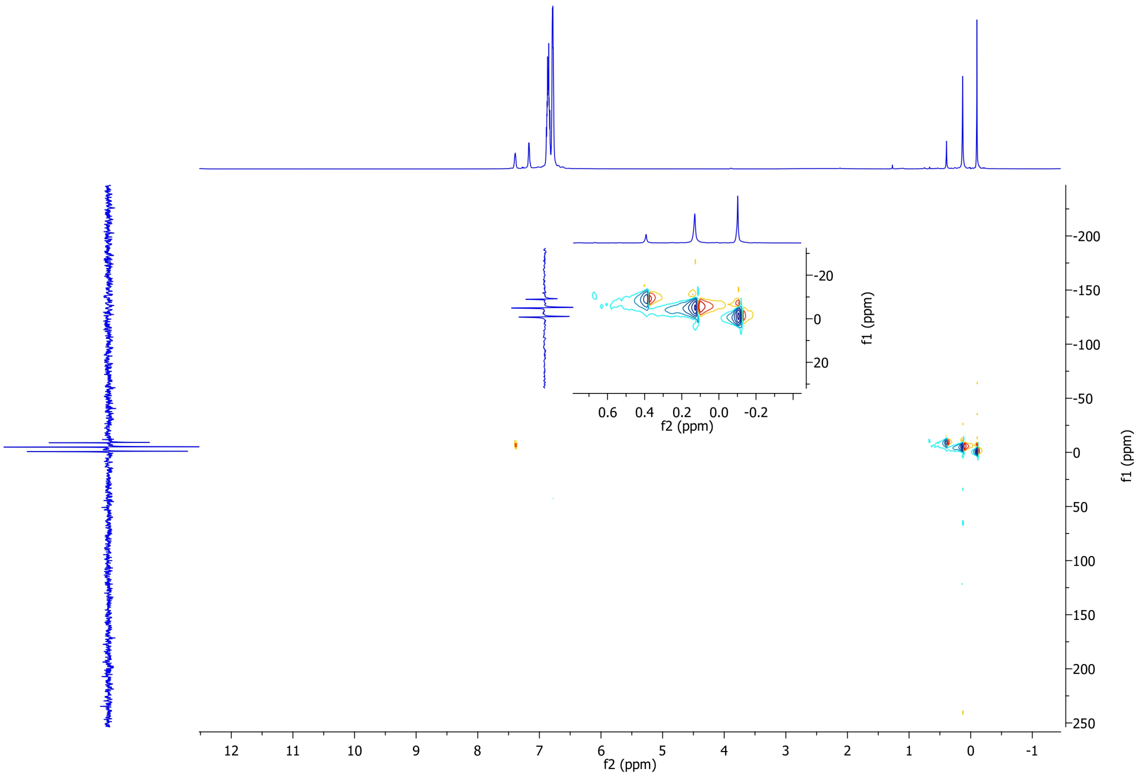

Figure S38. $^{1}H/^{29}Si$ HSQC NMR spectrum (500/79.5 MHz, *o*DFB, DMSO-D_6 capillary) of the reaction mixture between [**1**]$^{+}$[$B(C_6F_5)_4$]$^{-}$ and a mixture of Ph_4Si and Me_4Si.

<u>GC-MS data:</u>

Compound Table

Compound Label	RT	Name	DB Formula	Hits (DB)
Cpd 4: Silane, trimethylphenyl-	4.748	Silane, trimethylphenyl-	C9H14Si	3
Cpd 5: Dimethyl diphenylsilane	10.29	Dimethyldiphenyl Silane	C14H16Si	3
Cpd 6: Silane, methyltriphenyl-	14.161	Silane, methyltriphenyl-	C19H18Si	3

Integration Peak List

Peak	RT	Height	Area	Area%	Area Sum%
4	4.748	13948635.18	16270770.25	66.92	18.5
5	10.29	20852571.65	24312696.18	100	27.64
6	14.161	9703040.67	11249049.19	46.27	12.79

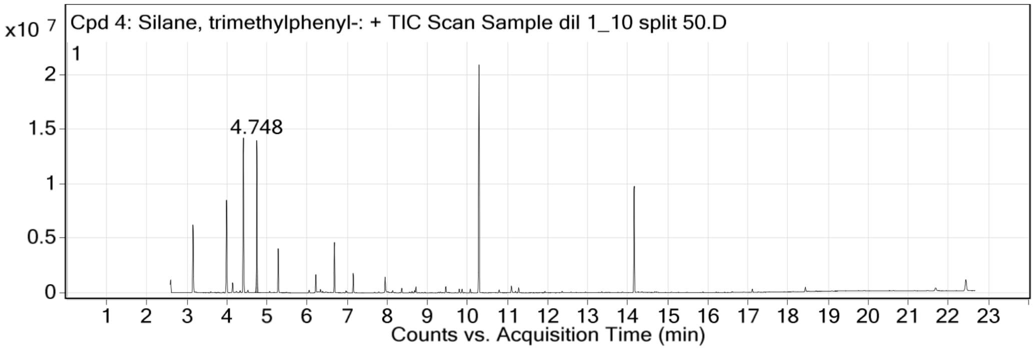
Cpd 4: Silane, trimethylphenyl-: + TIC Scan Sample dil 1_10 split 50.D
x10 7
4.748
Counts vs. Acquisition Time (min)

MS Spectrum

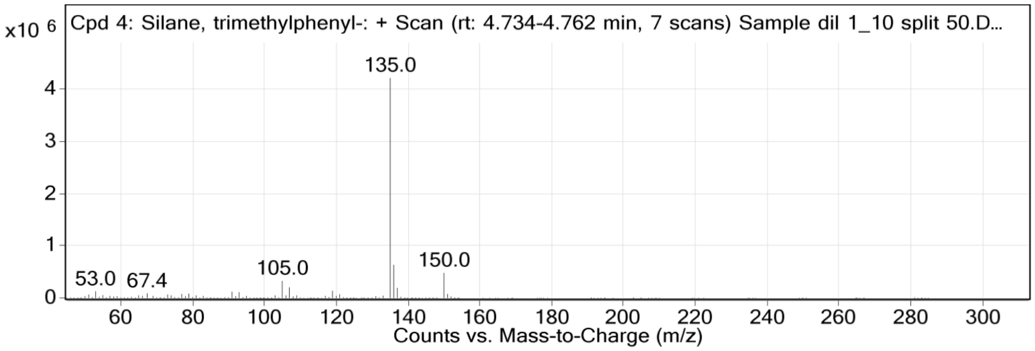
Cpd 4: Silane, trimethylphenyl-: + Scan (rt: 4.734-4.762 min, 7 scans) Sample dil 1_10 split 50.D...
x10 6
135.0
53.0
67.4
105.0
150.0
Counts vs. Mass-to-Charge (m/z)

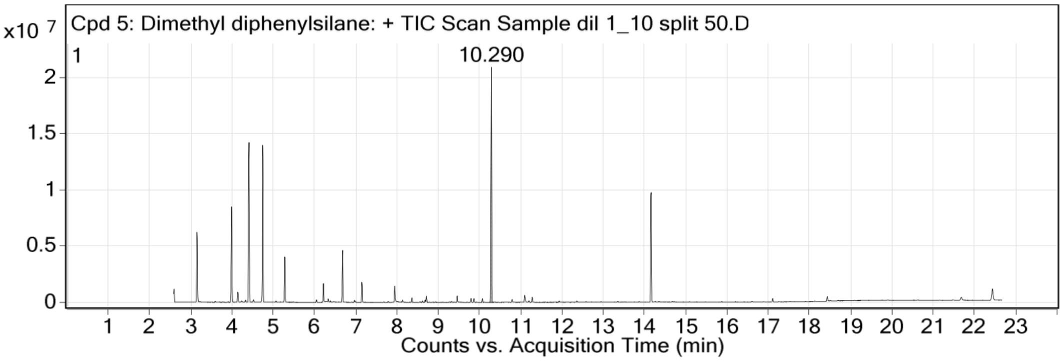
Cpd 5: Dimethyl diphenylsilane: + TIC Scan Sample dil 1_10 split 50.D
x10 7
10.290
Counts vs. Acquisition Time (min)

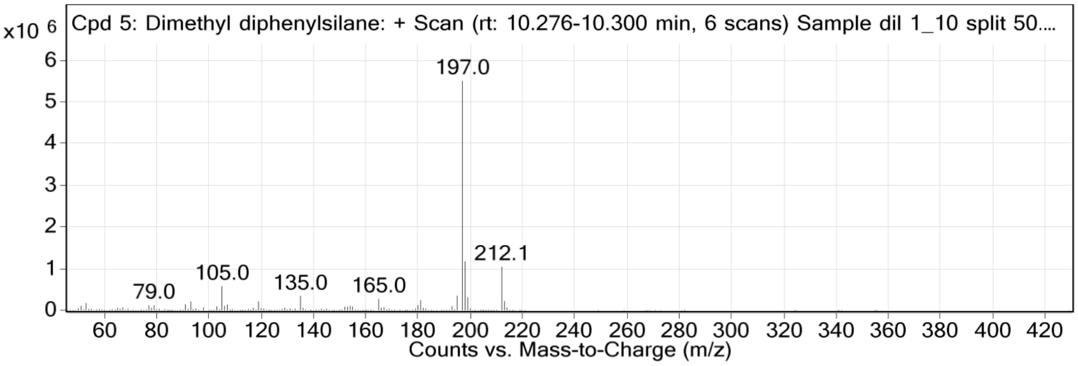
Cpd 5: Dimethyl diphenylsilane: + Scan (rt: 10.276-10.300 min, 6 scans) Sample dil 1_10 split 50....
x10 6
197.0
212.1
79.0
105.0
135.0
165.0
Counts vs. Mass-to-Charge (m/z)

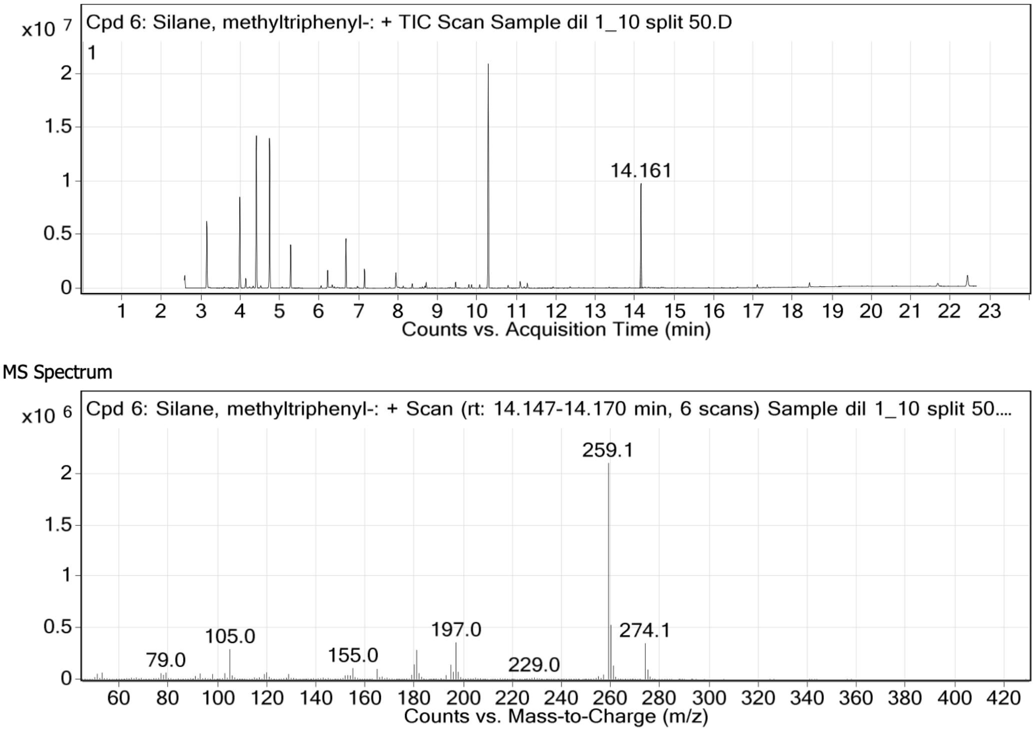

Figure S39. GC-MS data of the reaction between [**1**]$^+$[B(C_6F_5)$_4$]$^-$ and a mixture of Ph_4Si and Me_4Si.

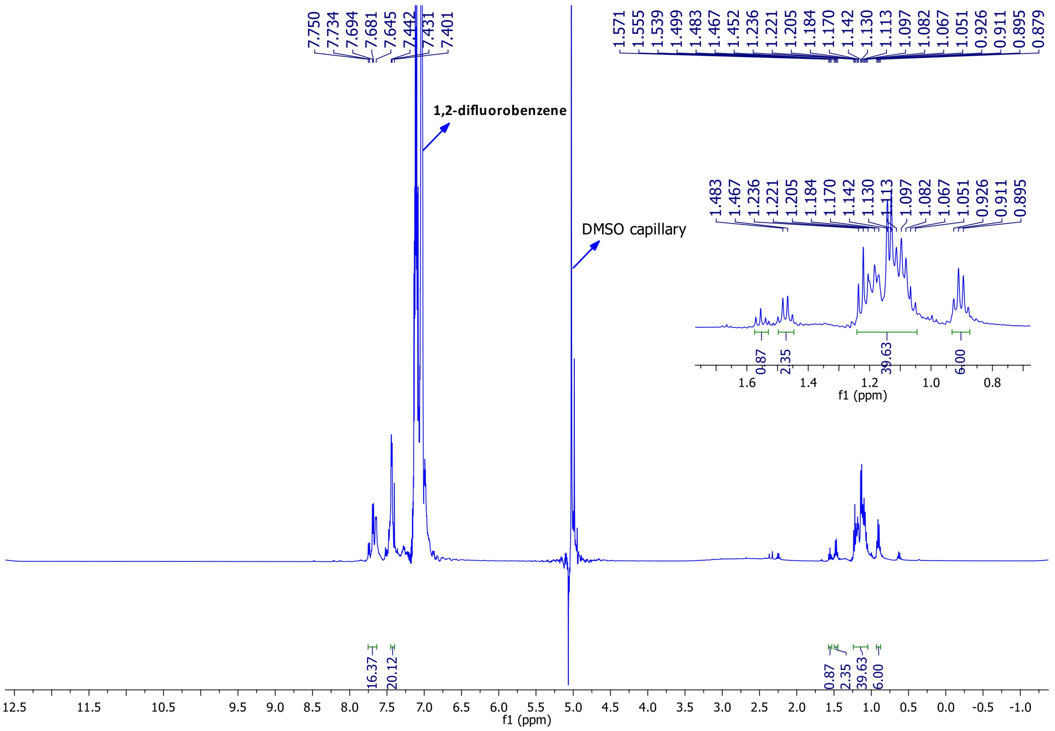

Figure S40. ^{1}H NMR spectrum (500 MHz, *o*DFB, DMSO-D$_6$ capillary) of the reaction mixture between [**1**]$^+$[B(C_6F_5)$_4$]$^-$ and a mixture of Ph_4Si and Et_4Si.

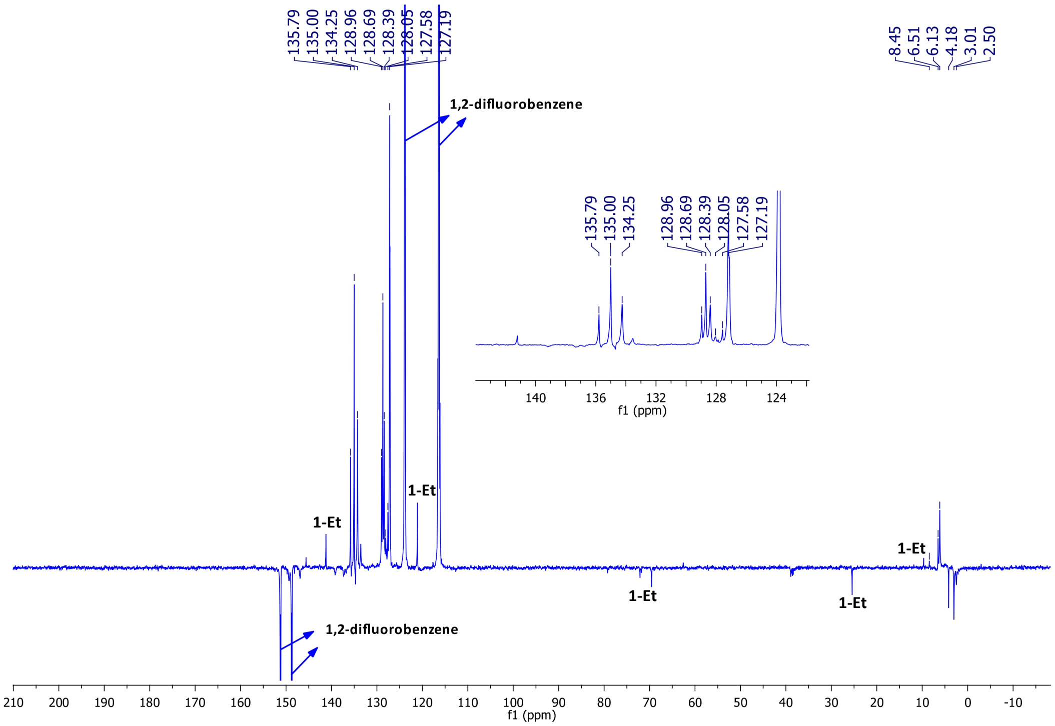

Figure S41. ^{13}C-JMOD NMR spectrum (100 MHz, *o*DFB, DMSO-D$_6$ capillary) of the reaction mixture between [**1**]$^+$[B(C$_6$F$_5$)$_4$]$^-$ and a mixture of Ph$_4$Si and Et$_4$Si.

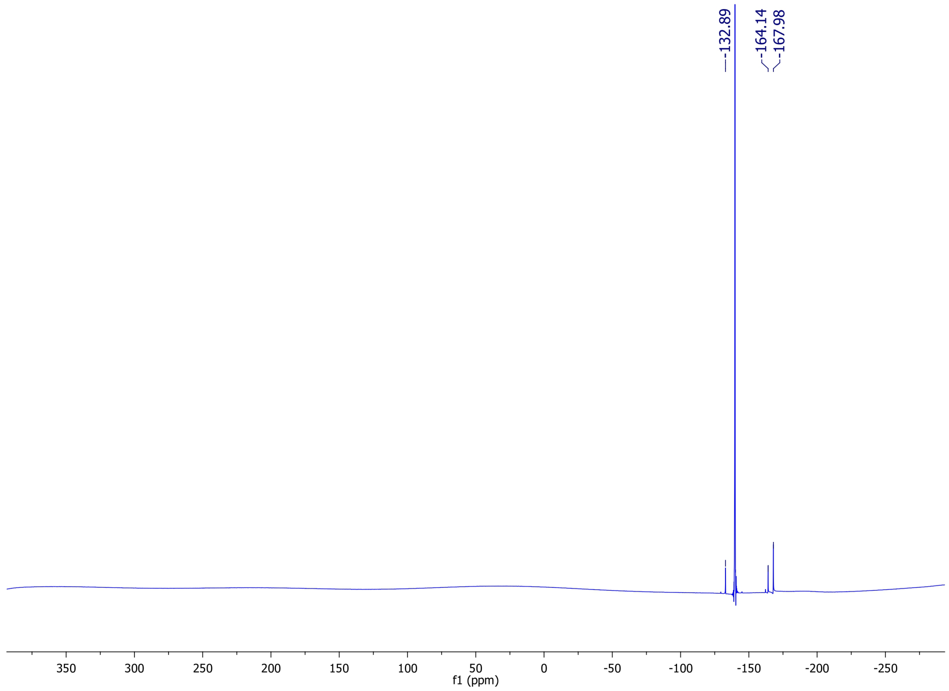

Figure S42. ^{19}F NMR spectrum (376.5 MHz, *o*DFB, DMSO-D$_6$ capillary) of the reaction mixture between [**1**]$^+$[B(C$_6$F$_5$)$_4$]$^-$ and a mixture of Ph$_4$Si and Et$_4$Si.

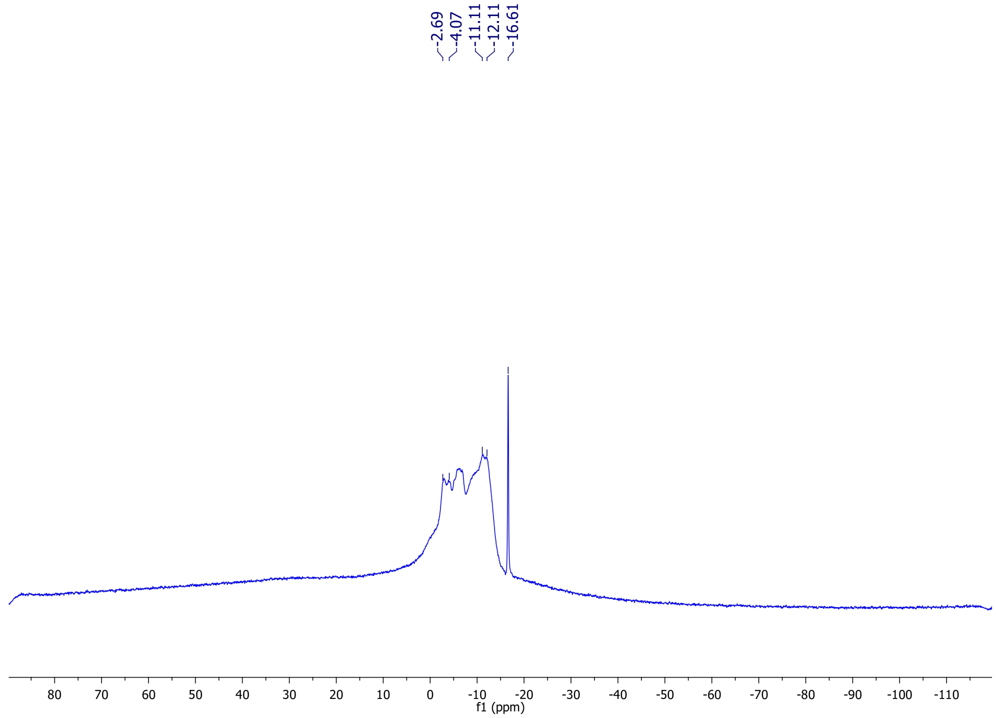

Figure S43. ^{11}B NMR spectrum (128 MHz, *o*DFB, DMSO-D$_6$ capillary) of the reaction mixture between [**1**]$^+$[B(C$_6$F$_5$)$_4$]$^-$ and a mixture of Ph$_4$Si and Et$_4$Si.

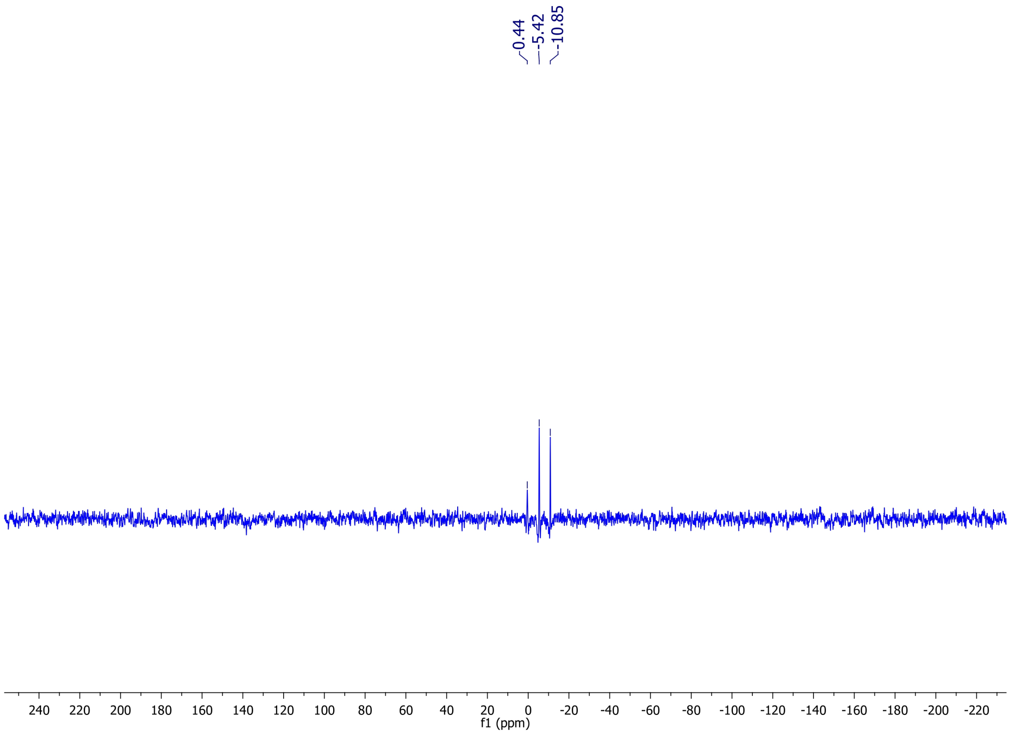

Figure S44. ^{29}Si NMR spectrum (79.5 MHz, *o*DFB, DMSO-D$_6$ capillary) of the reaction mixture between [**1**]$^+$[B(C$_6$F$_5$)$_4$]$^-$ and a mixture of Ph$_4$Si and Et$_4$Si.

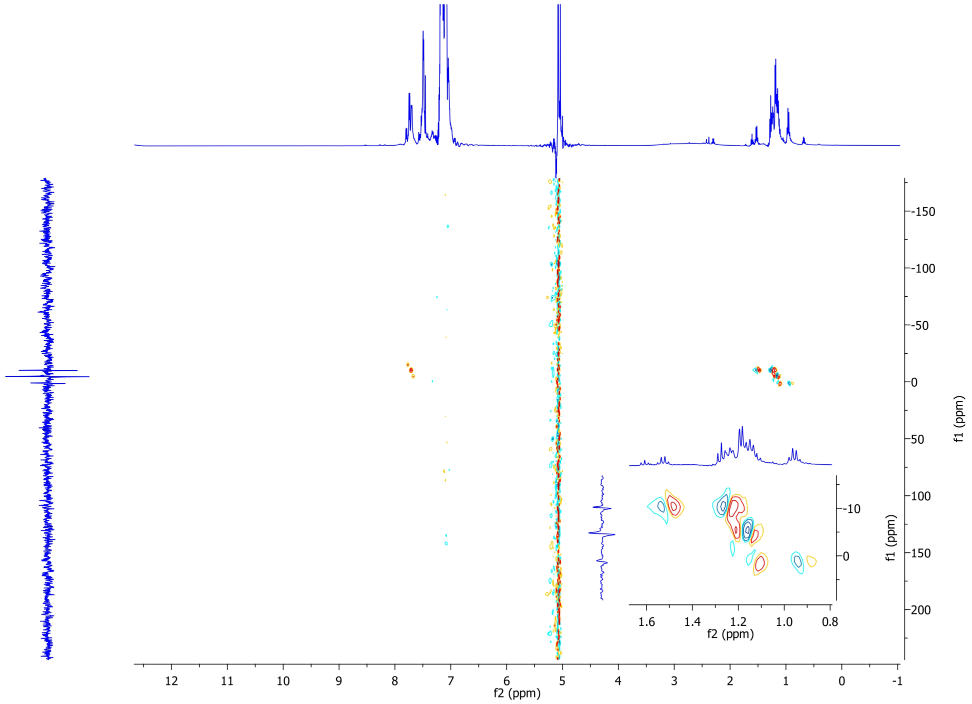

Figure S45. $^{1}H/^{29}Si$ HSQC NMR spectrum (500/79.5 MHz, *o*DFB, DMSO-D_6 capillary) of the reaction mixture between [**1**]$^{+}$[B(C_6F_5)$_4$]$^{-}$ and a mixture of Ph_4Si and Et_4Si.

GC-MS data:

Compound Table

Compound Label	RT	Name	DB Formula	Hits (DB)
Cpd 6: Silane, triethylphenyl-	14.557	Silane, triethylphenyl-	C12H20Si	3
Cpd 9: Silane, diethyldiphenyl-	19.782	Silane, diethyldiphenyl-	C16H20Si	3
Cpd 11: Silane, ethyltriphenyl	24.087	Silane, ethyltriphenyl	C21H20Si	3

Integration Peak List

Peak	RT	Height	Area	Area%	Area Sum%
6	14.557	2204999.9	2872728.35	7.33	1.82
9	19.782	11627519.04	16578223.56	42.3	10.48
11	24.087	24969346.98	39191880.25	100	24.77

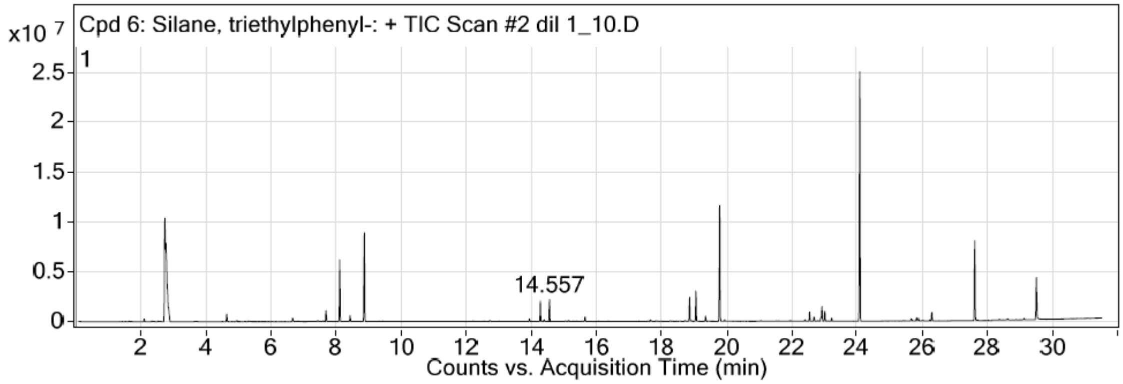
Cpd 6: Silane, triethylphenyl-: + TIC Scan #2 dil 1_10.D
x10 7
14.557
Counts vs. Acquisition Time (min)

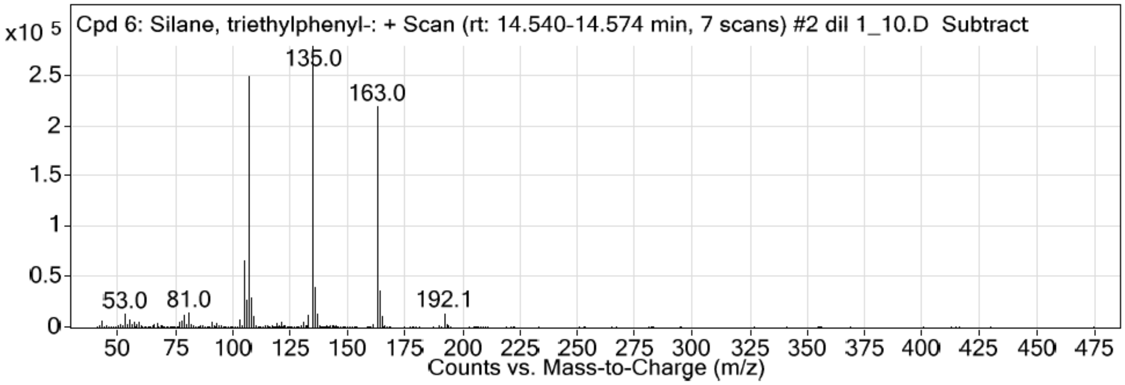
Cpd 6: Silane, triethylphenyl-: + Scan (rt: 14.540-14.574 min, 7 scans) #2 dil 1_10.D Subtract
x10 5
135.0
163.0
53.0
81.0
192.1
Counts vs. Mass-to-Charge (m/z)

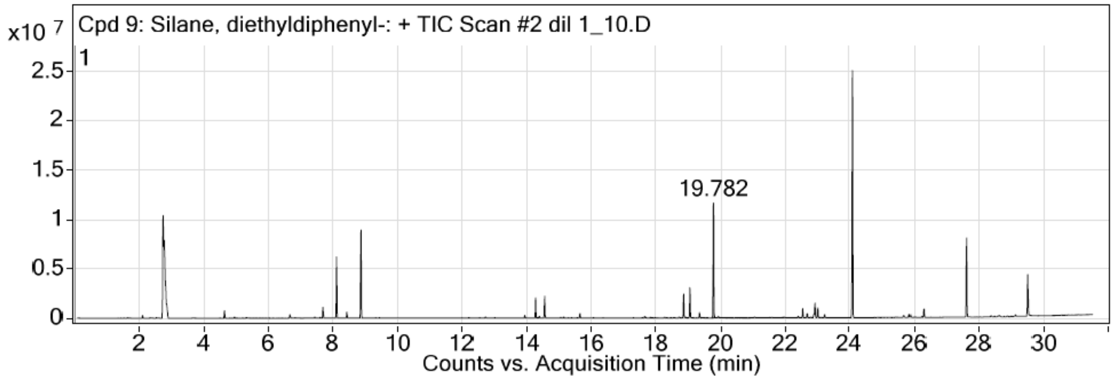
Cpd 9: Silane, diethyldiphenyl-: + TIC Scan #2 dil 1_10.D
x10 7
19.782
Counts vs. Acquisition Time (min)

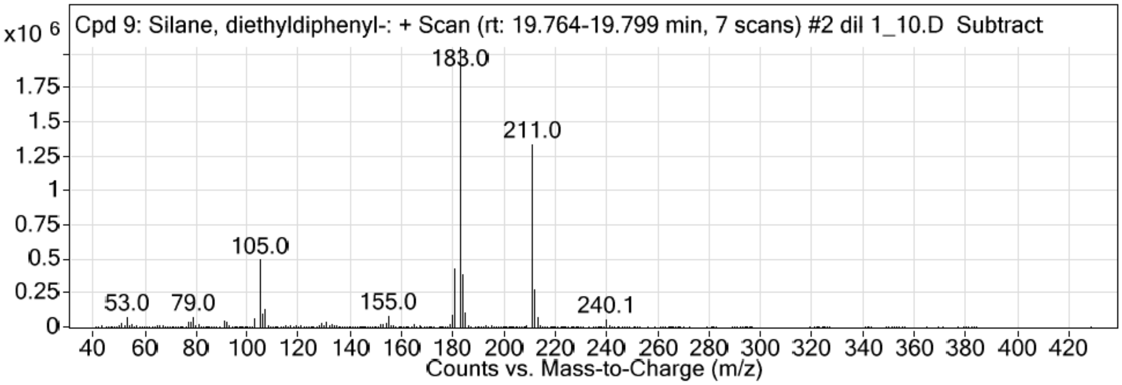
Cpd 9: Silane, diethyldiphenyl-: + Scan (rt: 19.764-19.799 min, 7 scans) #2 dil 1_10.D Subtract
x10 6
183.0
211.0
105.0
53.0
79.0
155.0
240.1
Counts vs. Mass-to-Charge (m/z)

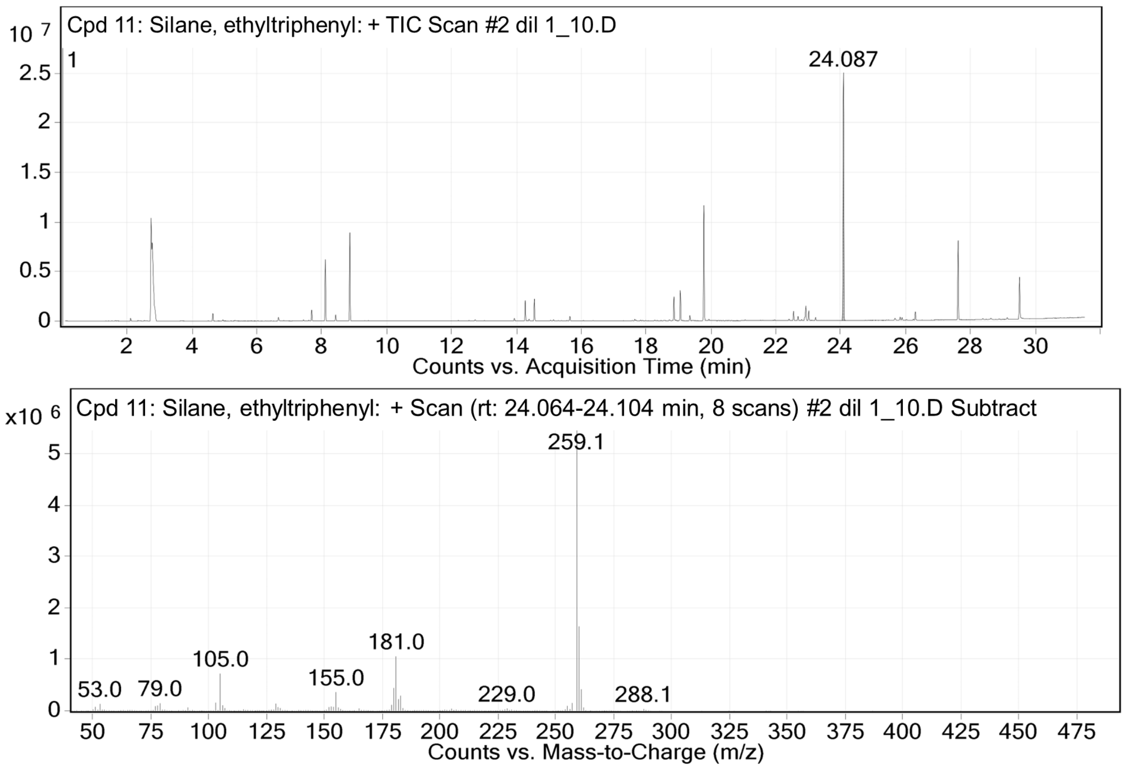

Figure S46. GC-MS data of the reaction between [**1**]$^+$[B(C_6F_5)$_4$]$^-$ and a mixture of Ph_4Si and Et_4Si.

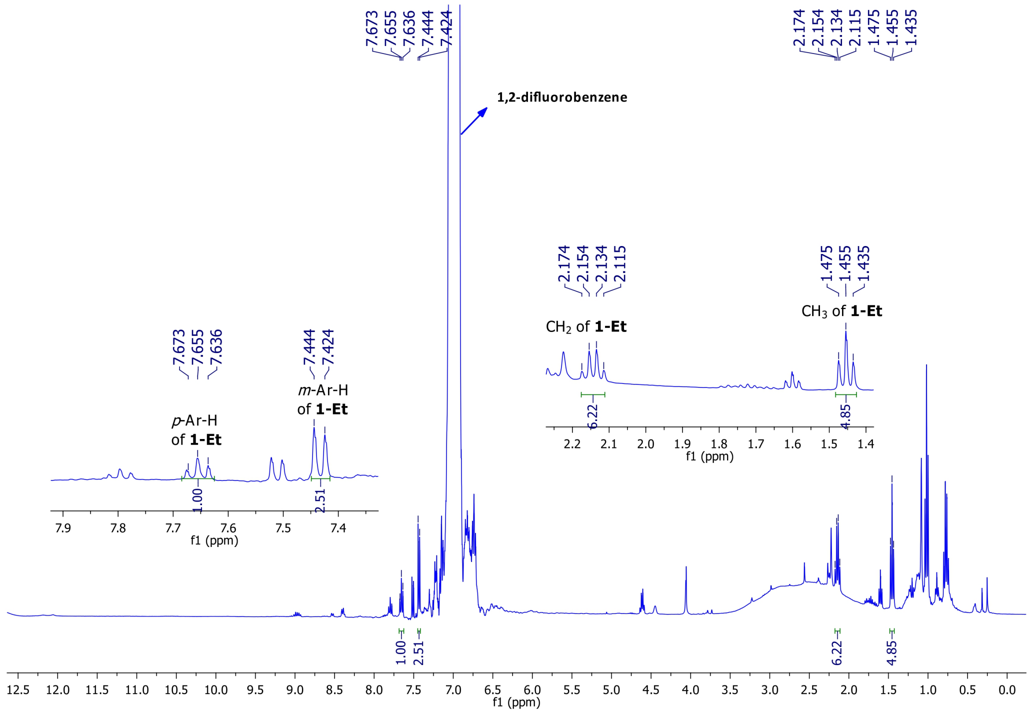

Figure S47. ^{1}H NMR spectrum (400 MHz, *o*DFB, DMSO-D$_6$ capillary) of the reaction mixture between [**1**]$^+$[B(C_6F_5)$_4$]$^-$ and Et_3SiH (1:1 ratio).

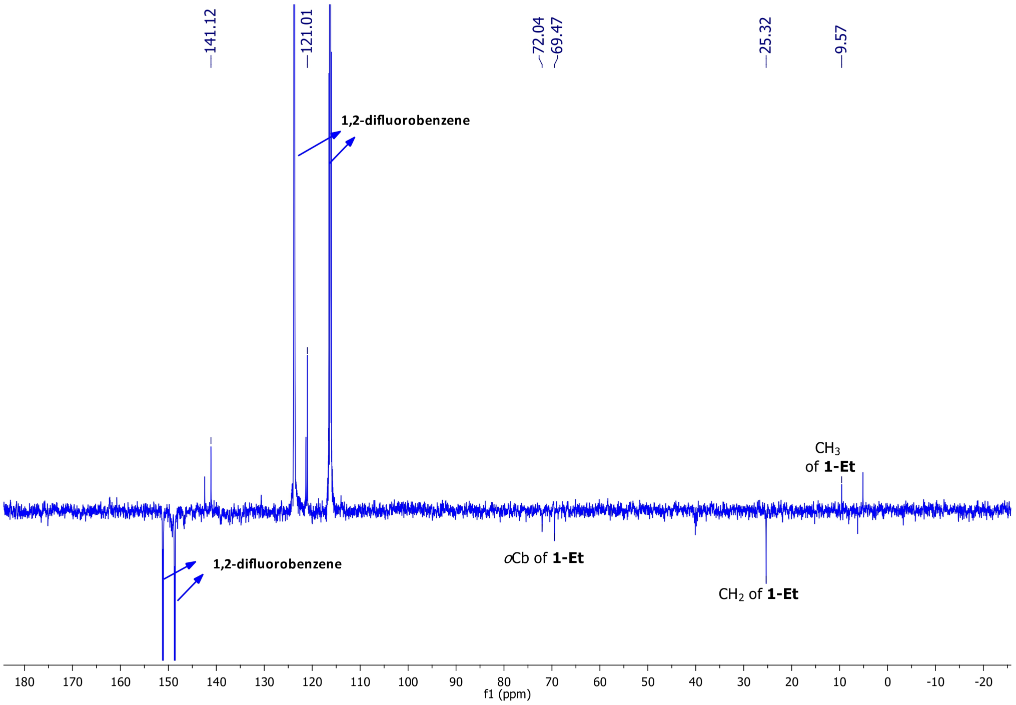

Figure S48. ^{13}C-JMOD NMR spectrum (100 MHz, *o*DFB, DMSO-D$_6$ capillary) of the reaction mixture between [**1**]$^+$[B(C$_6$F$_5$)$_4$]$^-$ and Et$_3$SiH (1:1 ratio).

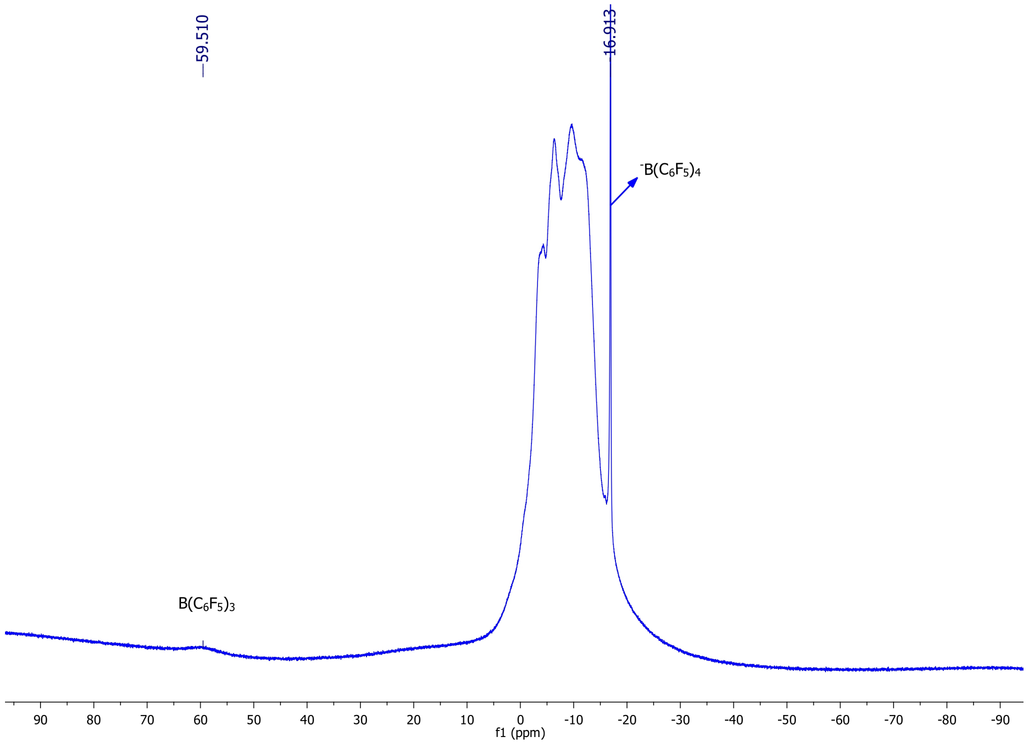

Figure S49. ^{11}B NMR spectrum (128 MHz, *o*DFB, DMSO-D$_6$ capillary) of the reaction mixture between [**1**]$^+$[B(C$_6$F$_5$)$_4$]$^-$ and Et$_3$SiH (1:1 ratio).

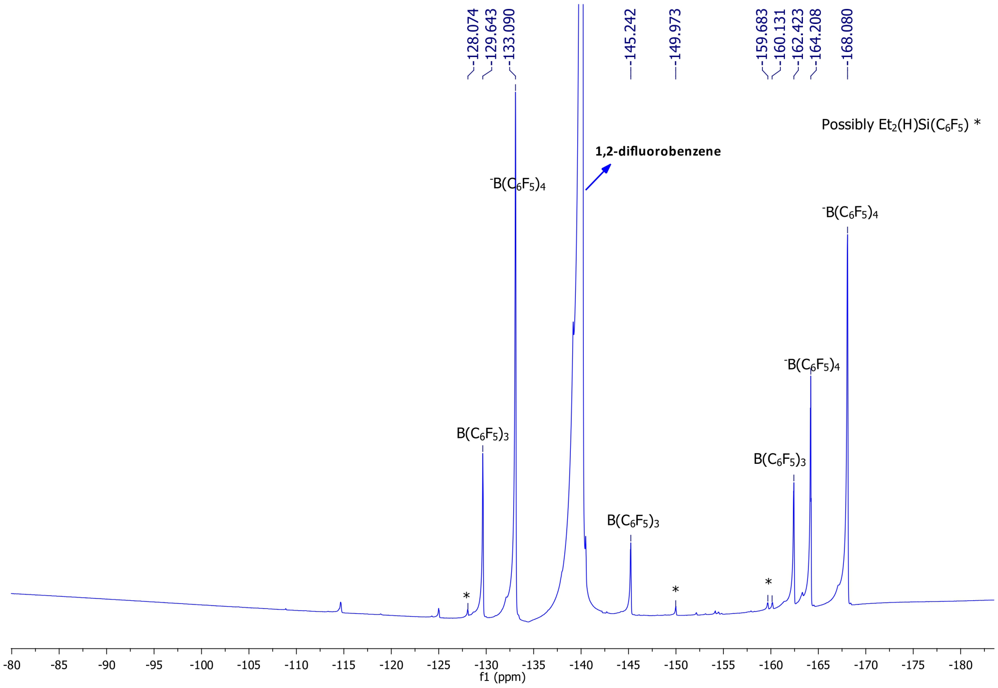

Figure S50. ^{19}F NMR spectrum (376.5 MHz, *o*DFB, DMSO-D$_6$ capillary) of the reaction mixture between [**1**]$^+$[B(C$_6$F$_5$)$_4$]$^-$ and Et$_3$SiH (1:1 ratio).

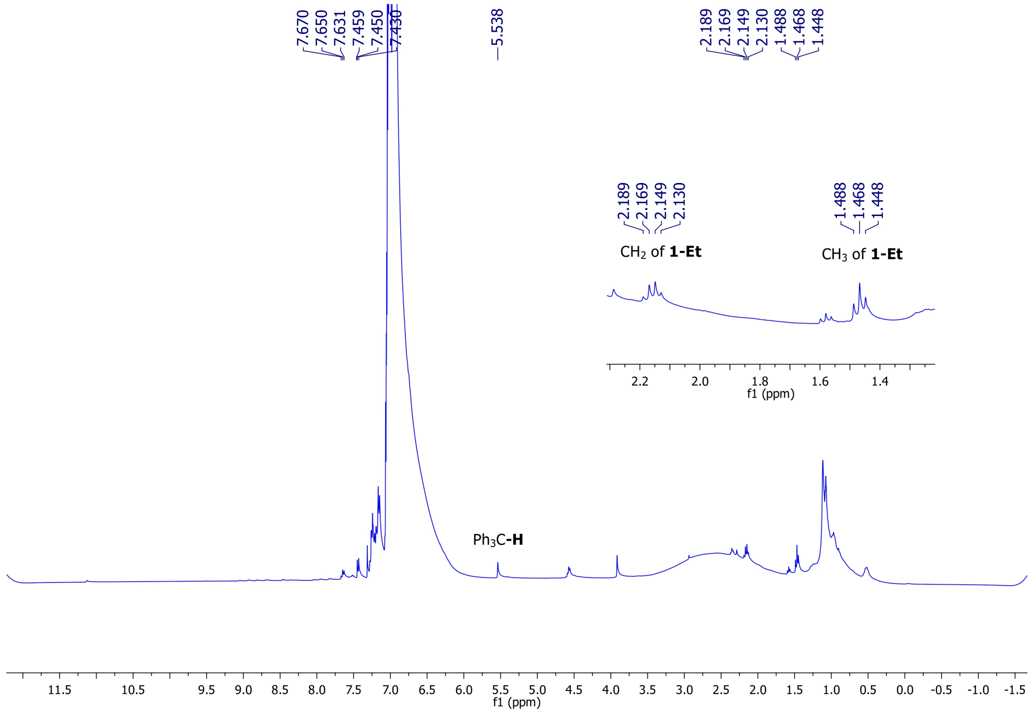

Figure S51. ^{1}H NMR spectrum (400 MHz, *o*DFB, DMSO-D$_6$ capillary) of the reaction mixture between **1-H** and [Et$_3$Si]$^+$[B(C$_6$F$_5$)$_4$]$^-$ (prepared in-situ).

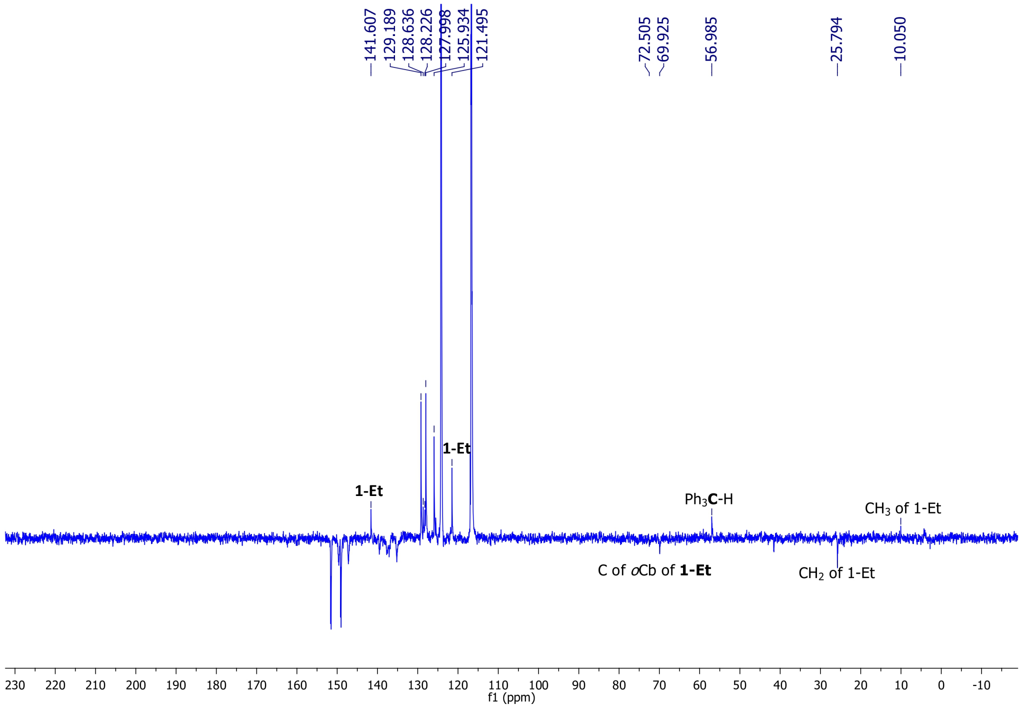

Figure S52. ^{13}C-JMOD NMR spectrum (100 MHz, *o*DFB, DMSO-D_6 capillary) of the reaction mixture between **1-H** and $[Et_3Si]^+[B(C_6F_5)_4]^-$ (prepared in-situ).

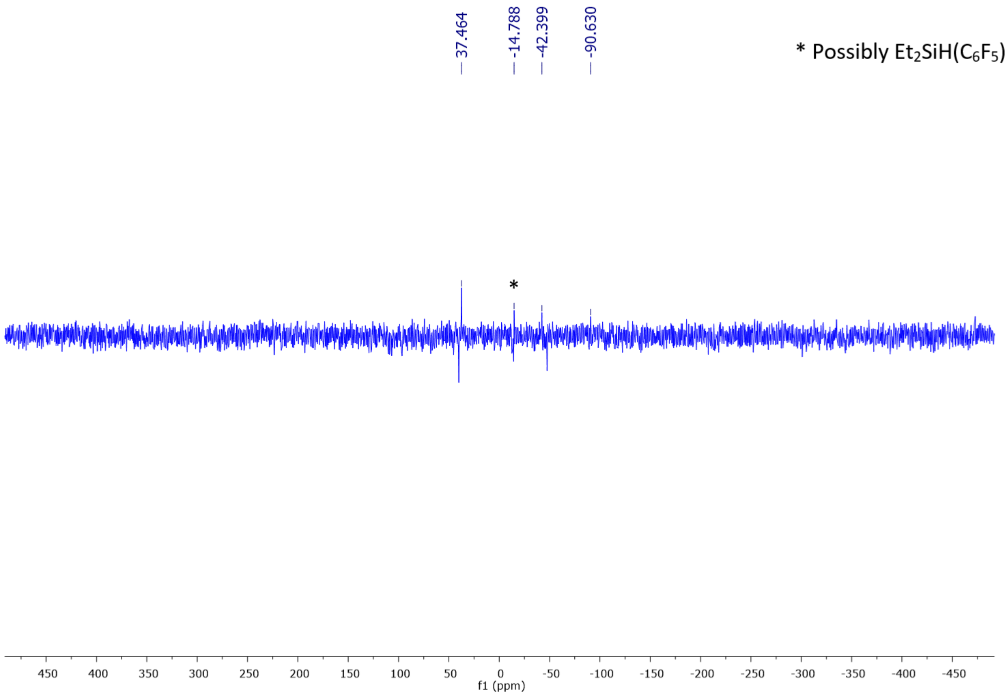

Figure S53. ^{29}Si NMR spectrum (79.5 MHz, *o*DFB, DMSO-D_6 capillary) of the reaction mixture between $[Ph_3C]^+[B(C_6F_5)_4]^-$ and Et_2SiH_2 for the generation of $[Et_2SiH]^+[B(C_6F_5)_4]^-$.

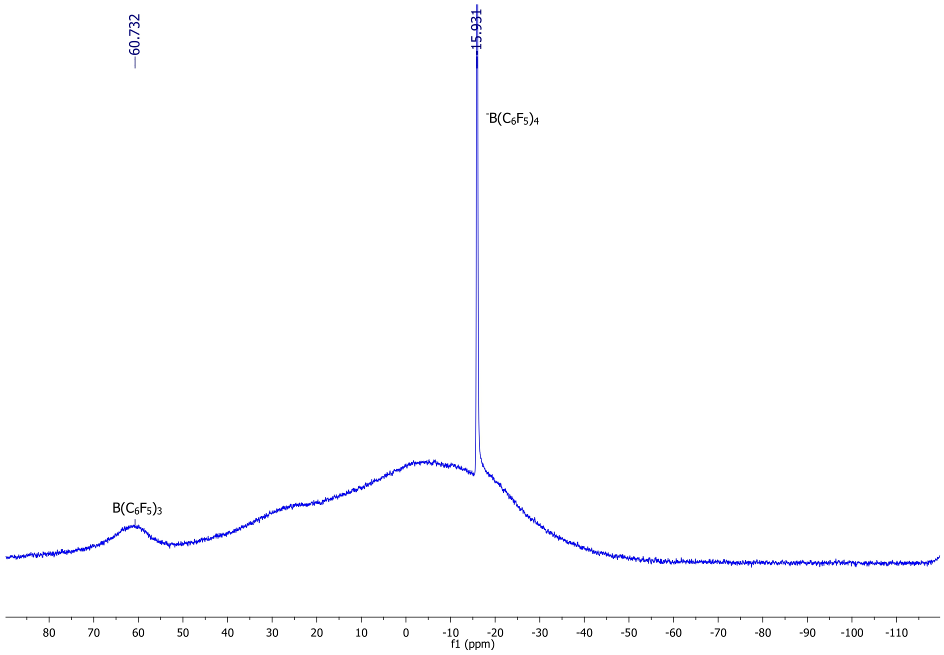

Figure S54. ^{11}B NMR spectrum (128 MHz, *o*DFB, DMSO-D$_6$ capillary) of the reaction mixture between $[Ph_3C]^+[B(C_6F_5)_4]^-$ and Et_2SiH_2 for the generation of $[Et_2SiH]^+[B(C_6F_5)_4]^-$.

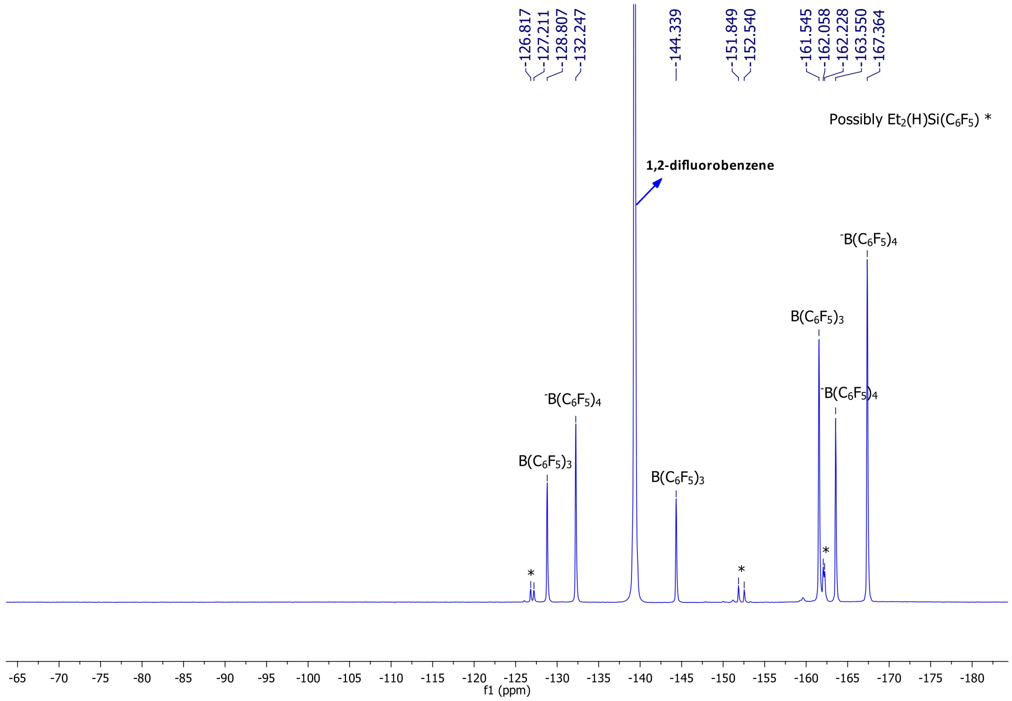

Figure S55. ^{19}F NMR spectrum (376.5 MHz, *o*DFB, DMSO-D$_6$ capillary) of the reaction mixture between $[Ph_3C]^+[B(C_6F_5)_4]^-$ and Et_2SiH_2 for the generation of $[Et_2SiH]^+[B(C_6F_5)_4]^-$. The assignment of the ^{19}F NMR chemical shifts of $Et_2(H)Si(C_6F_5)$ was made based on analogous previously reported $Me_2(H)Si(C_6F_5)$.[11]

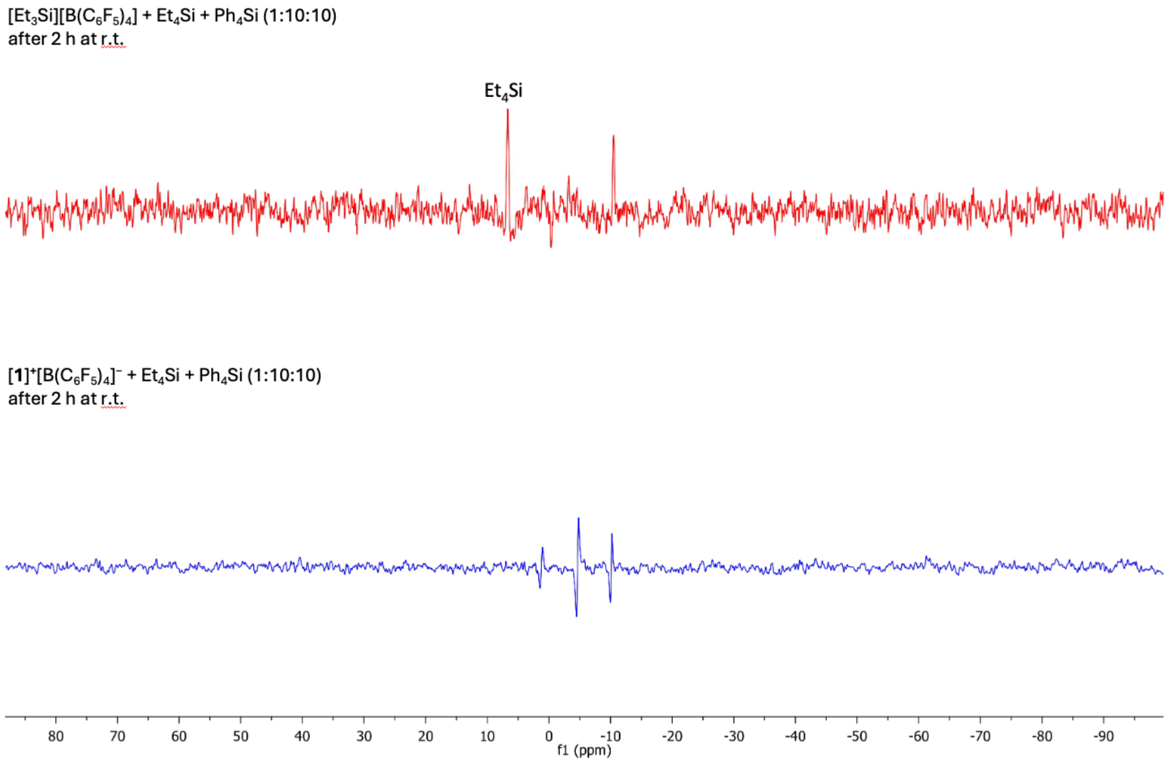

Figure S56. Stacked ^{29}Si NMR spectrum (79.5 MHz, *o*DFB, DMSO-D$_6$ capillary) showing the comparison between the reactions of $[Et_3Si]^+[B(C_6F_5)_4]^-$ and $[\mathbf{1}]^+[B(C_6F_5)_4]^-$ with Ph_4Si and Et_4Si.

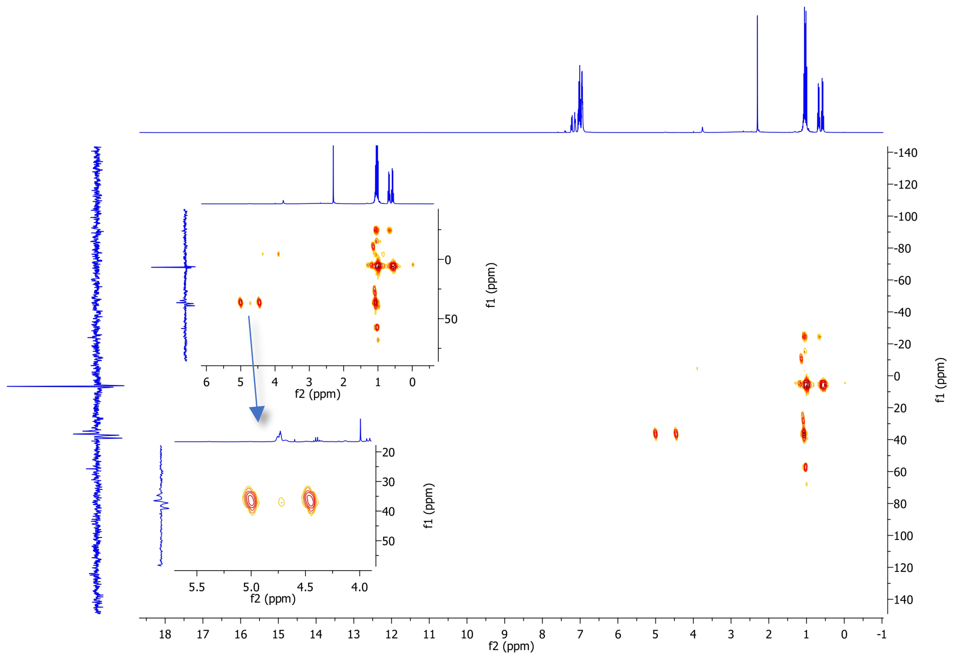

Figure S57. $^1H/^{29}Si$ HMBC NMR spectrum (400/79.5 MHz, *o*DFB) of the reaction mixture between $[\mathbf{1}]^+[B(C_6F_5)_4]^-$ and Et_3Si-H (1:10).

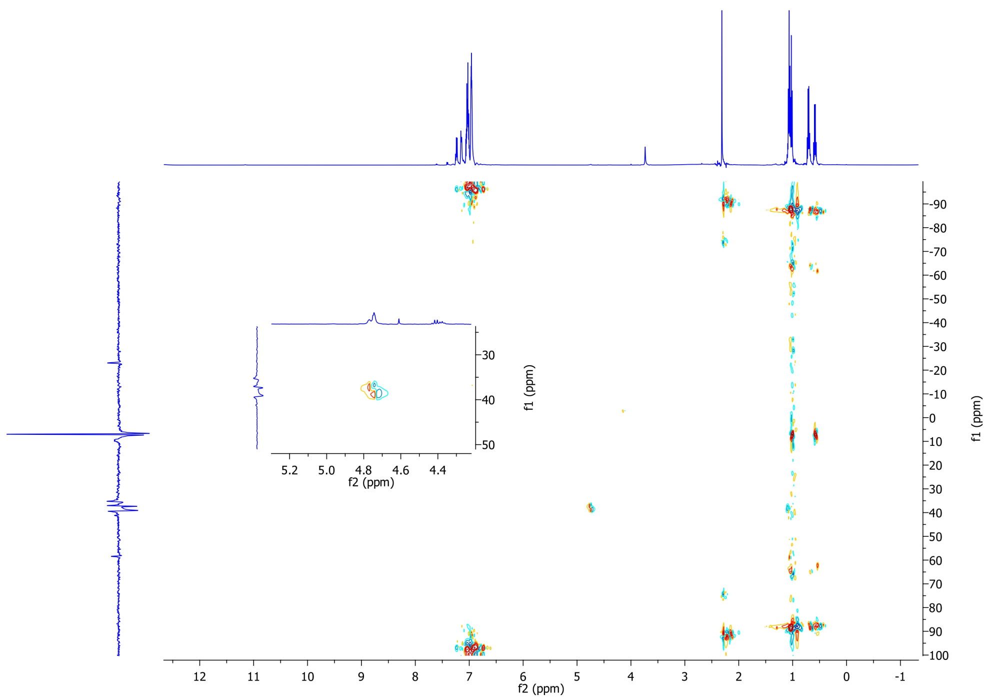

Figure S58. $^{1}H/^{29}Si$ HSQC NMR spectrum (500/79.5 MHz, *o*DFB) of the reaction mixture between $[\mathbf{1}]^{+}[B(C_6F_5)_4]^{-}$ and Et_3Si-H (1:10).

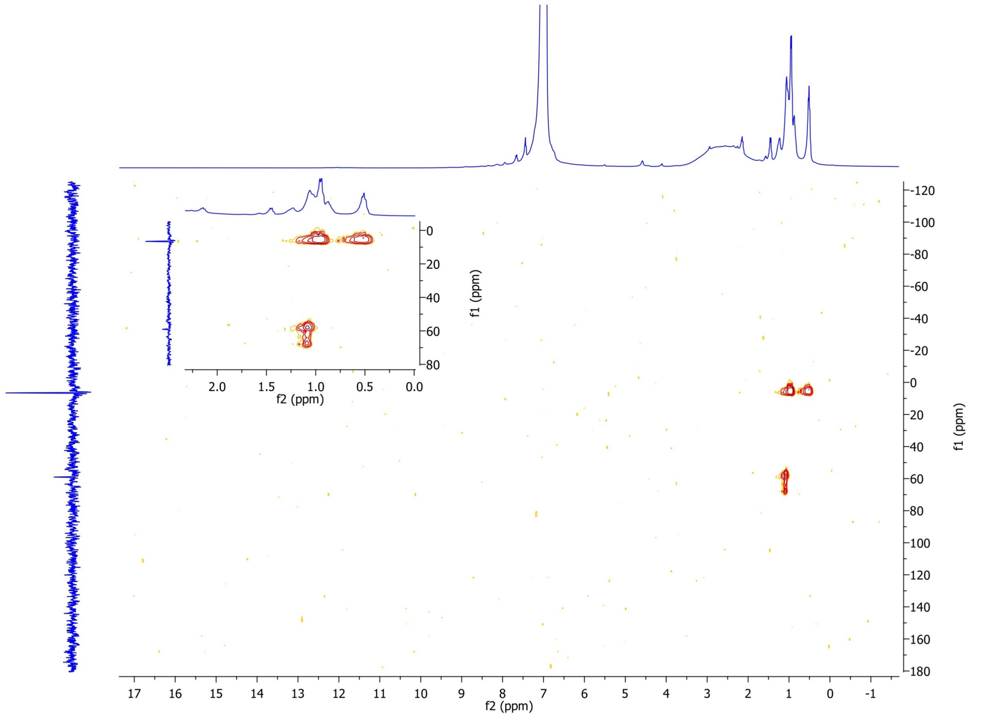

Figure S59. $^{1}H/^{29}Si$ HMBC NMR spectrum (400/79.5 MHz, *o*DFB) of the reaction mixture between $[\mathbf{1}]^{+}[B(C_6F_5)_4]^{-}$ and Et_4Si (1:2).

5. DFT calculations

DFT calculations were performed using Gaussian 09.2.[12] Geometry optimization of all the molecules was carried out using the BP86-D3 method[13,14] with Ahlrichs' def2-TZVP basis set,[15] implemented in the Gaussian 09 software. The Stuttgart basis set (SDD), along with the corresponding ECPs, was employed for the Sb atom.[16,17] Thermal energy corrections were extracted from the results of frequency analysis performed at the same level of theory. Frequency analysis of all the molecules and intermediates contained no imaginary frequency, showing that these are energy minima. The transition state geometries gave one imaginary frequency at the expected reaction coordinates, confirming that it is a first-order saddle point.

The solvent effects of *o*DFB were accounted for using the conductor-like polarizable continuum model (CPCM) through single-point corrections (recalculation of the vibrational frequencies on structures optimized in the gas phase, i.e., without re-optimization in solution).[18,19]

To support the assignment of the ^{29}Si NMR chemical shifts of the intermediates **INT^{SiEt}** and **INT1^{SiEt}** (δ(^{29}Si) = 36.5 and 58.4 ppm, respectively), the ^{29}Si NMR chemical shifts were computed using the benchmark method reported previously,[20] at the ZORA-B97-D3/def2-TZVP level of theory[15,21-23] and referenced to Me_4Si calculated at the same level. The calculated ^{29}Si chemical shift for **INT^{SiEt}** at δ = 39.8 ppm is in very good agreement with the experimentally observed value (δ = 36.5 ppm). The calculated ^{29}Si chemical shift for **INT1^{SiEt}** at δ = 82.4 ppm deviates from the experimental value (δ = 58.4 ppm) but remains within the range typically considered reasonable for computed ^{29}Si NMR chemical shifts.[20]

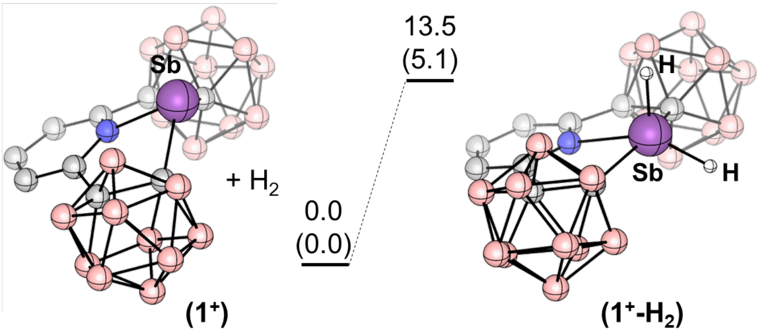

Figure S60. DFT calculated energy difference between [**1**]$^+$ and H_2 and [**1-H$_2$**]$^+$.

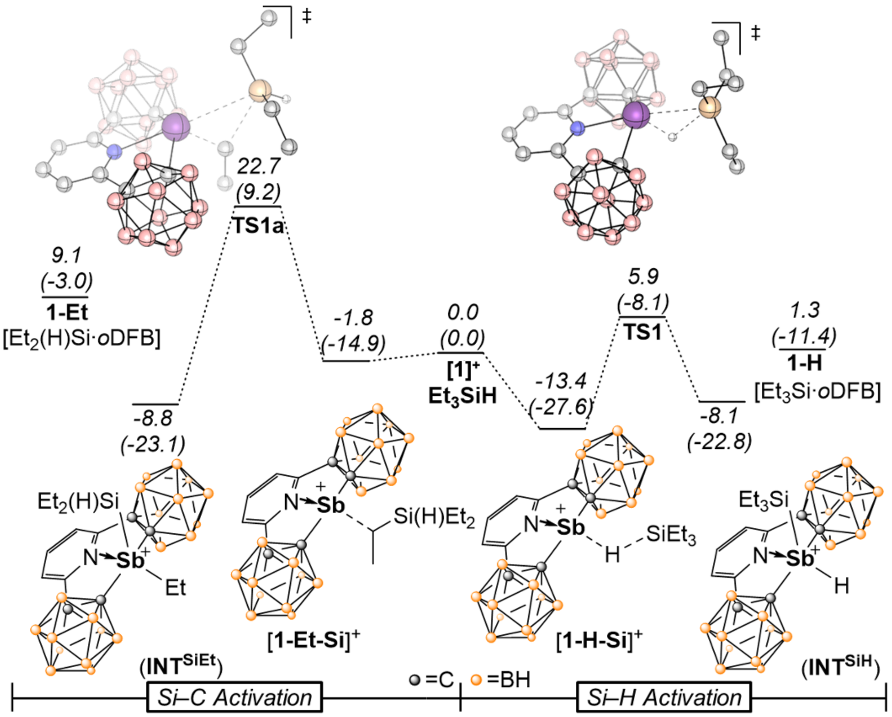

Figure S61. Gas phase DFT calculated mechanism for the activation of Et_3SiH by [**1**]$^+$, ΔG(ΔH) are given in kcal mol^{-1}.

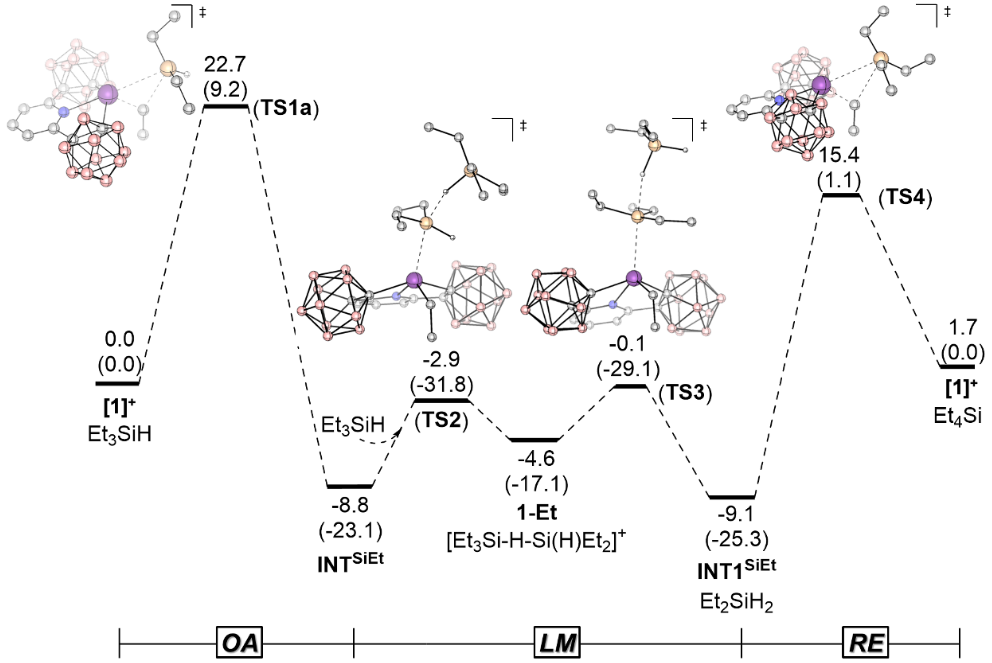

Figure S62. Gas phase DFT calculated mechanism for the redistribution of Et_3SiH catalyzed by [**1**]$^+$, ΔG(ΔH) are given in kcal mol^{-1}.

$$[Et_2(H)Si{\cdot}oDFB]^+ + [(F_5C_6)_4B]^- \xrightarrow[\text{CPCM(DFB)}]{\Delta G = \mathit{1.6},\ \Delta H = \mathit{2.6}} oDFB + [Et_2(H)Si{\cdot}(F_5C_6)_4B]$$

Figure S63. DFT calculated, including CPCM(*o*DFB) single point correction, isodesmic reaction comparing the strength of binding $[Et_2(H)Si]^+$ by *o*DFB and $[B(C_6F_5)_4]^-$, ΔG(ΔH) are given in kcal mol^{-1}.

$$[Et_2(H)Si{\cdot}HSiEt_3]^+ + oDFB \xrightarrow{\Delta G = \mathit{12.3},\ \Delta H = \mathit{11.7}} Et_3SiH + [Et_2(H)Si{\cdot}oDFB]^+$$

$$[Et_2(H)Si{\cdot}HSiEt_3]^+ + [(F_5C_6)_4B]^- \xrightarrow[\text{CPCM(DFB)}]{\Delta G = \mathit{14.3},\ \Delta H = \mathit{14.3}} Et_3SiH + [Et_2(H)Si{\cdot}(F_5C_6)_4B]$$

Figure S64. DFT calculated, including CPCM(*o*DFB) single point correction, reaction comparing the strength of binding $[Et_2(H)Si]^+$ by Et_3SiH vs. *o*DFB and $[B(C_6F_5)_4]^-$, ΔG(ΔH) are given in kcal mol^{-1}.

Computationally calculated Cartesian coordinates and energies:

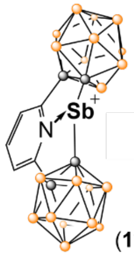

(1^+):

N	-1.04021	0.54922	0.00000
C	0.81454	-0.13636	1.91109
C	-0.79881	0.03574	2.33417
C	-1.68063	0.37957	1.18372
C	-3.07097	0.33515	1.21244
H	-3.58299	0.21563	2.16633
C	-3.76808	0.38262	0.00000
H	-4.85882	0.36590	0.00000
B	-0.11380	-1.56734	2.14121
H	-0.45182	-2.17686	1.17887
B	1.55216	-1.41036	2.76035
H	2.40307	-2.02294	2.19712
B	1.81454	0.29703	3.22330
H	2.84420	0.84071	2.96505
B	0.31989	1.20808	2.91374
H	0.26909	2.28574	2.39359

B	-1.24582	-1.15324	3.47273
H	-2.35033	-1.59072	3.39397
B	0.25214	-2.06902	3.79635
H	0.20532	-3.22675	4.07008
B	1.45308	-0.91917	4.47640
H	2.29065	-1.26026	5.25021
B	0.69406	0.70816	4.56191
H	0.97631	1.53286	5.37226
B	-0.98478	0.55514	3.94539
H	-1.91059	1.26878	4.17112
B	-0.27441	-0.76249	4.91286
H	-0.69185	-0.99441	6.00351
C	0.81454	-0.13636	-1.91109
C	-0.79881	0.03574	-2.33417
C	-1.68063	0.37957	-1.18372
C	-3.07097	0.33515	-1.21244
H	-3.58299	0.21563	-2.16633
B	0.31989	1.20808	-2.91374
H	0.26909	2.28574	-2.39359
B	1.81454	0.29703	-3.22330
H	2.84420	0.84071	-2.96505
B	1.55216	-1.41036	-2.76035
H	2.40307	-2.02294	-2.19712
B	-0.11380	-1.56734	-2.14121
H	-0.45182	-2.17686	-1.17887
B	-0.98478	0.55514	-3.94539
H	-1.91059	1.26878	-4.17112
B	0.69406	0.70816	-4.56191
H	0.97631	1.53286	-5.37226
B	1.45308	-0.91917	-4.47640
H	2.29065	-1.26026	-5.25021
B	0.25214	-2.06902	-3.79635
H	0.20532	-3.22675	-4.07008
B	-1.24582	-1.15324	-3.47273
H	-2.35033	-1.59072	-3.39397
B	-0.27441	-0.76249	-4.91286
H	-0.69185	-0.99441	-6.00351
Sb	1.11582	0.93163	0.00000

Gas Phase:

Sum of electronic and zero-point Energies= -914.008468
Sum of electronic and thermal Energies= -913.985608
Sum of electronic and thermal Enthalpies= -913.984664
Sum of electronic and thermal Free Energies= -914.055381

Single point CPCM(*o*DFB) corrected:

Sum of electronic and zero-point Energies= -914.080049
Sum of electronic and thermal Energies= -914.057184
Sum of electronic and thermal Enthalpies= -914.056240
Sum of electronic and thermal Free Energies= -914.127183

Et_3SiH:

Si	0.00164	-0.00258	0.28235
C	-0.64078	-1.68129	-0.31591
H	0.04170	-2.46300	0.05945
H	-0.55271	-1.70912	-1.41663
C	1.77618	0.28912	-0.31112
H	1.75798	0.38872	-1.41113
H	2.10544	1.27018	0.07243
C	-1.13489	1.38951	-0.31525
H	-1.20076	1.32566	-1.41612
H	-2.15263	1.18282	0.05795
H	0.00010	-0.00483	1.78717
C	2.77864	-0.80166	0.10792
H	2.51078	-1.78147	-0.31666
H	3.80109	-0.56793	-0.22855
H	2.81077	-0.91782	1.20272
C	-2.08634	-1.99479	0.11092
H	-2.79834	-1.26640	-0.30700
H	-2.40506	-2.99390	-0.22599
H	-2.19544	-1.96626	1.20652
C	-0.69483	2.80248	0.10950
H	0.29495	3.05728	-0.29971
H	-1.40120	3.57244	-0.23902
H	-0.62745	2.88949	1.20541

Gas Phase:

Sum of electronic and zero-point Energies=	-527.718749
Sum of electronic and thermal Energies=	-527.706970
Sum of electronic and thermal Enthalpies=	-527.706026
Sum of electronic and thermal Free Energies=	-527.756808

Single point CPCM(*o*DFB) corrected:

Sum of electronic and zero-point Energies=	-527.720271
Sum of electronic and thermal Energies=	-527.708417
Sum of electronic and thermal Enthalpies=	-527.707473
Sum of electronic and thermal Free Energies=	-527.758546

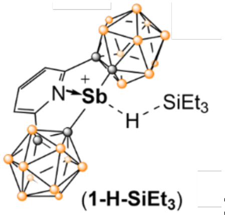

(**1-H-SiEt$_3$**):

N	0.38614	-2.10450	-0.35611
C	-1.90838	-0.59452	-0.14496
C	-1.99329	-2.23857	-0.03280
C	-0.66846	-2.92777	-0.16375
C	-0.47252	-4.29935	0.04729
H	-1.33885	-4.96081	0.18479
C	0.84733	-4.78023	0.11578

H	1.03313	-5.85495	0.26363
B	-2.09689	-1.27916	1.42405
H	-1.13937	-1.29077	2.15359
B	-3.01361	0.15853	0.91208
H	-2.70457	1.21641	1.39394
B	-3.34820	0.02780	-0.83773
H	-3.25118	0.99622	-1.55332
B	-2.66759	-1.50553	-1.43441
H	-2.04626	-1.63232	-2.46326
B	-3.13733	-2.72025	1.15390
H	-2.88488	-3.70648	1.79964
B	-3.84117	-1.18582	1.75279
H	-4.21832	-1.07887	2.89375
B	-4.62731	-0.36867	0.35052
H	-5.58832	0.34910	0.48421
B	-4.40471	-1.40216	-1.10825
H	-5.18546	-1.44193	-2.02649
B	-3.48569	-2.86101	-0.59962
H	-3.45938	-3.93902	-1.13886
B	-4.70346	-2.15489	0.50238
H	-5.72227	-2.75217	0.74798
C	2.03400	0.09667	-0.09272
C	2.66060	-1.42588	0.03092
C	1.65176	-2.52225	-0.13339
C	1.92726	-3.88038	0.07773
H	2.96295	-4.20960	0.23886
B	3.09140	-0.50095	-1.35623
H	2.57797	-0.82438	-2.40159
B	3.20682	1.16646	-0.74584
H	2.81725	2.05430	-1.46558
B	2.79676	1.16906	0.99071
H	2.14238	2.06338	1.45536
B	2.39448	-0.49344	1.48591
H	1.47168	-0.82066	2.18663
B	4.28827	-1.51280	-0.48963
H	4.63682	-2.53546	-1.02467
B	4.68661	0.17089	-0.97879
H	5.46171	0.39683	-1.87460
B	4.50943	1.21042	0.48083
H	5.17083	2.20758	0.63792
B	3.99907	0.17270	1.86347
H	4.28560	0.39463	3.01417
B	3.86382	-1.50441	1.25283
H	3.93522	-2.52267	1.89436
B	5.17076	-0.44880	0.64363
H	6.32281	-0.67485	0.92069
Sb	0.01469	0.09055	-1.07573
H	-0.32509	1.78729	-0.10652
Si	-0.67310	3.30789	0.38800
C	0.82838	4.27262	-0.21674
H	0.73574	5.28053	0.25214

H	1.74611	3.82674	0.22061
C	0.93532	4.39550	-1.74735
H	0.97424	3.40200	-2.24416
H	0.07489	4.94615	-2.18063
H	1.85700	4.93719	-2.04238
C	-2.24433	3.74680	-0.55591
H	-3.05803	3.06026	-0.24131
H	-2.08316	3.57207	-1.64202
C	-2.63522	5.22003	-0.29131
H	-1.85370	5.92804	-0.63830
H	-3.57248	5.47923	-0.82530
H	-2.81084	5.41648	0.78747
C	-0.92501	3.18002	2.25860
H	-2.02818	3.17270	2.40343
H	-0.57457	4.15725	2.66498
C	-0.27938	2.01952	3.03755
H	0.82587	2.07766	3.03361
H	-0.60732	2.02973	4.09678
H	-0.56438	1.03299	2.61676

Gas Phase:

Sum of electronic and zero-point Energies= -1441.771416
Sum of electronic and thermal Energies= -1441.735552
Sum of electronic and thermal Enthalpies= -1441.734608
Sum of electronic and thermal Free Energies= -1441.833562

Single point CPCM(*o*DFB) corrected:

Sum of electronic and zero-point Energies= -1441.831864
Sum of electronic and thermal Energies= -1441.796025
Sum of electronic and thermal Enthalpies= -1441.795080
Sum of electronic and thermal Free Energies= -1441.894323

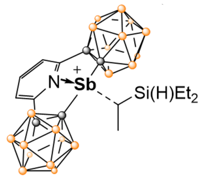

[1-Et-Si]$^+$

N	1.75818	-1.21160	-0.63072
C	1.56164	1.37955	0.26637
C	3.05577	0.72689	-0.08728
C	3.00110	-0.68976	-0.55061
C	4.11537	-1.50686	-0.71988
H	5.11382	-1.07507	-0.66784
C	3.91217	-2.87906	-0.90110
H	4.76726	-3.53953	-1.05103
B	2.45847	1.96334	-1.11976
H	2.07741	1.62023	-2.19953
B	1.57792	3.06043	-0.03593
H	0.57403	3.55345	-0.45329
B	1.64237	2.40614	1.62223
H	0.69059	2.46365	2.33078

B	2.54983	0.87464	1.58024
H	2.25839	-0.13195	2.14169
B	4.16970	1.90533	-0.61447
H	4.96678	1.56076	-1.42904
B	3.24579	3.43968	-0.56572
H	3.46300	4.31371	-1.34438
B	2.74991	3.71849	1.13738
H	2.61417	4.81545	1.58026
B	3.35221	2.35006	2.13368
H	3.65831	2.44528	3.28052
B	4.22627	1.23890	1.04479
H	5.07029	0.45443	1.34451
B	4.34555	2.99548	0.78225
H	5.37290	3.56762	0.97006
C	-0.83744	-1.69801	0.12798
C	0.17611	-2.96664	-0.23771
C	1.55432	-2.54672	-0.62353
C	2.62579	-3.41883	-0.79416
H	2.45056	-4.49363	-0.80142
B	-0.17246	-2.59211	1.43737
H	0.70303	-2.08886	2.06508
B	-1.87874	-2.08788	1.41686
H	-2.20158	-1.21214	2.15147
B	-2.44748	-2.10559	-0.27120
H	-3.15071	-1.23148	-0.67966
B	-1.11886	-2.64197	-1.31917
H	-0.82986	-2.13783	-2.36356
B	-0.08690	-4.27787	0.82409
H	0.86462	-4.91980	1.13989
B	-1.43154	-3.75398	1.87435
H	-1.50523	-4.12738	3.00271
B	-2.85473	-3.45498	0.82032
H	-3.97147	-3.60873	1.20426
B	-2.38242	-3.79027	-0.87929
H	-3.13854	-4.17543	-1.71431
B	-0.66873	-4.30947	-0.86891
H	-0.09996	-4.96167	-1.68674
B	-1.74642	-4.80956	0.45611
H	-2.05825	-5.95245	0.57702
Sb	-0.01764	0.17975	-0.74081
C	-2.00225	1.65027	0.87874
H	-1.12237	2.19053	0.47279
H	-2.17179	0.66723	0.38372
C	-1.90855	1.49775	2.39913
H	-1.78663	2.48420	2.86793
H	-2.82616	1.04676	2.80105
H	-1.06237	0.87128	2.71760
Si	-3.49221	2.65804	0.16501
H	-3.80420	3.69685	1.19642
C	-2.90513	3.43538	-1.44834
H	-2.51109	2.62533	-2.09080

H	-2.04517	4.09046	-1.22395
C	-4.93783	1.48260	-0.10176
H	-5.81884	2.09278	-0.36414
H	-4.71510	0.88452	-1.00420
C	-5.26809	0.55449	1.08205
H	-5.48546	1.12494	1.99767
H	-6.14828	-0.06864	0.86655
H	-4.43656	-0.13219	1.30688
C	-3.99239	4.22218	-2.20678
H	-3.59516	4.66207	-3.13325
H	-4.83905	3.57703	-2.48524
H	-4.39020	5.04581	-1.59517

Gas Phase:

Sum of electronic and zero-point Energies= -1441.751223
Sum of electronic and thermal Energies= -1441.715318
Sum of electronic and thermal Enthalpies= -1441.714374
Sum of electronic and thermal Free Energies= -1441.815064

Single point CPCM(*o*DFB) corrected:

Sum of electronic and zero-point Energies= -1441.813586
Sum of electronic and thermal Energies= -1441.777714
Sum of electronic and thermal Enthalpies= -1441.776770
Sum of electronic and thermal Free Energies= -1441.878744

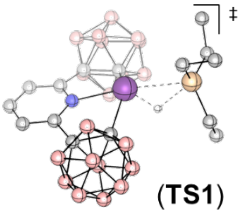

(**TS1**)
:

N	0.40322	-2.01709	-0.46893
C	-1.91523	-0.67664	0.17650
C	-1.95287	-2.27322	-0.19588
C	-0.63164	-2.85839	-0.58315
C	-0.43454	-4.20156	-0.90012
H	-1.28725	-4.87240	-0.99418
C	0.88034	-4.65361	-1.05208
H	1.06991	-5.69771	-1.30479
B	-1.99069	-1.80087	1.48369
H	-0.99955	-1.98392	2.11476
B	-2.95982	-0.31973	1.48165
H	-2.63051	0.54768	2.22866
B	-3.40442	0.05596	-0.20109
H	-3.39197	1.18631	-0.58064
B	-2.73527	-1.18554	-1.27704
H	-2.19454	-0.96770	-2.31571
B	-3.00869	-3.12954	0.85009
H	-2.69303	-4.23991	1.14145
B	-3.70358	-1.88198	1.91899
H	-4.00391	-2.13206	3.04421
B	-4.58974	-0.71979	0.87791

H	-5.54191	-0.11565	1.26397
B	-4.44165	-1.25746	-0.82996
H	-5.27079	-1.04976	-1.65970
B	-3.46308	-2.75212	-0.83822
H	-3.45413	-3.61016	-1.66394
B	-4.61695	-2.46303	0.48689
H	-5.59308	-3.13740	0.59593
C	2.11027	0.05643	0.18262
C	2.69292	-1.44958	-0.11036
C	1.66635	-2.45585	-0.52146
C	1.95348	-3.78591	-0.82643
H	2.98689	-4.12756	-0.86291
B	3.09951	-0.20597	-1.23144
H	2.55036	-0.23044	-2.28786
B	3.27869	1.23232	-0.21035
H	2.90384	2.26536	-0.66311
B	2.92692	0.80187	1.48266
H	2.29379	1.54430	2.16923
B	2.51191	-0.92126	1.54407
H	1.61559	-1.40119	2.16090
B	4.29672	-1.41702	-0.69775
H	4.60464	-2.26014	-1.48001
B	4.71552	0.31704	-0.74785
H	5.45563	0.75599	-1.57163
B	4.61598	0.94525	0.93118
H	5.29672	1.84980	1.30367
B	4.13511	-0.40092	2.01459
H	4.46359	-0.48811	3.15627
B	3.93663	-1.85613	0.99900
H	4.00093	-2.99446	1.34212
B	5.23991	-0.70167	0.63201
H	6.38128	-1.00294	0.79299
Sb	-0.02849	0.39720	-0.37843
H	-0.19471	1.27967	1.14079
Si	-0.83494	3.26509	-0.06256
C	0.82280	4.11721	0.07203
H	0.59950	5.02651	0.66514
H	1.49388	3.52415	0.71430
C	1.48788	4.48732	-1.26438
H	1.66552	3.60252	-1.89209
H	0.87314	5.19044	-1.84407
H	2.46175	4.96469	-1.08855
C	-1.69092	3.42544	-1.71888
H	-2.46152	2.65141	-1.84062
H	-0.94600	3.27625	-2.51541
C	-2.31788	4.84231	-1.80552
H	-1.58398	5.64197	-1.62075
H	-2.72248	5.00265	-2.81555
H	-3.14582	4.96689	-1.09350
C	-1.93168	3.37009	1.44027
H	-2.78161	2.68115	1.31859

H	-2.36116	4.38904	1.33957
C	-1.25554	3.21019	2.80887
H	-0.42543	3.91823	2.94557
H	-1.97853	3.38130	3.61849
H	-0.85310	2.19346	2.93924

Gas Phase:

Sum of electronic and zero-point Energies= -1441.740205
Sum of electronic and thermal Energies= -1441.704600
Sum of electronic and thermal Enthalpies= -1441.703656
Sum of electronic and thermal Free Energies= -1441.802811

Single point CPCM(*o*DFB) corrected:

Sum of electronic and zero-point Energies= -1441.800553
Sum of electronic and thermal Energies= -1441.764971
Sum of electronic and thermal Enthalpies= -1441.764027
Sum of electronic and thermal Free Energies= -1441.862911

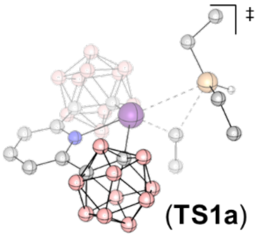

(**TS1a**)

N	0.37418	-1.95142	-0.41822
C	2.15527	0.06610	0.23440
C	2.69714	-1.42785	-0.15826
C	1.62897	-2.40997	-0.51749
C	1.89491	-3.73841	-0.84912
H	2.92430	-4.08440	-0.92806
C	0.81174	-4.59722	-1.04980
H	0.98470	-5.64162	-1.31282
B	3.02411	-0.14969	-1.26382
H	2.38864	-0.12530	-2.27118
B	3.31348	1.23952	-0.20633
H	2.91279	2.30661	-0.56134
B	3.11383	0.74486	1.48578
H	2.59711	1.47847	2.26804
B	2.67158	-0.97017	1.51337
H	1.83382	-1.47217	2.19224
B	4.24552	-1.39244	-0.89140
H	4.46933	-2.20847	-1.72940
B	4.68415	0.33610	-0.90987
H	5.35456	0.79843	-1.77992
B	4.74736	0.89410	0.79409
H	5.47073	1.77533	1.14201
B	4.34190	-0.49085	1.86114
H	4.76836	-0.62625	2.96538
B	4.03316	-1.89727	0.81103
H	4.11328	-3.04934	1.10094
B	5.31641	-0.74576	0.37511

H	6.46264	-1.06787	0.42201
C	-1.97443	-0.57731	0.08254
C	-2.00571	-2.17579	-0.27316
C	-0.66854	-2.78096	-0.55568
C	-0.49232	-4.12577	-0.88053
H	-1.35676	-4.77961	-0.98479
B	-2.23583	-1.68911	1.37313
H	-1.33467	-1.88982	2.12279
B	-3.17525	-0.19202	1.23417
H	-2.95338	0.68615	2.00745
B	-3.39723	0.17152	-0.48899
H	-3.30748	1.29142	-0.88907
B	-2.62631	-1.09237	-1.45473
H	-1.95777	-0.90449	-2.42228
B	-3.19961	-3.00632	0.63923
H	-2.94632	-4.11903	0.97888
B	-3.99354	-1.73067	1.59967
H	-4.43676	-1.95657	2.68237
B	-4.72381	-0.56576	0.44412
H	-5.70289	0.06100	0.70911
B	-4.37762	-1.12802	-1.22382
H	-5.09410	-0.91752	-2.15242
B	-3.43682	-2.63752	-1.09365
H	-3.34402	-3.50456	-1.90447
B	-4.73910	-2.31456	0.07488
H	-5.73433	-2.96959	0.07155
Sb	-0.01935	0.51986	-0.24281
C	-0.20753	1.70296	1.62150
H	0.65906	2.38833	1.57382
H	-1.15705	2.27446	1.51935
C	-0.21170	1.01552	2.99726
H	-0.25395	1.76445	3.80233
H	-1.07541	0.35015	3.10437
H	0.70391	0.42698	3.13171
Si	-0.56462	3.92081	-0.10684
H	-0.12059	4.62342	1.12664
C	0.62107	4.06439	-1.52904
H	0.26956	3.46050	-2.37790
H	1.61188	3.68578	-1.23736
C	-2.39230	3.94739	-0.40446
H	-2.48558	4.97488	-0.82851
H	-2.64445	3.26571	-1.23121
C	-3.33304	3.78345	0.79823
H	-3.04692	4.43224	1.63847
H	-4.36082	4.04174	0.50908
H	-3.34751	2.74292	1.14977
C	0.69217	5.57811	-1.88286
H	1.42262	5.71398	-2.69416
H	-0.27089	5.97196	-2.23874
H	1.03049	6.19208	-1.03466

Gas Phase:

Sum of electronic and zero-point Energies= -1441.712814
Sum of electronic and thermal Energies= -1441.676961
Sum of electronic and thermal Enthalpies= -1441.676017
Sum of electronic and thermal Free Energies= -1441.776011

Single point CPCM(*o*DFB) corrected:

Sum of electronic and zero-point Energies= -1441.777583
Sum of electronic and thermal Energies= -1441.741741
Sum of electronic and thermal Enthalpies= -1441.740797
Sum of electronic and thermal Free Energies= -1441.841602

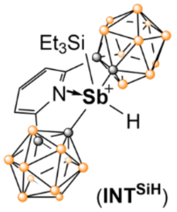

(INT^{SiH}) :

N	0.06728	-1.40078	0.82937
C	2.09487	-0.33783	-0.70738
C	2.43083	-1.36479	0.52691
C	1.24349	-1.79943	1.32668
C	1.30291	-2.67023	2.41409
H	2.26664	-2.98572	2.81149
C	0.09620	-3.14572	2.93891
H	0.10643	-3.82521	3.79228
B	2.39709	-1.99329	-1.10436
H	1.46048	-2.66884	-1.38945
B	3.06102	-0.66924	-2.07928
H	2.57423	-0.47091	-3.14853
B	3.41312	0.70473	-1.00206
H	3.18159	1.81060	-1.37405
B	2.98227	0.26164	0.66541
H	2.40516	0.95838	1.43733
B	3.64222	-2.49635	0.07797
H	3.54482	-3.59199	0.53314
B	4.09458	-2.04068	-1.58823
H	4.44800	-2.86228	-2.37445
B	4.73245	-0.36578	-1.53147
H	5.55570	0.02744	-2.29705
B	4.67162	0.21052	0.16825
H	5.43654	1.00027	0.62683
B	3.99612	-1.11701	1.15935
H	4.14331	-1.29554	2.32757
B	5.08911	-1.49767	-0.19582
H	6.17643	-1.93725	0.01182
C	-1.95999	-0.53348	-0.81534
C	-2.26936	-1.59097	0.40074
C	-1.08765	-1.92316	1.25633
C	-1.11965	-2.80303	2.33731
H	-2.06637	-3.22249	2.67483
B	-2.92947	-0.01173	0.52375

H	-2.41255	0.70819	1.31493
B	-3.32669	0.42313	-1.15458
H	-3.14142	1.54794	-1.50267
B	-2.84119	-0.91815	-2.22536
H	-2.32712	-0.67996	-3.27343
B	-2.12454	-2.20154	-1.22783
H	-1.13387	-2.80950	-1.47986
B	-3.87132	-1.45204	0.97292
H	-4.04831	-1.64745	2.13421
B	-4.59297	-0.16251	-0.03613
H	-5.42518	0.57192	0.39699
B	-4.54990	-0.73025	-1.74049
H	-5.36841	-0.38965	-2.53584
B	-3.79697	-2.35988	-1.78041
H	-4.06592	-3.19731	-2.58313
B	-3.38132	-2.79655	-0.09921
H	-3.23057	-3.88734	0.35304
B	-4.88164	-1.89359	-0.42561
H	-5.94445	-2.40619	-0.26411
Sb	0.02280	0.43485	-0.81116
H	0.06122	0.61999	-2.51279
Si	-0.12696	2.79590	0.51521
C	-1.64838	3.72822	-0.09900
H	-1.27652	4.77123	-0.12047
H	-1.84765	3.48458	-1.15465
C	-2.93459	3.65349	0.73913
H	-3.40556	2.66498	0.68744
H	-2.74814	3.88014	1.79879
H	-3.66946	4.38372	0.37141
C	-0.16062	2.36608	2.34734
H	0.59762	1.60412	2.58508
H	-1.14037	1.92355	2.58583
C	0.07490	3.63648	3.19751
H	-0.64229	4.43564	2.95513
H	-0.04193	3.40801	4.26653
H	1.08768	4.03937	3.05427
C	1.43382	3.67724	-0.04745
H	2.32460	3.10097	0.24154
H	1.45003	4.58559	0.58532
C	1.47837	4.07662	-1.53277
H	0.61489	4.69524	-1.81713
H	2.38607	4.65610	-1.75213
H	1.48949	3.19932	-2.19744

Gas Phase:
Sum of electronic and zero-point Energies= -1441.763716
Sum of electronic and thermal Energies= -1441.728006
Sum of electronic and thermal Enthalpies= -1441.727061
Sum of electronic and thermal Free Energies= -1441.825018

Single point CPCM(*o*DFB) corrected:
Sum of electronic and zero-point Energies= -1441.825431

Sum of electronic and thermal Energies= -1441.789578
Sum of electronic and thermal Enthalpies= -1441.788634
Sum of electronic and thermal Free Energies= -1441.887716

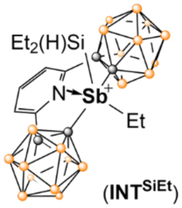

(INT^{SiEt}) :

N	0.21406	-1.31607	0.79076
C	-1.98714	-0.43740	-0.63200
C	-2.14121	-1.58412	0.53473
C	-0.88564	-1.89789	1.28161
C	-0.81340	-2.79312	2.34826
H	-1.71840	-3.26221	2.73138
C	0.44791	-3.07868	2.88026
H	0.53953	-3.76788	3.72086
B	-2.86934	-0.05033	0.81595
H	-2.34320	0.65270	1.61763
B	-3.43582	0.45791	-0.78535
H	-3.34711	1.61453	-1.05602
B	-2.98281	-0.78706	-1.97812
H	-2.59090	-0.47165	-3.05703
B	-2.11323	-2.08468	-1.13562
H	-1.11985	-2.61934	-1.51437
B	-3.69300	-1.56875	1.24928
H	-3.75775	-1.83975	2.40692
B	-4.56679	-0.26909	0.39262
H	-5.39267	0.38976	0.94315
B	-4.64796	-0.72691	-1.34096
H	-5.54747	-0.38481	-2.04266
B	-3.81716	-2.30426	-1.54935
H	-4.11197	-3.10397	-2.38119
B	-3.22932	-2.81759	0.05632
H	-2.98263	-3.92319	0.42224
B	-4.79854	-1.98532	-0.08143
H	-5.81471	-2.56682	0.13702
C	2.11043	-0.07057	-0.77324
C	2.56022	-1.10212	0.41918
C	1.43281	-1.64441	1.23636
C	1.59588	-2.52125	2.30822
H	2.59481	-2.77523	2.65980
B	2.53870	-1.68217	-1.22516
H	1.65604	-2.42549	-1.51419
B	3.07462	-0.28329	-2.17305
H	2.56849	-0.11408	-3.23646
B	3.32707	1.08924	-1.06157
H	2.97615	2.18605	-1.37448
B	2.98216	0.55922	0.59761

H	2.37378	1.18265	1.40599
B	3.84719	-2.11467	-0.08285
H	3.85282	-3.22690	0.34189
B	4.22417	-1.57784	-1.74351
H	4.62483	-2.34401	-2.56246
B	4.72494	0.14428	-1.65201
H	5.49533	0.62357	-2.42352
B	4.65599	0.66490	0.06345
H	5.36303	1.50114	0.53195
B	4.11524	-0.74182	1.02899
H	4.30098	-0.93869	2.18869
B	5.20226	-0.99229	-0.35807
H	6.32634	-1.34682	-0.18633
Sb	-0.01599	0.58689	-0.77881
C	-0.08196	1.16247	-2.88254
H	0.94672	1.49128	-3.08236
H	-0.25363	0.22910	-3.43660
C	-1.10903	2.24283	-3.21281
H	-1.04744	2.47346	-4.28789
H	-2.13901	1.92747	-3.00480
H	-0.91472	3.17862	-2.66955
Si	-0.09390	2.58736	0.93455
H	1.16258	3.28411	0.52902
C	-1.61789	3.62561	0.57359
H	-2.51989	3.00691	0.69621
H	-1.60152	3.95332	-0.47764
C	0.00259	1.91478	2.69662
H	-1.02714	1.91170	3.09092
H	0.33211	0.86420	2.69769
C	0.92519	2.77097	3.58837
H	0.60837	3.82392	3.61250
H	0.91365	2.39908	4.62318
H	1.96613	2.74615	3.23451
C	-1.66639	4.84517	1.52285
H	-2.54586	5.46549	1.29781
H	-1.74379	4.53927	2.57693
H	-0.77637	5.48363	1.41910

Gas Phase:
Sum of electronic and zero-point Energies= -1441.764250
Sum of electronic and thermal Energies= -1441.728410
Sum of electronic and thermal Enthalpies= -1441.727466
Sum of electronic and thermal Free Energies= -1441.826135

Single point CPCM(*o*DFB) corrected:
Sum of electronic and zero-point Energies= -1441.817689
Sum of electronic and thermal Energies= -1441.781933
Sum of electronic and thermal Enthalpies= -1441.780989
Sum of electronic and thermal Free Energies= -1441.879794

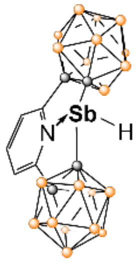

(1-H) :

N	1.07018	0.25037	0.00000
C	-0.73497	-0.05344	2.05789
C	0.87407	0.02296	2.36757
C	1.73072	0.24547	1.16162
C	3.12227	0.34334	1.21035
H	3.63671	0.34046	2.17018
C	3.81642	0.41854	0.00000
H	4.90424	0.50175	0.00000
B	-0.10210	1.32715	2.90602
H	-0.01664	2.35384	2.30814
B	-1.61700	0.54884	3.39197
H	-2.63623	1.12946	3.16860
B	-1.49568	-1.19629	3.07959
H	-2.43021	-1.79197	2.64096
B	0.09360	-1.52696	2.37612
H	0.31828	-2.25086	1.45896
B	1.21311	0.66567	3.91358
H	2.20294	1.31999	4.03072
B	-0.39125	0.99315	4.61804
H	-0.56213	1.89920	5.37499
B	-1.25874	-0.57210	4.73221
H	-2.06337	-0.78380	5.58743
B	-0.18639	-1.85977	4.09372
H	-0.20381	-2.98942	4.47710
B	1.33626	-1.08746	3.58967
H	2.41024	-1.59463	3.48654
B	0.49965	-0.50467	5.04638
H	0.97605	-0.66878	6.12819
C	-0.73497	-0.05344	-2.05789
C	0.87407	0.02296	-2.36757
C	1.73072	0.24547	-1.16162
C	3.12227	0.34334	-1.21035
H	3.63671	0.34046	-2.17018
B	0.09360	-1.52696	-2.37612
H	0.31828	-2.25086	-1.45896
B	-1.49568	-1.19629	-3.07959
H	-2.43021	-1.79197	-2.64096
B	-1.61700	0.54884	-3.39197
H	-2.63623	1.12946	-3.16860
B	-0.10210	1.32715	-2.90602
H	-0.01664	2.35384	-2.30814

B	1.33626	-1.08746	-3.58967
H	2.41024	-1.59463	-3.48654
B	-0.18639	-1.85977	-4.09372
H	-0.20381	-2.98942	-4.47710
B	-1.25874	-0.57210	-4.73221
H	-2.06337	-0.78380	-5.58743
B	-0.39125	0.99315	-4.61804
H	-0.56213	1.89920	-5.37499
B	1.21311	0.66567	-3.91358
H	2.20294	1.31999	-4.03072
B	0.49965	-0.50467	-5.04638
H	0.97605	-0.66878	-6.12819
Sb	-1.50889	0.54220	0.00000
H	-2.42403	-0.92019	0.00000

Gas Phase:

Sum of electronic and zero-point Energies= -914.856645
Sum of electronic and thermal Energies= -914.833428
Sum of electronic and thermal Enthalpies= -914.832484
Sum of electronic and thermal Free Energies= -914.904719

Single point CPCM(*o*DFB) corrected:

Sum of electronic and zero-point Energies= -914.865835
Sum of electronic and thermal Energies= -914.842632
Sum of electronic and thermal Enthalpies= -914.841688
Sum of electronic and thermal Free Energies= -914.913858

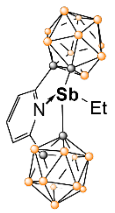

(1-Et)

:

N	-0.02964	1.24833	-0.02098
C	-2.15463	-0.54967	-0.12243
C	-2.42517	1.04742	0.06854
C	-1.19718	1.89698	0.09370
C	-1.25889	3.28241	0.25471
H	-2.22627	3.77297	0.34575
C	-0.06291	3.99834	0.30040
H	-0.07634	5.08263	0.41995
B	-2.82720	0.34684	-1.44659
H	-2.11259	0.59512	-2.36689
B	-3.43418	-1.25479	-1.02102
H	-3.16802	-2.16406	-1.74734
B	-3.32860	-1.45751	0.73779
H	-3.01254	-2.51078	1.19716
B	-2.63269	0.01086	1.43414
H	-1.80300	0.05744	2.28714
B	-3.87866	1.56620	-0.67358

H	-3.89456	2.66174	-1.14395
B	-4.57489	0.08164	-1.36547
H	-5.22283	0.11242	-2.36679
B	-4.89086	-1.04459	-0.00886
H	-5.77960	-1.84036	-0.04120
B	-4.38375	-0.24865	1.51873
H	-4.89766	-0.45452	2.57573
B	-3.75770	1.36304	1.09724
H	-3.69544	2.32368	1.80065
B	-5.15554	0.70850	0.21285
H	-6.23817	1.19429	0.34144
C	2.13421	-0.49746	-0.12970
C	2.37089	1.11071	0.02975
C	1.12253	1.92955	0.05940
C	1.15075	3.31686	0.21184
H	2.10672	3.83428	0.26955
B	2.59495	0.10916	1.41550
H	1.76121	0.16403	2.26416
B	3.31874	-1.36167	0.75900
H	3.01478	-2.40422	1.24815
B	3.42997	-1.19499	-1.00428
H	3.18403	-2.12461	-1.71194
B	2.79333	0.38551	-1.46837
H	2.07892	0.59855	-2.39760
B	3.69424	1.47467	1.05589
H	3.60899	2.44761	1.73950
B	4.34821	-0.11520	1.51549
H	4.86031	-0.28728	2.57941
B	4.87749	-0.93425	0.00928
H	5.78242	-1.71211	-0.00378
B	4.54522	0.15634	-1.37360
H	5.19740	0.17754	-2.37240
B	3.81615	1.64271	-0.71813
H	3.81352	2.72768	-1.21270
B	5.10710	0.82810	0.19237
H	6.17951	1.33766	0.31438
Sb	-0.00178	-1.27174	-0.59063
C	0.04869	-2.49027	1.23923
H	0.75821	-2.00949	1.92528
H	-0.94892	-2.43603	1.69038
C	0.44891	-3.93920	0.94035
H	0.49792	-4.52180	1.87442
H	-0.27774	-4.43794	0.28087
H	1.43812	-4.00776	0.46311

Gas Phase:

Sum of electronic and zero-point Energies= -993.464337
Sum of electronic and thermal Energies= -993.437866
Sum of electronic and thermal Enthalpies= -993.436921
Sum of electronic and thermal Free Energies= -993.516657

Single point CPCM(oDFB) corrected:

Sum of electronic and zero-point Energies= -993.473669
Sum of electronic and thermal Energies= -993.447204
Sum of electronic and thermal Enthalpies= -993.446260
Sum of electronic and thermal Free Energies= -993.525682

[Et_3Si·*o*DFB]$^+$:

C	0.70095	-0.74776	-1.64608
C	1.84490	-1.29204	-1.07490
C	2.61280	-0.50406	-0.21380
C	2.25551	0.83256	0.07615
C	1.12065	1.38250	-0.48905
H	0.11152	-1.33399	-2.35071
H	2.17346	-2.30944	-1.28941
H	0.87117	2.42362	-0.28058
C	0.28729	0.57328	-1.31633
H	-0.43416	1.07612	-1.97135
Si	-1.32615	0.09417	0.13789
C	-2.01472	1.80498	0.47035
H	-2.90153	1.60104	1.10112
H	-1.31563	2.35942	1.11721
C	-0.54266	-0.74231	1.61200
H	-0.07806	-1.68855	1.29300
H	0.25849	-0.11470	2.03053
C	-2.48844	-0.99699	-0.85282
H	-3.48824	-0.77528	-0.43388
H	-2.53445	-0.63839	-1.89465
C	-1.62693	-1.00946	2.68661
H	-2.45684	-1.61799	2.29661
H	-1.19301	-1.55638	3.53523
H	-2.05030	-0.07413	3.08035
C	-2.42923	2.63841	-0.75448
H	-2.95983	3.54942	-0.44479
H	-1.55829	2.96497	-1.34388
H	-3.10019	2.08070	-1.42468
C	-2.21531	-2.50972	-0.79126
H	-2.95720	-3.06249	-1.38443
H	-1.22203	-2.77241	-1.18483
H	-2.26717	-2.88901	0.23898
F	3.03362	1.54651	0.88992
F	3.70410	-1.00400	0.34802

Gas Phase:

Sum of electronic and zero-point Energies= -957.701137
Sum of electronic and thermal Energies= -957.682519
Sum of electronic and thermal Enthalpies= -957.681575
Sum of electronic and thermal Free Energies= -957.747764

Single point CPCM(oDFB) corrected:

Sum of electronic and zero-point Energies= -957.764973
Sum of electronic and thermal Energies= -957.747532
Sum of electronic and thermal Enthalpies= -957.746587
Sum of electronic and thermal Free Energies= -957.808836

[Et$_2$(H)Si·*o*DFB]$^+$:

C	-0.27906	-1.75191	0.12282
C	-1.22208	-1.49390	-0.86191
C	-2.15172	-0.46830	-0.65874
C	-2.15558	0.30185	0.52756
C	-1.21956	0.05482	1.51369
H	0.42514	-2.57501	0.00250
H	-1.27679	-2.07662	-1.78208
H	-1.24527	0.63974	2.43463
C	-0.21746	-0.94189	1.29610
H	0.31130	-1.33411	2.17340
Si	1.45426	0.36298	0.78256
C	0.97504	1.46417	-0.64218
H	0.74242	0.84734	-1.52473
H	0.06602	2.03212	-0.39400
C	2.90231	-0.78656	0.48967
H	3.78565	-0.18301	0.77173
H	2.87214	-1.60656	1.22630
C	2.14400	2.43411	-0.95200
H	3.06145	1.89994	-1.23958
H	1.87249	3.09167	-1.78953
H	2.38175	3.07820	-0.09262
C	3.05702	-1.31739	-0.94595
H	3.93829	-1.96965	-1.02105
H	2.18788	-1.90727	-1.27355
H	3.18915	-0.50094	-1.66964
F	-3.07588	1.25267	0.67502
F	-3.05665	-0.20286	-1.58681
H	1.52410	1.06428	2.09415

Gas Phase:

Sum of electronic and zero-point Energies= -879.080098
Sum of electronic and thermal Energies= -879.064630
Sum of electronic and thermal Enthalpies= -879.063686
Sum of electronic and thermal Free Energies= -879.123448

Single point CPCM(oDFB) corrected:

Sum of electronic and zero-point Energies= -879.146798
Sum of electronic and thermal Energies= -879.132237
Sum of electronic and thermal Enthalpies= -879.131293
Sum of electronic and thermal Free Energies= -879.187652

[$Et_3Si{\cdot}(F_5C_6)_4B$]:

F	-2.05937	1.15685	2.71350
F	-3.23410	3.52285	3.06918
F	-3.55699	5.26158	0.97833
F	-2.66735	4.55895	-1.52476
F	-1.48877	2.17444	-1.91979
F	-3.73080	0.42721	-1.37277
F	-4.69936	-1.18588	-3.26298
F	-3.23178	-3.30818	-4.19227
F	-0.72383	-3.77744	-3.15921
F	0.27621	-2.16633	-1.25012
F	0.52390	0.35660	-2.65041
F	2.90845	1.50642	-2.93379
F	4.08911	2.78291	-0.81497
F	3.08436	2.43304	1.69987
F	0.70226	1.25297	2.04306
C	-1.76382	1.51250	0.35580
F	1.35734	-1.40547	1.35698
C	-2.20217	1.93646	1.61887
F	1.10911	-3.33255	3.17734
C	-2.81222	3.17578	1.83940
F	-1.36881	-4.10025	4.09321
C	-2.97846	4.06748	0.77788
F	-3.61500	-2.86160	3.11089
C	-2.52603	3.70579	-0.49308
F	-3.39429	-0.91168	1.26637
C	-1.92547	2.46017	-0.66691
C	-1.65284	-0.76461	-1.23636
B	-1.00248	0.07923	0.01827
C	-2.92814	-0.58138	-1.78330
C	-3.46793	-1.41597	-2.77023
C	-2.72837	-2.49941	-3.24448
C	-1.45648	-2.73481	-2.71714
C	-0.96701	-1.87708	-1.73539
C	0.50378	0.69556	-0.27048
C	1.11592	0.82247	-1.54114
C	2.36242	1.39558	-1.72090
C	2.46649	1.85098	0.67124
C	1.22660	1.26526	0.80764
C	-1.01765	-1.02398	1.24444
C	0.08335	-1.70354	1.76660
C	-0.00460	-2.72506	2.71558
C	-1.25807	-3.12061	3.18042
C	-2.39784	-2.48672	2.67768
C	-2.25433	-1.47355	1.72941
C	3.16466	1.79493	-0.59112
Si	4.36723	-0.05136	-0.31455
C	4.63068	-0.21714	1.53486
H	4.93731	0.76359	1.93157
H	3.66568	-0.46203	2.00672

C	5.89985	0.47764	-1.26775
H	6.37394	-0.47958	-1.55813
H	5.57227	0.94005	-2.21308
C	5.69188	-1.28376	1.89008
H	6.65385	-1.09650	1.38971
H	5.88079	-1.28332	2.97354
H	5.36517	-2.29469	1.61328
C	6.90248	1.37716	-0.52714
H	7.77764	1.58759	-1.15943
H	6.45211	2.33932	-0.25122
H	7.26981	0.90496	0.39522
C	3.42795	-1.43394	-1.15244
H	2.39020	-1.43729	-0.79393
H	3.38564	-1.20052	-2.22989
C	4.04235	-2.83250	-0.92849
H	3.52352	-3.57186	-1.55534
H	5.11279	-2.87525	-1.18242
H	3.92591	-3.15359	0.11558

Single point CPCM(oDFB) corrected:

Sum of electronic and zero-point Energies= -3464.303973
Sum of electronic and thermal Energies= -3464.252302
Sum of electronic and thermal Enthalpies= -3464.251358
Sum of electronic and thermal Free Energies= -3464.389263

[Et_2(H)Si·$(F_5C_6)_4$B]:

F	1.91327	0.94754	-2.74215
F	3.22687	3.21531	-3.22165
F	3.67621	5.03011	-1.22085
F	2.76677	4.50857	1.31831
F	1.44185	2.22883	1.83710
F	3.56555	0.29212	1.39634
F	4.38996	-1.25861	3.40953
F	2.74221	-3.17373	4.47542
F	0.20606	-3.49933	3.46228
F	-0.65155	-1.94641	1.43057
F	-0.67463	0.66938	2.61787
F	-3.11043	1.73827	2.76111
F	-4.37138	2.66297	0.51135
F	-3.27814	2.15790	-1.94582
F	-0.81612	1.10520	-2.13986
C	1.65844	1.43642	-0.40325
F	-1.53598	-1.46972	-1.27415
C	2.10920	1.77132	-1.68948
F	-1.29732	-3.51781	-2.95996
C	2.79363	2.95692	-1.97449
F	1.17781	-4.38416	-3.78769
C	3.02592	3.88658	-0.95848
F	3.43209	-3.11857	-2.85796
C	2.56339	3.61644	0.33138

F	3.22110	-1.05050	-1.14902
C	1.88563	2.42209	0.57072
C	1.39959	-0.73439	1.32627
B	0.83247	0.05859	0.00399
C	2.68310	-0.61721	1.87046
C	3.14579	-1.41926	2.92125
C	2.31338	-2.39735	3.46577
C	1.02620	-2.56116	2.94725
C	0.61386	-1.74062	1.90022
C	-0.65361	0.73907	0.21397
C	-1.27677	0.98303	1.46423
C	-2.54729	1.51720	1.57414
C	-2.63614	1.73314	-0.85833
C	-1.36723	1.20296	-0.92186
C	0.84217	-1.12303	-1.14975
C	-0.26430	-1.81818	-1.64116
C	-0.18011	-2.90064	-2.52085
C	1.07216	-3.34560	-2.94204
C	2.21566	-2.69801	-2.46651
C	2.07701	-1.62272	-1.58864
C	-3.36384	1.73539	0.39652
Si	-4.31558	-0.16979	0.27288
H	-3.21250	-1.09927	0.61799
C	-4.94078	-0.38209	-1.47784
H	-5.58220	0.46882	-1.75586
H	-4.07844	-0.37346	-2.16215
C	-5.65461	-0.09226	1.58375
H	-5.90446	-1.15400	1.77095
H	-5.21598	0.27449	2.52513
C	-5.70813	-1.71796	-1.60244
H	-6.59395	-1.74952	-0.94980
H	-6.05625	-1.86191	-2.63571
H	-5.06928	-2.57746	-1.34980
C	-6.91719	0.70309	1.20760
H	-7.67868	0.61800	1.99642
H	-6.69395	1.76950	1.07116
H	-7.36731	0.33361	0.27417

Single point CPCM(oDFB) corrected:
Sum of electronic and zero-point Energies= -3385.686544
Sum of electronic and thermal Energies= -3385.638015
Sum of electronic and thermal Enthalpies= -3385.637071
Sum of electronic and thermal Free Energies= -3385.769400

$[Et_3Si\text{-}H\text{-}Si(H)Et_2]^+$:

Si	1.42106	-0.04469	0.19040
C	1.33489	0.45386	1.99037
H	1.06365	1.52075	2.04266
H	2.37013	0.38969	2.37493
C	2.03693	1.22486	-1.03504

H	3.11928	1.02776	-1.15015
H	1.60161	0.98865	-2.02135
C	1.88786	-1.81727	-0.16426
H	2.95087	-1.88465	0.13944
H	1.34446	-2.47694	0.53092
H	-0.21573	-0.04196	-0.06564
C	1.79453	2.69679	-0.65311
H	2.31147	2.96360	0.27959
H	2.16603	3.36852	-1.43928
H	0.72581	2.91512	-0.51118
C	0.38553	-0.40099	2.85059
H	0.65974	-1.46568	2.83216
H	0.40969	-0.07472	3.89950
H	-0.65635	-0.31455	2.50499
C	1.72061	-2.28515	-1.62154
H	2.26274	-1.63796	-2.32593
H	2.10737	-3.30593	-1.74710
H	0.66332	-2.29598	-1.92325
C	-2.04496	1.78476	-0.87427
H	-3.02153	1.86315	-1.38675
H	-1.33554	2.34463	-1.50549
C	-2.12792	2.38078	0.54421
H	-1.15707	2.33431	1.06125
H	-2.42799	3.43695	0.50390
H	-2.86367	1.85387	1.16860
C	-2.60371	-1.25780	0.01737
H	-3.52495	-1.39199	-0.57980
H	-2.92514	-0.79829	0.96628
C	-1.91576	-2.61396	0.26154
H	-1.05594	-2.50745	0.93905
H	-2.61308	-3.32374	0.72751
H	-1.55758	-3.06867	-0.67381
Si	-1.57338	-0.01995	-0.92574
H	-1.07718	-0.50881	-2.24131

Gas Phase:

Sum of electronic and zero-point Energies= -976.009220
Sum of electronic and thermal Energies= -975.988051
Sum of electronic and thermal Enthalpies= -975.987107
Sum of electronic and thermal Free Energies= -976.059665

Single point CPCM(oDFB) corrected:

Sum of electronic and zero-point Energies= -976.069284
Sum of electronic and thermal Energies= -976.050708
Sum of electronic and thermal Enthalpies= -976.049764
Sum of electronic and thermal Free Energies= -976.113818

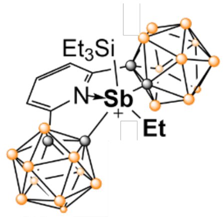

(**INT1**^{SiEt}) :

N	0.02032	-1.43242	0.87508
C	2.11965	-0.34155	-0.57600
C	2.40287	-1.39570	0.64267
C	1.18760	-1.85202	1.38165
C	1.23222	-2.73850	2.45724
H	2.19235	-3.06423	2.85443
C	0.02233	-3.20895	2.97448
H	0.02249	-3.90334	3.81576
B	2.47244	-1.98474	-0.99303
H	1.56482	-2.66465	-1.35374
B	3.19074	-0.63444	-1.88920
H	2.81244	-0.40859	-2.99225
B	3.45327	0.71501	-0.76738
H	3.23653	1.82349	-1.13865
B	2.92853	0.22987	0.85633
H	2.30829	0.90145	1.61577
B	3.65169	-2.50669	0.24981
H	3.53826	-3.61343	0.67353
B	4.19884	-2.01024	-1.37193
H	4.60664	-2.80785	-2.15686
B	4.81652	-0.33182	-1.23644
H	5.67683	0.08738	-1.94589
B	4.64618	0.20546	0.46455
H	5.37273	0.99089	0.98890
B	3.92731	-1.14917	1.38039
H	4.00135	-1.35483	2.55122
B	5.10356	-1.49088	0.08936
H	6.18038	-1.92666	0.35311
C	-2.06550	-0.42909	-0.64760
C	-2.34992	-1.51248	0.54607
C	-1.14224	-1.92643	1.32091
C	-1.18574	-2.82346	2.38765
H	-2.14199	-3.21594	2.72995
B	-2.90662	0.09474	0.77763
H	-2.29355	0.75070	1.55435
B	-3.41124	0.61116	-0.84052
H	-3.20187	1.73499	-1.17405
B	-3.10344	-0.71131	-1.98466
H	-2.70652	-0.45509	-3.07454
B	-2.37462	-2.06802	-1.10133
H	-1.44683	-2.72141	-1.45910
B	-3.89379	-1.30616	1.25913
H	-3.98510	-1.53472	2.42438

B	-4.61651	0.05485	0.35723
H	-5.36500	0.81811	0.88387
B	-4.74606	-0.44984	-1.35724
H	-5.60133	-0.03429	-2.07483
B	-4.09438	-2.11552	-1.51383
H	-4.47514	-2.90346	-2.32173
B	-3.56857	-2.63598	0.10879
H	-3.44599	-3.74950	0.51165
B	-5.03673	-1.64130	-0.06090
H	-6.11048	-2.10129	0.17225
Sb	0.00823	0.42170	-0.76598
Si	-0.07371	2.68858	0.73054
C	-1.56752	3.72118	0.19501
H	-1.13747	4.73823	0.12479
H	-1.85654	3.46887	-0.83692
C	-2.79689	3.74625	1.11680
H	-3.30203	2.77388	1.16523
H	-2.53093	4.03955	2.14292
H	-3.53175	4.47658	0.74883
C	-0.09566	2.11088	2.53270
H	0.26776	1.07455	2.61126
H	-1.14754	2.09018	2.85801
C	0.73393	3.03379	3.44985
H	0.38950	4.07775	3.40298
H	0.64998	2.70923	4.49706
H	1.80038	3.02127	3.18211
C	1.49002	3.61007	0.23542
H	2.38864	3.04385	0.51701
H	1.47536	4.49309	0.90343
C	1.55072	4.07064	-1.23047
H	0.68617	4.69332	-1.50294
H	2.45670	4.66429	-1.41702
H	1.57581	3.21962	-1.92850
C	0.05621	0.61778	-2.93848
H	-0.82582	1.23072	-3.17486
H	0.95834	1.21848	-3.12494
C	0.07320	-0.69795	-3.71696
H	-0.83017	-1.29453	-3.54774
H	0.12204	-0.47230	-4.79367
H	0.94492	-1.31577	-3.47207

Gas Phase:
Sum of electronic and zero-point Energies= -1520.378937
Sum of electronic and thermal Energies= -1520.340234
Sum of electronic and thermal Enthalpies= -1520.339290
Sum of electronic and thermal Free Energies= -1520.443266
Single point CPCM(oDFB) corrected:
Sum of electronic and zero-point Energies= -1520.437718
Sum of electronic and thermal Energies= -1520.400745
Sum of electronic and thermal Enthalpies= -1520.399801
Sum of electronic and thermal Free Energies= -1520.497869

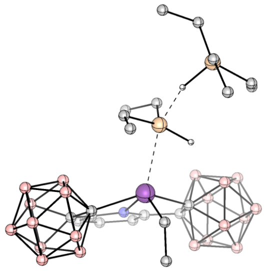
(**TS2**):

N	2.43028	0.82097	-0.78264
C	0.41603	2.12368	0.61111
C	1.41078	2.95587	-0.38980
C	2.40659	2.11692	-1.12265
C	3.29539	2.63842	-2.06257
H	3.25925	3.69671	-2.31562
C	4.22271	1.77204	-2.64580
H	4.92525	2.14615	-3.39172
B	-0.22601	2.77707	-0.86773
H	-0.42667	2.03055	-1.76794
B	-1.12748	2.86847	0.65364
H	-2.08414	2.17300	0.79517
B	0.02953	3.06226	1.98990
H	-0.15943	2.50205	3.02126
B	1.67186	3.07764	1.31608
H	2.62027	2.50581	1.74968
B	0.64392	4.31329	-1.09866
H	0.95922	4.59531	-2.21186
B	-1.00330	4.29358	-0.41542
H	-1.94066	4.67863	-1.04366
B	-0.84575	4.48102	1.36034
H	-1.68909	5.00633	2.01880
B	0.89867	4.60487	1.76778
H	1.31963	5.21760	2.69878
B	1.81145	4.50020	0.23972
H	2.90780	4.90817	0.01644
B	0.25810	5.37192	0.27569
H	0.22257	6.55487	0.13733
C	2.24081	-1.67029	0.42195
C	3.40500	-1.36649	-0.68135
C	3.35842	-0.00260	-1.28918
C	4.27226	0.43421	-2.24790
H	5.01236	-0.25708	-2.64785
B	3.83785	-1.34500	1.00028
H	4.04400	-0.27560	1.47892
B	2.85495	-2.64095	1.69984
H	2.40772	-2.49243	2.79124
B	1.84064	-3.33452	0.42043
H	0.71799	-3.63775	0.67498
B	2.17583	-2.49863	-1.10517
H	1.35800	-2.13985	-1.89119
B	4.90562	-2.07146	-0.23884
H	5.89095	-1.46938	-0.52973

B	4.56623	-2.93114	1.28448
H	5.39995	-3.04378	2.12793
B	3.32515	-4.17615	0.92914
H	3.25854	-5.19916	1.53674
B	2.90264	-4.07907	-0.81175
H	2.54498	-5.01338	-1.45958
B	3.88546	-2.77385	-1.52847
H	4.19695	-2.64250	-2.67058
B	4.59464	-3.82091	-0.27503
H	5.46613	-4.58642	-0.54794
Sb	0.58955	-0.13818	0.61419
C	0.40858	-0.35470	2.77873
H	-0.54717	0.13684	3.01275
H	0.26997	-1.43926	2.90527
C	1.54660	0.17014	3.65319
H	1.32455	-0.05425	4.70820
H	2.50742	-0.30208	3.41424
H	1.66648	1.25671	3.56793
Si	-5.33598	-0.72135	-0.28220
C	-5.94216	-1.04789	-2.03064
H	-5.50326	-0.28984	-2.70104
H	-7.03110	-0.86533	-2.04961
C	-5.99237	-1.92138	1.00827
H	-7.09419	-1.91897	0.93080
H	-5.67968	-2.93938	0.72027
H	-3.82677	-1.09104	-0.37850
C	-5.62453	-2.46754	-2.53720
H	-6.09131	-3.23926	-1.90755
H	-5.98769	-2.61775	-3.56415
H	-4.53939	-2.66032	-2.54219
C	-5.56030	-1.60385	2.45224
H	-5.96295	-0.63806	2.79052
H	-5.91692	-2.37276	3.15280
H	-4.46444	-1.55180	2.55200
C	-2.02778	-0.17480	-2.09331
H	-2.20445	0.90822	-2.02728
H	-2.94172	-0.60261	-2.53852
C	-0.82364	-0.49168	-2.99452
H	-0.64057	-1.57357	-3.06640
H	-1.00074	-0.11734	-4.01313
H	0.10060	-0.02100	-2.62810
C	-1.84914	-2.75914	-0.21239
H	-0.87273	-3.07243	-0.61182
H	-2.60355	-3.15476	-0.91389
C	-2.07603	-3.31779	1.19985
H	-3.07594	-3.06027	1.57772
H	-1.98782	-4.41348	1.20587
H	-1.33929	-2.92836	1.91873
Si	-1.97236	-0.88881	-0.34970
H	-2.32329	-0.09300	0.85908
C	-5.38342	1.07223	0.27658

H	-6.43686	1.29432	0.52664
H	-4.83863	1.13878	1.23408
C	-4.84322	2.11232	-0.72023
H	-5.37467	2.07454	-1.68230
H	-4.94820	3.13262	-0.32428
H	-3.77312	1.96014	-0.92379

Gas Phase:
Sum of electronic and zero-point Energies= -1969.496538
Sum of electronic and thermal Energies= -1969.448475
Sum of electronic and thermal Enthalpies= -1969.447530
Sum of electronic and thermal Free Energies= -1969.573658
Single point CPCM(oDFB) corrected:
Sum of electronic and zero-point Energies= -1969.553212
Sum of electronic and thermal Energies= -1969.505700
Sum of electronic and thermal Enthalpies= -1969.504756
Sum of electronic and thermal Free Energies= -1969.629612

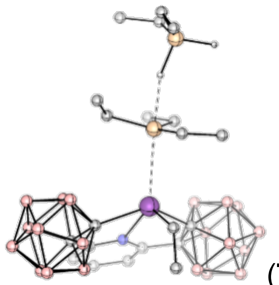

(**TS3**):

N	2.52467	0.11649	0.98085
C	1.54059	-2.03208	-0.47172
C	2.68445	-2.25005	0.68671
C	3.07708	-1.01612	1.42946
C	4.00740	-1.01448	2.46915
H	4.44635	-1.95093	2.80941
C	4.36149	0.21321	3.03325
H	5.07883	0.25019	3.85418
B	1.11779	-2.87455	0.99060
H	0.46852	-2.28722	1.79427
B	0.57116	-3.43585	-0.59920
H	-0.57575	-3.29806	-0.89141
B	1.82880	-3.08127	-1.79907
H	1.51719	-2.71526	-2.88650
B	3.17682	-2.27520	-0.97468
H	3.78007	-1.32682	-1.36203
B	2.58164	-3.79013	1.42872
H	2.85591	-3.85313	2.58581
B	1.22833	-4.59161	0.59233
H	0.52278	-5.36928	1.15648
B	1.67271	-4.72668	-1.13949
H	1.27093	-5.61290	-1.82760
B	3.29628	-3.99692	-1.36841
H	4.07127	-4.35201	-2.20076

B	3.84886	-3.42202	0.22437
H	4.96976	-3.23810	0.58136
B	2.92351	-4.93797	0.11464
H	3.44303	-5.98515	0.34557
C	1.32296	2.14333	-0.46832
C	2.37313	2.49051	0.74414
C	2.91086	1.30085	1.46987
C	3.82449	1.39621	2.51970
H	4.11560	2.37303	2.90271
B	2.93383	2.60972	-0.89007
H	3.67977	1.76018	-1.25877
B	1.53195	3.24152	-1.77196
H	1.33005	2.85615	-2.87802
B	0.17988	3.39553	-0.63101
H	-0.91437	3.09477	-0.99489
B	0.72540	2.89107	0.98302
H	0.12289	2.20740	1.75079
B	3.38575	3.81610	0.35370
H	4.50328	3.78127	0.76282
B	2.83582	4.33984	-1.25682
H	3.59319	4.81255	-2.04555
B	1.11721	4.83694	-1.10009
H	0.63053	5.67349	-1.79549
B	0.61197	4.61252	0.60693
H	-0.21805	5.27695	1.14575
B	2.02676	3.98842	1.49951
H	2.23916	4.06664	2.66892
B	2.26776	5.19542	0.21656
H	2.62923	6.29884	0.48406
Sb	0.57849	0.01705	-0.67960
C	0.66406	0.05663	-2.86493
H	0.09052	-0.82971	-3.17347
H	0.06643	0.94673	-3.11381
C	2.03323	0.10570	-3.54112
H	1.89347	0.18641	-4.63052
H	2.62635	0.97177	-3.22352
H	2.62056	-0.80026	-3.35291
Si	-5.78290	-0.86388	0.46509
C	-5.91817	-1.06943	2.32951
H	-5.11210	-1.74508	2.66254
H	-6.85842	-1.61000	2.53494
C	-7.04169	0.29129	-0.31537
H	-8.04043	-0.13523	-0.11570
H	-7.01412	1.25161	0.22663
H	-4.40611	-0.24849	0.19880
C	-5.87849	0.25333	3.11635
H	-6.71244	0.91580	2.84193
H	-5.94482	0.07428	4.19932
H	-4.94589	0.81035	2.93308
C	-6.84725	0.51585	-1.82629
H	-6.86012	-0.43237	-2.38431

H	-7.64338	1.15250	-2.23866
H	-5.89016	1.01639	-2.04163
C	-2.24658	-1.58927	1.10320
H	-1.48305	-2.28696	0.73671
H	-3.19536	-2.14434	1.02541
C	-1.96816	-1.18559	2.55961
H	-2.79101	-0.59181	2.98366
H	-1.84092	-2.07574	3.19216
H	-1.04877	-0.58461	2.64645
C	-2.68979	1.58328	0.68215
H	-1.73976	2.08302	0.90770
H	-3.15832	1.39146	1.66154
C	-3.57769	2.50062	-0.17483
H	-4.58286	2.07843	-0.31632
H	-3.69312	3.48066	0.30922
H	-3.14658	2.67742	-1.17063
Si	-2.35606	-0.11566	-0.06450
H	-5.71645	-2.17998	-0.24253
C	-2.74663	-0.34151	-1.89855
H	-3.72869	0.13980	-2.03844
H	-2.04866	0.26362	-2.49778
C	-2.78533	-1.79561	-2.39185
H	-3.10755	-1.83905	-3.44202
H	-1.80099	-2.28225	-2.32899
H	-3.48856	-2.40255	-1.80295

Gas Phase:
Sum of electronic and zero-point Energies= -1969.492168
Sum of electronic and thermal Energies= -1969.443984
Sum of electronic and thermal Enthalpies= -1969.443040
Sum of electronic and thermal Free Energies= -1969.569111
Single point CPCM(oDFB) corrected:
Sum of electronic and zero-point Energies= -1969.549069
Sum of electronic and thermal Energies= -1969.500667
Sum of electronic and thermal Enthalpies= -1969.499723
Sum of electronic and thermal Free Energies= -1969.626998

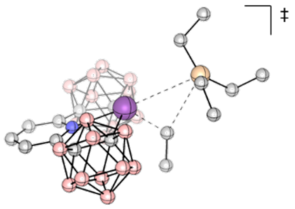

(**TS4**):

N	2.29132	0.31750	-0.35563
C	0.29323	2.14202	0.21905
C	1.81135	2.65236	-0.11173
C	2.78354	1.56122	-0.43038
C	4.13088	1.79607	-0.70409
H	4.50507	2.81698	-0.76182
C	4.97056	0.69297	-0.87541

H	6.02914	0.84154	-1.09274
B	0.58838	3.00428	-1.27037
H	0.59241	2.36871	-2.27796
B	-0.83613	3.32373	-0.27055
H	-1.89878	2.94416	-0.66460
B	-0.41556	3.11748	1.43963
H	-1.19358	2.61921	2.19019
B	1.28655	2.64018	1.53798
H	1.74510	1.79647	2.23970
B	1.83953	4.19977	-0.84816
H	2.69518	4.40500	-1.65089
B	0.12220	4.67541	-0.93955
H	-0.29002	5.35375	-1.82869
B	-0.50387	4.75262	0.74053
H	-1.38329	5.49550	1.05128
B	0.82766	4.32159	1.86363
H	0.92616	4.74833	2.97189
B	2.26859	3.98166	0.87324
H	3.40909	4.04085	1.21057
B	1.16317	5.28766	0.38832
H	1.50717	6.42714	0.44687
C	0.84290	-1.99982	0.08287
C	2.45181	-2.06885	-0.21528
C	3.09965	-0.74474	-0.46641
C	4.46067	-0.59991	-0.73528
H	5.09663	-1.47989	-0.81805
B	1.90117	-2.29385	1.41040
H	2.09714	-1.40267	2.17320
B	0.39008	-3.19891	1.21572
H	-0.51073	-2.96355	1.95860
B	0.08275	-3.40552	-0.51953
H	-1.02306	-3.29216	-0.95405
B	1.39610	-2.66072	-1.43721
H	1.25837	-1.98620	-2.40917
B	3.22172	-3.28371	0.71834
H	4.32754	-3.05899	1.09867
B	1.89769	-4.05315	1.63079
H	2.07662	-4.50550	2.71866
B	0.75768	-4.75285	0.43300
H	0.10075	-5.71925	0.67087
B	1.38491	-4.41300	-1.21304
H	1.19184	-5.12146	-2.15184
B	2.90977	-3.50712	-1.02643
H	3.80743	-3.43100	-1.80527
B	2.51736	-4.80553	0.12386
H	3.15040	-5.81501	0.13861
Sb	-0.19788	-0.02060	-0.29637
C	-1.45357	-0.19230	1.51594
H	-2.13277	0.67511	1.42814
H	-2.02820	-1.12812	1.37724
C	-0.80844	-0.19260	2.90731

H	-1.57953	-0.24155	3.69199
H	-0.14345	-1.05410	3.03655
H	-0.22588	0.72187	3.07037
Si	-3.83294	-0.42629	-0.41951
C	-3.65345	0.71130	-1.87905
H	-3.06663	0.19931	-2.65713
H	-3.08089	1.60383	-1.57988
C	-3.72231	-2.25133	-0.71968
H	-4.65835	-2.41252	-1.30360
H	-2.90967	-2.44508	-1.43766
C	-3.66983	-3.20442	0.48408
H	-4.44671	-2.97840	1.22826
H	-3.81443	-4.24154	0.15284
H	-2.69134	-3.15833	0.98177
C	-5.05407	1.13632	-2.40492
H	-4.92255	1.77009	-3.29339
H	-5.67295	0.27729	-2.70306
H	-5.60922	1.72396	-1.66046
C	-4.84708	0.15125	1.02711
H	-4.45789	-0.29626	1.95419
H	-4.75829	1.24413	1.12830
C	-6.32998	-0.26976	0.81183
H	-6.92973	0.07585	1.66566
H	-6.76479	0.17212	-0.09582
H	-6.44327	-1.36182	0.75028

Gas Phase:

Sum of electronic and zero-point Energies= -1520.337273
Sum of electronic and thermal Energies= -1520.298227
Sum of electronic and thermal Enthalpies= -1520.297282
Sum of electronic and thermal Free Energies= -1520.404301

Single point CPCM(oDFB) corrected:

Sum of electronic and zero-point Energies= -1520.401318
Sum of electronic and thermal Energies= -1520.362577
Sum of electronic and thermal Enthalpies= -1520.361633
Sum of electronic and thermal Free Energies= -1520.467430

Et_2SiH_2:

Si	0.03392	-0.78044	0.15097
C	1.22981	0.61388	-0.30042
H	0.87703	1.53956	0.18616
H	1.14832	0.79384	-1.38667
C	-1.73654	-0.38841	-0.38950
H	-1.75018	-0.31404	-1.49079
H	-2.37071	-1.25566	-0.13859
H	0.06655	-0.98954	1.63692
C	-2.31525	0.89384	0.23769
H	-1.73044	1.78269	-0.04561
H	-3.35377	1.06992	-0.08355
H	-2.31517	0.83891	1.33767

C	2.69380	0.33573	0.08613
H	3.07609	-0.56874	-0.41201
H	3.35434	1.17124	-0.19419
H	2.80060	0.18109	1.17114
H	0.49152	-2.05330	-0.49749

Sum of electronic and zero-point Energies= -449.107187
Sum of electronic and thermal Energies= -449.098647
Sum of electronic and thermal Enthalpies= -449.097703
Sum of electronic and thermal Free Energies= -449.140210

Single point CPCM(oDFB) corrected:

Sum of electronic and zero-point Energies= -449.108581
Sum of electronic and thermal Energies= -449.099983
Sum of electronic and thermal Enthalpies= -449.099039
Sum of electronic and thermal Free Energies= -449.141746

Et_4Si:

Si	0.19509	-0.01195	-0.16975
C	2.00713	0.54743	-0.22982
H	2.04265	1.61169	0.06155
H	2.34582	0.51174	-1.28041
C	-0.86178	1.15124	-1.23153
H	-0.54270	1.04499	-2.28321
H	-1.90555	0.79305	-1.19969
C	0.08872	-1.77458	-0.86311
H	0.45103	-1.72868	-1.90620
H	0.82285	-2.39873	-0.32405
C	-0.80170	2.62983	-0.80571
H	0.21752	3.03510	-0.89975
H	-1.46334	3.26235	-1.41847
H	-1.10550	2.75985	0.24512
C	2.96049	-0.26814	0.66297
H	2.97757	-1.33036	0.37320
H	3.99624	0.10239	0.60452
H	2.65640	-0.22401	1.72053
C	-1.29638	-2.44586	-0.81877
H	-2.06410	-1.83105	-1.31428
H	-1.29092	-3.42777	-1.31830
H	-1.62940	-2.61120	0.21706
C	-0.39800	0.06972	1.63503
H	0.05544	0.96872	2.08958
H	0.03934	-0.78546	2.17950
C	-1.92394	0.10264	1.84180
H	-2.19008	0.15134	2.90976
H	-2.37771	0.97814	1.35224
H	-2.41397	-0.78848	1.42254

Gas Phase:

Sum of electronic and zero-point Energies= -606.329901
Sum of electronic and thermal Energies= -606.315243
Sum of electronic and thermal Enthalpies= -606.314299
Sum of electronic and thermal Free Energies= -606.370689

Single point CPCM(oDFB) corrected:

Sum of electronic and zero-point Energies= -606.331284
Sum of electronic and thermal Energies= -606.316555
Sum of electronic and thermal Enthalpies= -606.315610
Sum of electronic and thermal Free Energies= -606.372237

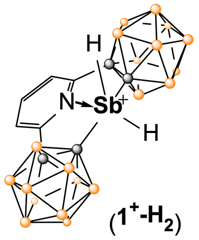

(1^{+}-H_2) :

N	1.08166	-0.16563	0.00000
C	-0.74096	0.01263	2.07730
C	0.85943	-0.01545	2.38141
C	1.73104	-0.13650	1.17241
C	3.12389	-0.15376	1.21366
H	3.64103	-0.13213	2.17182
C	3.81858	-0.18058	0.00000
H	4.90935	-0.19779	0.00000
B	0.01268	1.50948	2.49476
H	0.21327	2.28623	1.61474
B	-1.58668	1.05194	3.13188
H	-2.53463	1.62558	2.69280
B	-1.64115	-0.72915	3.31731
H	-2.62639	-1.32462	3.00459
B	-0.07396	-1.41654	2.81534
H	0.06302	-2.37509	2.12220
B	1.24972	1.01921	3.68887
H	2.30015	1.57665	3.64028
B	-0.32141	1.69345	4.21728
H	-0.39669	2.78933	4.67600
B	-1.35151	0.31544	4.73787
H	-2.18523	0.43040	5.57952
B	-0.41515	-1.20636	4.53089
H	-0.56146	-2.17096	5.21281
B	1.19461	-0.76009	3.88401
H	2.20897	-1.37794	3.96294
B	0.40323	0.29678	5.07960
H	0.84908	0.39939	6.17884
C	-0.74096	0.01263	-2.07730
C	0.85943	-0.01545	-2.38141
C	1.73104	-0.13650	-1.17241
C	3.12389	-0.15376	-1.21366

H	3.64103	-0.13213	-2.17182
B	-0.07396	-1.41654	-2.81534
H	0.06302	-2.37509	-2.12220
B	-1.64115	-0.72915	-3.31731
H	-2.62639	-1.32462	-3.00459
B	-1.58668	1.05194	-3.13188
H	-2.53463	1.62558	-2.69280
B	0.01268	1.50948	-2.49476
H	0.21327	2.28623	-1.61474
B	1.19461	-0.76009	-3.88401
H	2.20897	-1.37794	-3.96294
B	-0.41515	-1.20636	-4.53089
H	-0.56146	-2.17096	-5.21281
B	-1.35151	0.31544	-4.73787
H	-2.18523	0.43040	-5.57952
B	-0.32141	1.69345	-4.21728
H	-0.39669	2.78933	-4.67600
B	1.24972	1.01921	-3.68887
H	2.30015	1.57665	-3.64028
B	0.40323	0.29678	-5.07960
H	0.84908	0.39939	-6.17884
Sb	-1.31844	-0.27421	0.00000
H	-1.73194	-1.90627	0.00000
H	-2.74694	0.65345	0.00000

Gas Phase:

Sum of electronic and zero-point Energies= -915.165656
Sum of electronic and thermal Energies= -915.141917
Sum of electronic and thermal Enthalpies= -915.140972
Sum of electronic and thermal Free Energies= -915.213827

H_2:

H	0.00000	0.00000	0.37581
H	0.00000	0.00000	-0.37581

Gas Phase:

Sum of electronic and zero-point Energies= -1.167746
Sum of electronic and thermal Energies= -1.165385
Sum of electronic and thermal Enthalpies= -1.164441
Sum of electronic and thermal Free Energies= -1.179255

6. References

1. K. P. Anderson, H. A. Mills, C. Mao, K. O. Kirlikovali, J. C. Axtell, A. L. Rheingold, A. M. Spokoyny, *Tetrahedron* **2019**, *75*, 187–191. DOI: 10.1016/j.tet.2018.11.040.

2. S. J. Connelly, W. Kaminsky, D. M. Heinekey, *Organometallics* **2013**, *32*, 7478−7481. DOI: 10.1021/om400970j.

3. Bruker (2019). APEX III. Bruker AXS Inc., Madison, Wisconsin, USA.

4. Bruker (2013). SAINT v8.34A. Bruker AXS Inc., Madison, Wisconsin, USA.

5. Bruker (2014/5). Sadabs, 2014/5. Bruker AXS Inc., Madison, Wisconsin, USA.

6. O. V. Dolomanov, L. J. Bourhis, R. J. Gildea, J. A. K. Howard, H. Puschmann, *J. Appl. Crystallogr.* **2009**, *42*, 339−341. DOI: 10.1107/S0021889808042726.

7. G. M. Sheldrick, *Acta Cryst. A* **2015**, *71*, 3−8. DOI: 10.1107/S2053273314026370.

8. G. M. Sheldrick, *Acta Cryst. C* **2015**, *71*, 3–8, DOI: 10.1107/S2053229614024218.

9. Bruker, *SAINT*, *V8.40B*, Bruker AXS Inc., Madison, Wisconsin, USA.

10. L. Krause, R. Herbst-Irmer, G. M. Sheldrick, D. Stalke, *J. Appl. Cryst.* **2015**, *48*, 3–10, DOI: 10.1107/S1600576714022985.

11. H. Fang, Q. He, G. Liu, Z. Huang, *Org. Lett.* **2020**, *22*, 9298–9302. DOI: 10.1021/acs.orglett.0c03530.

12. Gaussian 09, Revision D.01, M. J. Frisch, G. W. Trucks, H. B. Schlegel, G. E. Scuseria, M. A. Robb, J. R. Cheeseman, G. Scalmani, V. Barone, B. Mennucci, G. A. Petersson, H. Nakatsuji, M. Caricato, X. Li, H. P. Hratchian, A. F. Izmaylov, J. Bloino, G. Zheng, J. L. Sonnengerg, M. Hada, M. Ehara, K. Toyota, R. Fukuda, J. Hasegawa, M. Ishida, T. Nakajima, Y. Honda, O. Kitao, H. Nakai, T. Vreven, Jr, J. A. Montgomery, J. E. Peralta, F. Ogliaro, M. Bearpark, J. J. Heyd, E. Brothers, K. N. Kudin, V. N. Staroverov, T. Keith, R. Kobayashi, J. Normand, K. Raghavachari, A. Rendell, J. C. Burant, S. S. Iyengar, J. Tomasi, M. Cossi, N. Rega, N. J. Millam, M. Klene, J. E. Knox, J. B. Cross, V. Bakken, C. Adamo, J. Jaramillo, R. Gomperts, R. E. Stratmann, O. Yazyev, A. J. Austin, R. Cammi, C. Pomelli, J. W. Ochterski, R. L. Martin, K. Morokuma, V. G. Zakrzewski, G. A. Voth, P. Salvador, J. J. Dannenberg, S. Dapprich, A. D. Daniels, Ö. Farkas, J. B. Foresman, J. V. Ortiz, J. Cioslowski, D. J. Fox, Gaussian, Inc., Wallingford CT, (2010).

13. J. P. Perdew, *Phys. Rev. B* **1986**, 33, 8822–8824. DOI: 10.1103/PhysRevB.33.8822.

14. A. D. Becke, *Phys. Rev. A* **1988**, 38, 3098–3100. DOI: 10.1103/PhysRevA.38.3098.

15. F. Weigend, R. Ahlrichs, *Phys. Chem. Chem. Phys.* **2005**, 7, 3297–3305. DOI: 10.1039/B508541A.

16. A. Bergner, M. Dolg, W. Küchle, H. Stoll, H. Preuß, *Mol. Phys.*, **1993**, 80, 1431–1441.

DOI: 10.1080/00268979300103121.
17. T. H. Dunning, P. J. Hay, 1977, vol. 3. Ed. Schaefer III, H. F. pp. 1-28, Plenum Press, New York.
18. V. Barone, M. Cossi, *J. Phys. Chem. A* **1998**, *102*, 1995–2001. DOI: 10.1021/jp9716997.
19. M. Cossi, N. Rega, G. Scalmani, V. Barone, *J. Comput. Chem.* **2003**, *24*, 669-681. DOI: 10.1002/jcc.10189.
20. M. Bursch, T. Gasevic, J. B. Stückrath, S. Grimme, *Inorg. Chem.* **2021**, *60*, 272–285. DOI: 10.1021/acs.inorgchem.0c02907.
21. F. Neese, *Comput. Molec. Sci.* **2025**, 15, e70019. DOI: 10.1002/wcms.70019
22. S. Grimme, J. Antony, S. Ehrlich, H. Krieg, *J. Chem. Phys.* **2010**, 132, 154104. DOI: 10.1063/1.3382344.
23. G. L. Stoychev, A. A. Auer, R. Izsak, F. Neese, *J. Chem. Theory Comput.* **2018,** 14, 619-637. DOI: 10.1021/acs.jctc.7b01006.
